# Electrically Tunable Graphene‐Metal Hybrid Metasurfaces for High‐Efficiency, Broadband Mid‐Infrared Transmission Modulation

**DOI:** 10.1002/advs.77550

**Published:** 2026-09-03

**Authors:** Heonhak Ha, Jinseok Kong, Junhyung Kim, Jiwon Kang, Min Seok Jang

**Affiliations:** ^1^ School of Electrical Engineering Korea Advanced Institute of Science and Technology Daejeon Republic of Korea

**Keywords:** broadband, electro‐optic modulator, equivalent circuit, graphene, nanodevice, nanophotonics, nanostructure fabrication, optical efficiency, optoelectronics, spectrometer

## Abstract

Transmissive active metasurfaces are essential for ultra‐compact on‐chip optical systems and are widely used in sensing, imaging, and spectroscopy. However, these devices generally suffer from lower efficiency and greater design complexity compared to their reflective counterparts. In this work, an electrically tunable transmission filter based on single‐layer graphene integrated with a capacitive gold grating on a mid‐IR transparent CaF_2_ substrate is demonstrated. By employing an ionic gel gating scheme to induce a high Fermi level in graphene, the device achieves a high transmission modulation efficiency of 73.3% for TM‐polarized light, along with a broad operational bandwidth of 742 cm^−1^. Furthermore, by modifying the device structure, an ultrawide operational bandwidth of 1949 cm^−1^ can be achieved at the expense of reducing the modulation efficiency to 44%, outperforming previously reported electrically tunable mid‐IR transmissive metasurfaces. Equivalent circuit model analysis reveals that this extraordinary modulation and bandwidth arise from the dynamic formation of a parallel LC resonance between the tunable inductive response of highly doped graphene and the metal grating's capacitive response. Furthermore, leveraging the spectral diversity generated by this Fermi‐level modulation, the platform is computationally demonstrated to operate as a single‐pixel mid‐IR spectrometer. These results provide a platform for ultra‐compact mid‐IR applications.

## Graphical abstract

A graphene‐metal hybrid transmissive metasurface enables electrically tunable mid‐infrared transmission modulation through ionic gel top gating. By combining graphene with a metallic slit metasurface, high modulation efficiency and broadband operation are achieved simultaneously, demonstrating efficient electrical control of mid‐infrared transmission over a broad spectral range.

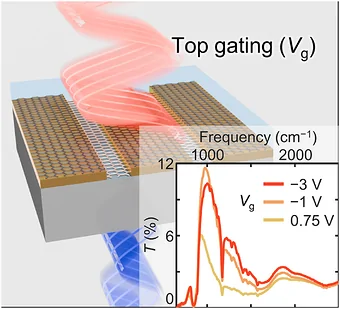

## 1 Introduction

Active manipulation of mid‐infrared (mid‐IR) light is crucial for next‐generation optoelectronic applications, including molecular sensing, thermal imaging, and free‐space communication [1, 2, 3, 4]. While metasurfaces have provided a versatile platform for mid‐IR optics, achieving dynamic control remains a challenge. Thermally reconfigurable metasurfaces, for instance, inherently suffer from slow response times, high power consumption, and severe thermal crosstalk [5, 6].

Electrically modulated metasurfaces, in contrast, offer rapid, continuous tuning and better energy efficiency, making them highly favorable for ultra‐compact integration [5, 7, 8, 9]. Among various electrically tunable materials, graphene‐based metasurfaces have attracted significant attention due to graphene's electrically controllable carrier concentration, broadband optical response in the mid‐IR regime, and excellent thermal and electrical stability [10, 11, 12, 13]. In addition, graphene's atomic thickness, mechanical robustness, and compatibility with planar fabrication processes enable ultra‐compact and scalable device designs [14, 15, 16]. As a result, active graphene‐based metasurfaces have been extensively investigated for mid‐IR applications, demonstrating tunable amplitude, phase, and polarization control. These devices enable a wide range of functionalities, including beam steering for LiDAR applications [17, 18], biochemical sensing platforms [19, 20], holographic applications [21], and imaging systems [22, 23], highlighting the versatility of graphene‐based metasurfaces for next‐generation mid‐IR photonic devices.

However, the atomically thin nature of graphene leads to inherently weak light‐matter interaction. To overcome this, conventional active metasurfaces heavily rely on resonance overlap within reflective architectures, such as Salisbury screen‐like Fabry‐Pérot cavities [24, 25]. While these reflective devices enhance modulation, they limit practical optical system designs. They require bulky beam‐splitting configurations, suffer from reduced numerical apertures, and present critical challenges for monolithic integration with essential on‐chip components like focal plane array detectors or surface‐emitting lasers [26, 27, 28, 29, 30]. Despite the practical advantages of transmissive architectures, developing active mid‐IR metasurfaces that simultaneously provide high modulation efficiency and broadband spectral tunability remains an unresolved challenge. Fundamentally, this difficulty arises from the intricate nature of multi‐port scattering processes. While reflective architectures typically function as single‐port systems where the incident wave is either absorbed or coupled back into the reflection channel, transmissive devices must simultaneously control the scattering pathways to both forward and backward radiation ports [31, 32].

In this work, we propose and experimentally demonstrate a dynamically tunable mid‐IR transmissive metasurface that simultaneously achieves high transmission modulation efficiency and a broad operational bandwidth based on single‐layer graphene coupled to a gold grating structure. The device utilizes a parallel LC resonance between the strong capacitive response of the metallic slit array and the tunable inductive response of graphene electrostatically controlled via ionic‐gel gating. Unlike devices based on graphene plasmon resonances, which are fundamentally sensitive to graphene quality and highly spectrally selective, this tunable LC resonance mechanism enables a higher transmission modulation efficiency of 73.3% alongside a broader full‐width at half‐maximum (FWHM) operational bandwidth of 742 cm^−1^, outperforming previous electrically tunable mid‐IR transmissive metasurfaces [33, 34, 35]. Furthermore, by modifying the device structure, an ultrawide operational bandwidth of 1949 cm^−1^ can be achieved at the expense of reducing the modulation efficiency to 44%. Finally, we demonstrate that the combination of high transmission modulation efficiency and broad operational bandwidth provides sufficient spectral diversity to computationally reconstruct unknown incident light, thereby showcasing the device's potential as a highly compact, single‐chip mid‐IR spectrometer.

## 2 Results

### 2.1 Device Geometry and Fabrication

**FIGURE 1:**
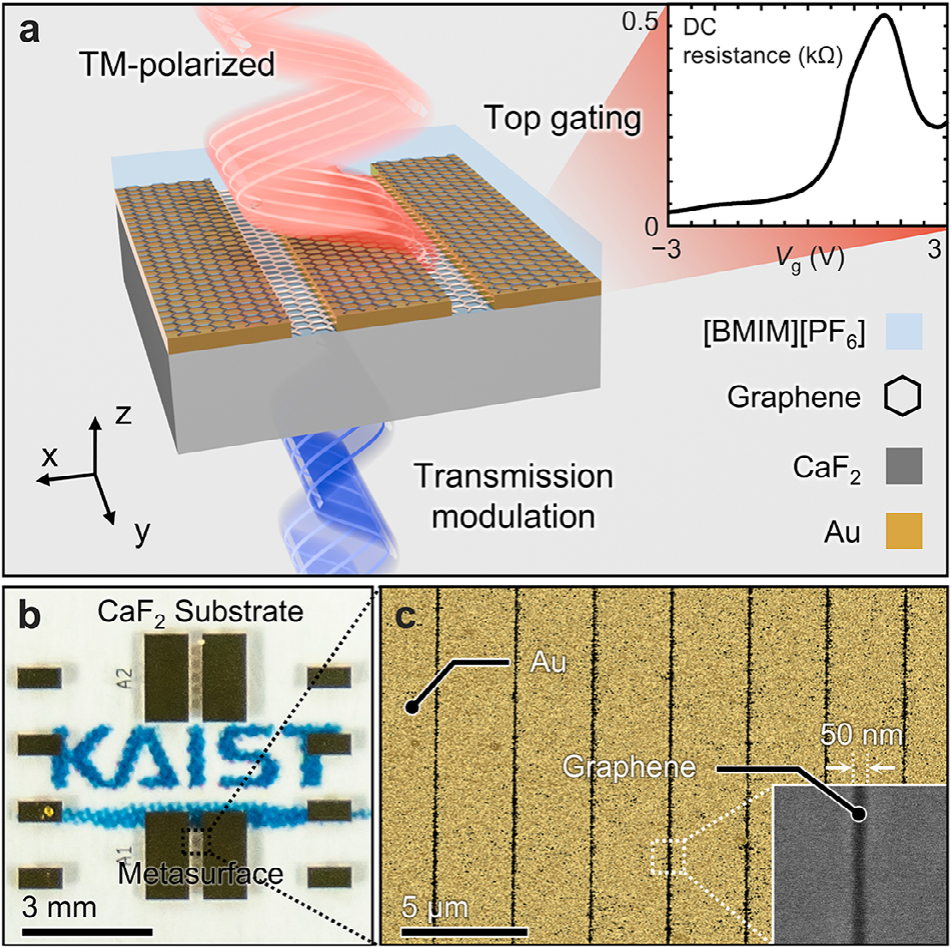
Active metasurface for high‐efficiency transmission modulation. (a) Schematic of the active transmission modulation metasurface. The device consists of a 500‐µm‐thick CaF_2_ substrate, a periodic gold grating (10‐nm height with a 2‐nm‐thick Ti adhesion layer), a transferred graphene layer, and a spin‐coated ionic gel. A gate voltage (*V*_g_) was applied through the ionic gel layer. The inset graph represents DC resistance of the graphene layer as a function of *V*_g_, demonstrating effective ionic gel gating. (b) Top‐view image of the fabricated device. Scale bar represents 3 mm. (c) Top‐view false‐colored SEM image of the device (*p* = 3 µm, *g* = 50 nm) with an enlarged inset of the pattern. Scale bar represents 5 µm.

A schematic of the proposed device is shown in Figure 1a. A periodic gold grating structure with a grating period *p* ∈ [1, 5]µm and a gap width *g* ∈ [50,  150]nm was patterned on top of a 500 µm‐thick CaF_2_ substrate via thermal evaporation and electron beam lithography. The CaF_2_ substrate is transparent from the visible to the mid‐IR regime, down to approximately 800 cm^−1^ [36]. The thickness of the gold grating, *h*, is fixed at 10 nm, corresponding to the minimum stable thickness achievable with our fabrication process. Monolayer graphene grown by chemical vapor deposition, wet‐transferred onto the patterned structure, covers both the gold strips and the exposed substrate within the gaps [37]. Finally, a 2.98 µm‐thick layer of ionic gel [BMIM][PF_6_] was spin‐coated onto the graphene layer, allowing for electro‐optic modulation of graphene carrier concentration. Figure 1b presents a top‐view image of the fabricated device. The two large gold pads serve as the source and drain electrodes of the device. Five gold grating patterns are located in the channel region between the source and drain. The source and drain pads are connected to the PCB substrate through wire bonding. A corresponding SEM image is shown in Figure 1c, confirming the successful fabrication of a structure with *p* = 3 µm and *g* = 50 nm. Detailed fabrication steps are described in the Methods section and Note S1, and statistical analysis across multiple identical devices is provided in Note S2.

To dynamically tune the Fermi level of graphene *E*_F_, a gate voltage *V*_g_ was applied through the ionic gel layer. The inset graph in Figure 1a shows the DC resistance of the graphene channel as a function of *V*_g_. The charge neutrality point (CNP), where the resistance becomes maximized, occurs between +0.5 and +1.5 V depending on the samples, meaning that the graphene is p‐doped at zero gate bias. During the optical measurement, *V*_g_ was modulated from the CNP (∼ +1 V) to a highly hole‐doped regime (up to −3 V) to obtain maximum carrier density modulation (see Note S3). We note that both the electrical and optical responses of the device were not symmetrical with respect to the CNP, likely due to the intrinsic difference between cations and anions in the ionic gel [38, 39].

### 2.2 Transmission Modulation in the Mid‐IR Regime and the Equivalent Circuit Model Analysis

**FIGURE 2:**
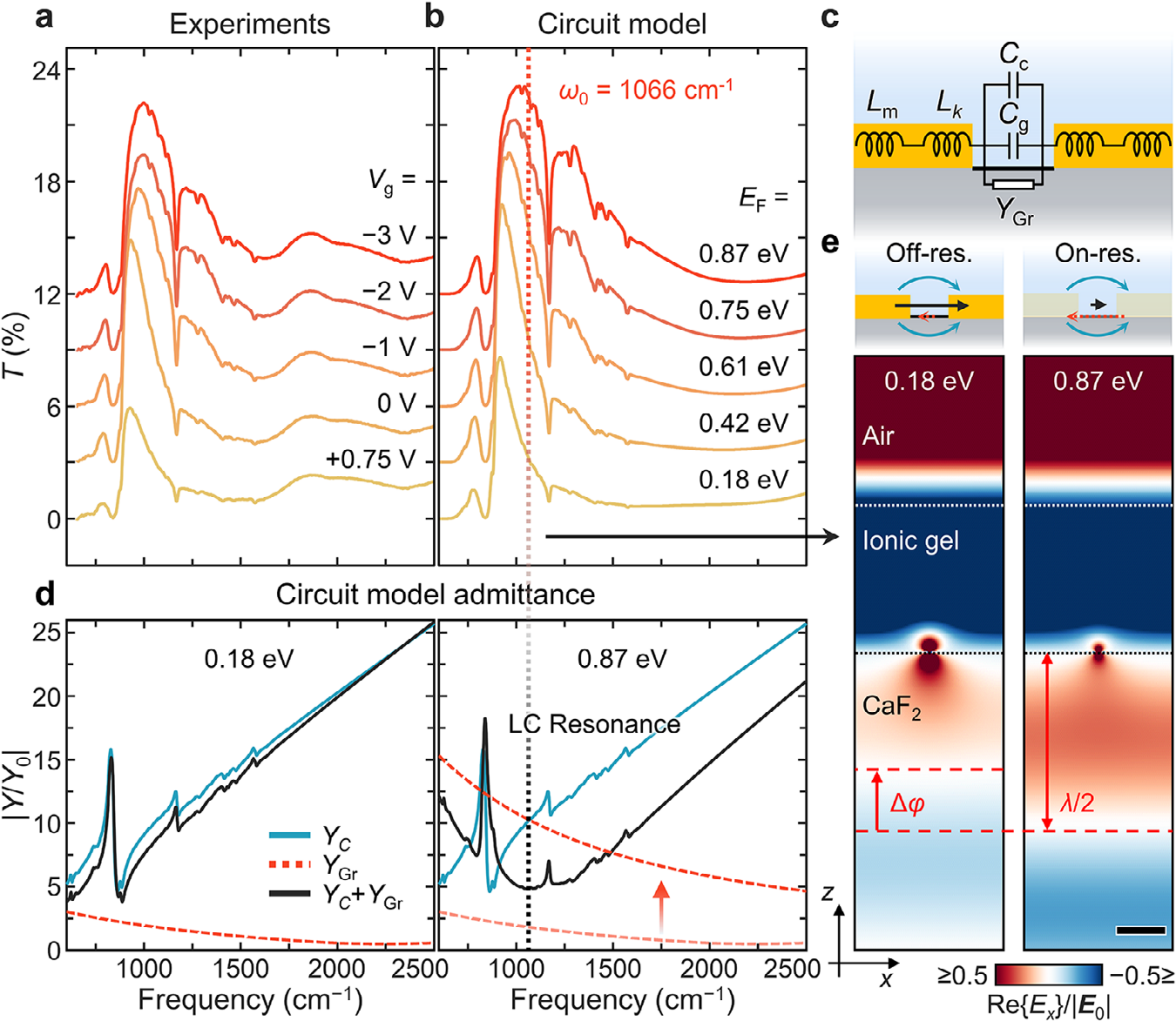
Electrostatic modulation of transmission and analysis based on a circuit model. (a) Measured and (b) calculated transmission spectra of the device (*p* = 3 µm, *g* = 50 nm) for TM‐polarized light. The gate voltage is swept from −3 V (*E*_F_ = 0.87 eV) to +0.75 V (CNP, 0.18 eV). (c) Schematic of the equivalent circuit model. (d) Normalized admittance magnitudes |*Y*/*Y*_0_| for the capacitive metal antenna (*Y_C_*, light blue), the graphene layer (*Y*_Gr_, red dashed line), and their sum (*Y_C_* +*Y*_Gr_, black) at the CNP and *E*_F_ = 0.87 eV. The dashed black line in d highlights the intersection between |*Y_C_* | and |*Y*_Gr_|, corresponding to the LC parallel resonance at *ω*_0_ = 1066 cm^−1^. (e) Schematics of the effective current flow (upper panels) and cross‐sectional distributions of the normalized electric field Re{***E*** *x*}/| ***E***_0_| (lower panels) at *ω*_0_ = 1066 cm^−1^. The left and right columns represent the off‐resonance (CNP, 0.18 eV) and on‐resonance (*E*_F_ = 0.87 eV) states, respectively. Scale bar represents 1 µm. The transmission spectra in Figure 2a and b are vertically offset by a constant value for enhanced visualization.

The devices were measured using an infrared microscope coupled to an FTIR spectrometer in transmission mode under two orthogonal linearly polarized illuminations. The numerical aperture (NA) of the IR microscope is 0.71, corresponding to a collection angle of approximately 45 degrees. The measured transmittance spectra of the device for TM‐polarized light with *p* = 3 µm and *g* = 50 nm, as *V*_g_ was varied from −3 V to +0.75 V, are depicted in Figure 2a. Regardless of the polarization or the applied gate voltage, strong absorption dips appear in all spectra near 840 and 1170 cm^−1^, arising from the absorption lines of the ionic gel as well as the suppressed transmission due to the increasing loss of the CaF_2_ substrate at frequencies below 1200 cm^−1^ (see Note S4). In the case of TE‐polarized incident light, whose electric field oscillates parallel to the grating lines, the transmittance is primarily determined by the gap‐to‐period ratio and thus marginally dependent on *V*_g_, as illustrated in Figure S11. Unlike TM‐polarized light, the electric field does not oscillate across the narrow gaps and therefore cannot efficiently induce charge accumulation at the gap edges. As a result, the strong capacitive response associated with the gap structure is not excited, suppressing the LC resonance mechanism responsible for transmission modulation. Consequently, the transmission spectra exhibit negligible modulation under TE‐polarized illumination. On the other hand, for TM‐polarized illumination, whose electric field is oscillating perpendicular to the grating lines, the transmission spectra show a strong dependence on *V*_g_. Despite the background losses from the ionic gel and CaF_2_, the transmission peak exhibits a clear blueshift as the gate voltage moves away from the CNP (*V*_g_ = +0.75 V). The maximum modulation efficiency of *η_T_* = 1 − (*T*_min_/*T*_max_) = 73.3% occurs at *ω* = 1265 cm^−1^, where *T*_max_ and *T*_min_ are 5.3% and 1.4%, respectively. At *ω* = 933 cm^−1^, the transmittance *T* increased from 5.9% (*V*_g_ = +0.75 V) to 11.9% (*V*_g_ = −1 V), resulting in a maximum transmission difference of *T*_max_ − *T*_min_ = 6%. We note that the maximum transmission difference can be further enhanced to 17.1% for a device with a larger opening ratio, *p* = 1 µm and *g* = 50 nm, as explained further in the following sections.

To theoretically analyze these results, we employed an equivalent circuit model. This simulation result (shown in Figure 2b) successfully reproduces the experimentally observed transmission modulation behavior shown in Figure 2a. The applied gate voltages ranging from +0.75 to −3 V were fitted to Fermi levels from 0.18 to 0.87 eV, considering the capacitance formula. Here, the graphene carrier scattering rate Γ was fixed at 0.066 eV (62.85 fs), yielding a graphene mobility of 115 cm^2^/V∙s at 0.87 eV, a value comparable to those reported in previous studies on ionic gel‐gated graphene [40, 41]. The detailed fitting process is provided in Note S7. A deviation between the experimental and calculated spectra is observed at frequencies above 2000 cm^−1^, where a broad spectral feature appears in the measurement. This feature originates from the coupling to the waveguide modes supported within the ionic gel layer, averaged out over a broad illumination angle distribution associated with the high NA (0.71) IR microscope objective (a detailed discussion is provided in Notes S8 and S9).

Our model treats the metasurface as a thin homogeneous layer with an effective sheet admittance *Y*_s_, recognizing that the metasurface is a few orders of magnitude thinner than the wavelength of incident light, *λ* ≫ *h* = 10 nm. The transmission coefficient of the metasurface is then given by *t*_s_ = 2*Y*_Sup_/(*Y*_Sup_+*Y*_Sub_+*Y*_s_), where $Y_{\mathrm{Sup}}=\sqrt{\varepsilon_{\mathrm{Sup}}/\mu_{\mathrm{Sup}}}$ and $Y_{\mathrm{Sub}}=\sqrt{\varepsilon_{\mathrm{Sub}}/\mu_{\mathrm{Sub}}}$ are the characteristic admittances of the superstrate (ionic gel) and the substrate (CaF_2_), respectively. This coefficient is subsequently used in the transfer matrix method (TMM) to obtain the final transmittance spectrum. Accordingly, the transmission peaks observed in Figure 2b occur when |*Y*_s_| is minimized.

To determine *Y*_s_, we leveraged the aforementioned circuit model, which is valid in the quasi‐static limit. In this regime, the charge dynamics and currents induced by TM‐polarized light can be directly mapped onto electrostatic circuit elements such as inductors and capacitors, as shown in Figure 2c [42]. Consequently, the effective sheet admittance of the metasurface can then be expressed as

(1) $$\begin{array}{cc} \;Y_{s} & ={\left[\frac{1}{Y_{L}}+\frac{1}{Y_{C}+Y_{\mathrm{Gr}}}\right]}^{-1}\\ & ={\left[\frac{-i\omega \left(L_{m}+L_{k}\right)}{p}+\frac{1}{-i\omega p\left(C_{c}+C_{g}\right)+\frac{p}{g}\sigma_{\mathrm{Gr}}}\right]}^{-1}\; \end{array}$$

where *Y_L_* and *Y_C_* represent the surface admittances associated with the inductive and capacitive responses of the metal antenna, respectively. The inductive response of the metal is decomposed into magnetic (*L*_m_) and kinetic (*L*_k_) inductances, while the capacitive response comprises coplanar (*C*_c_) and gap (*C*_g_) capacitances. These components are determined by the structural parameters and the material properties, as detailed in Note S10. The contribution of graphene is represented by its surface admittance *Y*_Gr_, determined by the optical sheet conductivity *σ*_Gr_, and electrically connected in parallel with *Y_C_*.

The expression for *Y*_s_ can be further simplified by neglecting marginal contributions. Considering the large period‐to‐gap ratio (*g* ≪ *p*) and the ultrathin nature of the slab (*h* ≪ *g*, *λ*_0_), the magnitudes of *L*_m_ and *C*_g_ (proportional to *h*) become negligible compared to *L*_k_ (inversely proportional to *h*) and *C*_c_ (independent of *h*). Furthermore, in the frequency range (*ω* < 1300 cm^−1^) where the primary modulation occurs, the impedance contribution of *L*_k_ is much lower than that of *C*_c_, indicating that the metal grating behaves predominantly as a capacitor in this regime (see Note S11). Within this approximation, *Y*_s_ can be greatly simplified to

(2) $$Y_{s}≅Y_{C}+Y_{\mathrm{Gr}}≅-pi\omega C_{c}+\frac{p}{g}\sigma_{\mathrm{Gr}}.$$

The physical origin of the transmission can be understood from the interaction between these two admittance components *Y_C_* and *Y*_Gr_. Under TM‐polarized normal incidence, the oscillating electric field is concentrated across the narrow metal gaps, inducing charge accumulation at the gap edges and generating a capacitive displacement current represented by *Y_C_*. As the Fermi level increases, the higher carrier density in graphene provides an additional inductive current path represented by *Y*_Gr_, which is out of phase with the displacement current and partially cancels the capacitive response. Consequently, the effective surface admittance *Y*_s_ is reduced. Since the transmission coefficient is inversely related to |*Y*_s_|, a reduction in |*Y*_s_| leads to enhanced transmission.

The interplay between *Y_C_* and *Y*_Gr_ induces the transmission modulation behavior. Within the operating frequency range (*ω* = 1265 cm^−1^ = 0.157 eV) and at the high Fermi level (|*E*_F_| > 0.4 eV) achieved by ionic gel gating, graphene's interband transitions are effectively suppressed due to Pauli blocking (2|*E*_F_| ≫ *ħω*). Under these conditions, graphene exhibits a Drude‐like free‐carrier response expressed by σ_Gr_(ω) ≈ σ_intra_ (ω) = *e* ^2^ *E*_F_/πℏ^2^(Γ − *i*ω) [43, 44, 45]. Since the operating frequency exceeds the assumed scattering rate (Γ = 0.066 eV), Im(*σ*_intra_) dominates over Re(*σ*_intra_) and scales inversely with frequency. Figure 2d shows *Y*_Gr_ and *Y_C_* at the CNP and *E*_F_ = 0.87 eV. At the CNP condition, *Y_C_* ≫ *Y*_Gr_ throughout the entire spectral range due to the low carrier concentration of graphene. *Y*_s_ ≈ *Y_C_* is linearly dependent on *ω* where the surrounding materials are nondispersive but shows a strong dispersion near the major absorption line of the ionic gel (*ω* = 840 cm^−1^). As a result, |*Y*_s_| has its minimum value at around *ω* ≈ 870 cm^−1^ where the corresponding |*t*_s_| becomes maximized. Note that the peak of |*t*_s_| at the CNP is obscured in the total transmittance of the structure because of the absorption from the ionic gel and CaF_2_.

As *E*_F_ increases, the inductive *Y*_Gr_ is no longer negligible and starts to cancel the capacitive *Y_C_*, as illustrated in Figure 2d. Consequently, overall |*Y*_s_| decreases, leading to increased transmittance over a wide spectral range. For *E*_F_ = 0.87 eV, |*Y*_s_| is minimized at *ω*_0_ = 1066 cm^−1^, where the cancellation between *Y*_Gr_ and *Y*_C_ becomes optimal, forming a parallel LC resonance. At this LC resonance, |*Y*_s_| is reduced to 60% compared to its off‐resonance value at the CNP. Since |*Y*_s_| is significantly larger than |*Y*_Sup_| ≈ |*Y*_Sub_| ≈ 1.3*Y*_0_ and thus primarily dictates |*t*_s_| near the resonance point, the transmittance is expected to increase by a factor of approximately 2.8 (≈ 1/0.6^2^). The spectrum at *E*_F_ = 0.87 eV in Figure 2b confirms this, showing a similar increment in maximum transmittance, albeit with a slight frequency shift due to ionic gel absorption around *ω* = 1170 cm^−1^. To clearly isolate the transmission peak shifts caused by the LC resonance, we performed an additional circuit model analysis for an ideal, lossless case (see Note S12). In the absence of superstrate and substrate losses, the transmission peak shift is more distinctly observed, substantiating that our mechanism is robust. We emphasize that although graphene plasmons are crucial in various mid‐IR active metasurfaces [16, 34, 42, 46, 47, 48, 49], our device does not rely on this mechanism. For the gap size of 50 nm, the graphene plasmon resonance occurs around *ω* = 3202 cm^−1^, and its contribution to the transmission spectra is also marginal (see Note S13).

Figure 2e presents simulated *E_x_* field profiles under on‐ and off‐resonance conditions. In the off‐resonance state, the transmission is suppressed due to the presence of the capacitive gold grating. In contrast, when the system is on‐resonance, the parallel LC resonance between the capacitive gold grating and the graphene suppresses induced currents. Consequently, the incident wave experiences minimal interaction with the metasurface, leading to a transmitted wave that is relatively unperturbed compared to the off‐resonance case. We also note that, as the metasurface becomes less capacitive, the on‐resonance transmitted wave has a phase lead over the off‐resonance case.

### 2.3 Transmission Modulation Dependent on the Geometric Parameters

**FIGURE 3:**
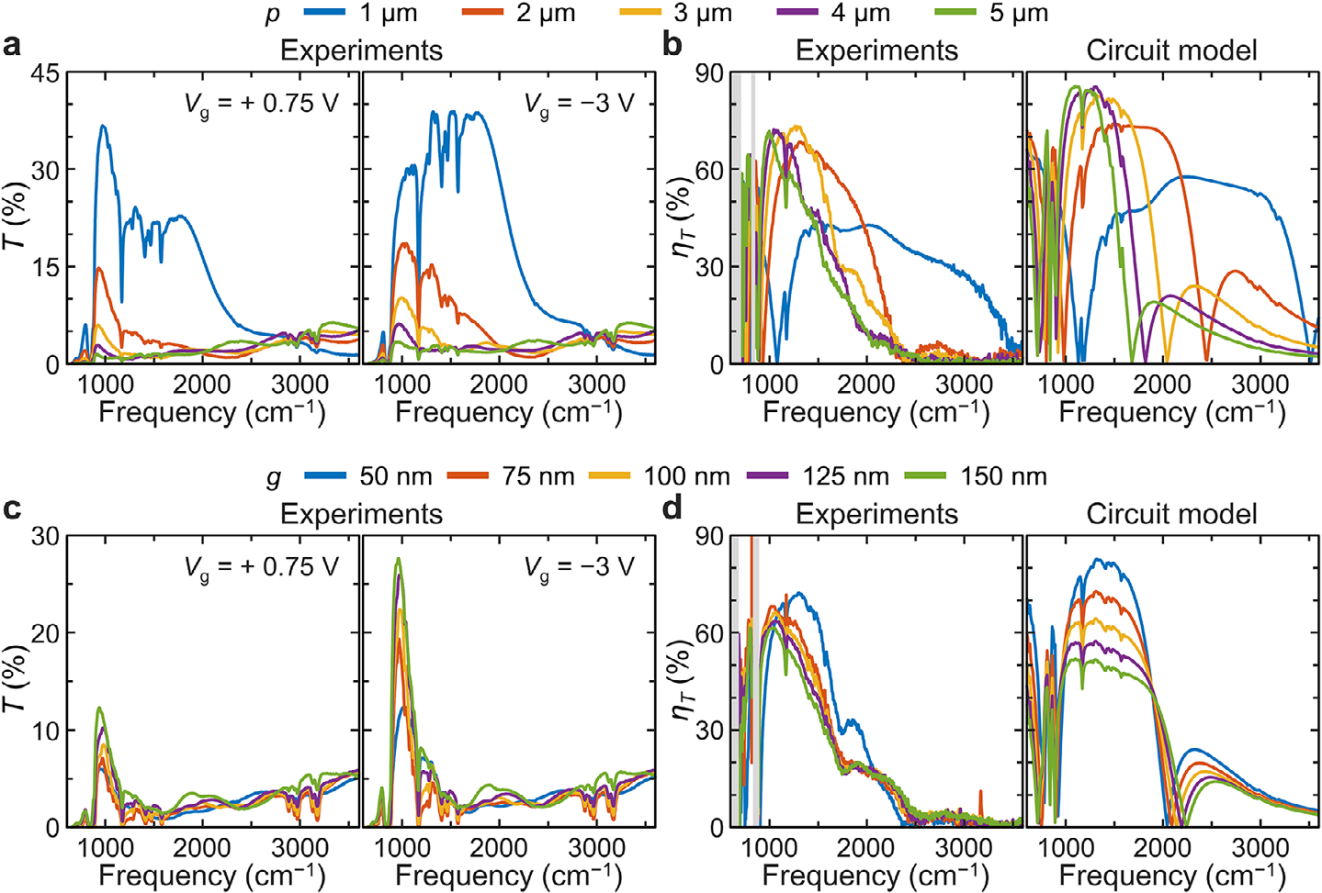
Measured transmission spectra and transmission modulation efficiency for devices with varying geometrical parameters. (a) Transmission spectra measured at *V*_g_ = +0.75 V (left) and −3 V (right) for devices with periods ranging from 1 to 5 µm and a fixed gap size of 50 nm. (b) Measured (left) and simulated (right) transmission modulation efficiency *η_T_* as a function of frequency for the period sweep, calculated from the transmission spectra at *V*_g_ = +0.75 V (*E*_F_ = 0.18 eV) and −3 V (*E*_F_ = 0.87 eV). (c) Transmission spectra measured at *V*_g_ = +0.75 V (left) and −3 V (right) for devices with gap sizes ranging from 50 to 150 nm and a fixed period of 3 µm. (d) Measured (left) and simulated (right) transmission modulation efficiency *η_T_* as a function of frequency for the gap sweep, calculated from transmission spectra at *V*_g_ = +0.75 V (*E*_F_ = 0.18 eV) and −3 V (*E*_F_ = 0.87 eV). The gray shaded region indicates data that are masked because *T*_max_ is close to zero, rendering the data meaningless.

The transmission modulation characteristics of the metasurface can be tailored by altering the geometrical parameters of the device. Figure 3 presents the measured transmission spectra and transmission modulation efficiency, *η_T_* = 1 − (*T*_min_/*T*_max_), as functions of period *p* (Figure 3a, b) and gap size *g* (Figure 3c, d). First, Figure 3a shows the transmission spectra at the CNP and *V*_g_ = −3 V for devices with *p* ranging from 1 µm to 5 µm while *g* is fixed at 50 nm. The overall transmission increases as *p* decreases owing to the increased opening ratio, *g*/*p*, which allows more light to pass through the structure. In addition, the transmission peak frequency at *V*_g_ = −3 V progressively blueshifts from 960 to 1529 cm^−1^ with decreasing *p*.

The key reason is that the metasurface becomes less capacitive with decreasing *p*. As discussed earlier, at high Fermi energy (*E*_F_ = 0.87 eV), the transmission peak occurs when *Y*_Gr_ cancels *Y_C_*. As *p* decreases, *Y_C_* decreases relative to *Y*_Gr_, leading to the parallel LC resonance occurring at a higher frequency. In contrast, at low Fermi energy (*E*_F_ = 0.18 eV), the system is predominantly capacitive regardless of *p*, and so the transmission peak is pinned around *ω* ≈ 870 cm^−1^.

These transmission changes are directly reflected in the transmission modulation efficiency spectra shown in Figure 3b, where the theoretical efficiency calculated using the equivalent circuit model shows good agreement with the experimental data. From the transmission modulation efficiency spectra, the operational bandwidth can be defined as the frequency range at which the modulation efficiency decreases by 50% from its maximum value [33]. As *p* decreases, the operational bandwidth increases substantially from 629 to 1949 cm^−1^. This broadening results from the increasing spectral separation between the transmission peaks of low and high Fermi energies. However, the average modulation efficiency gradually decreases because the larger opening ratio increases the background transmission and thereby reduces the relative contrast between *T*_min_ and *T*_max_. Nevertheless, the absolute transmission difference, Δ*T* = *T*_max_ − *T*_min_, remains substantial and even increases for shorter periods owing to the higher optical throughput. For example, the *p* = 1 µm, *g* = 50 nm device exhibits an absolute transmission difference of approximately 17.1% (*T*_max_ = 38.9%, *T*_min_ = 21.8% at *ω* = 1530 cm^−1^) together with the broadest modulation bandwidth of 1949cm^−1^, whereas the device exhibiting the highest modulation efficiency (*p* = 3 µm, *g* = 50 nm, *η_T_* = 73.3%) provides lower transmission throughput (*T*_max_ = 5.3%, *T*_min_ = 1.4% at *ω* = 1265 cm^−1^) and narrower bandwidth (742 cm^−1^). These results reveal a design trade‐off between modulation efficiency, operational bandwidth, and absolute transmission difference, providing flexible design guidelines for application‐specific optimization.

A similar but less dramatic effect can be achieved by adjusting the gap size *g*. Figure 3c, d shows the transmission and transmission modulation efficiency spectra, respectively, for various *g* while *p* is fixed at 3 µm. As shown in Figure 3c, increasing *g* results in higher overall transmission because of the larger opening area of the grating. In contrast, decreasing *g* causes the transmission peak to shift toward higher frequencies. Consequently, the modulation band also blueshifts and broadens, a trend that is well captured by the circuit model, as shown in Figure 3d. Although the coplanar capacitance *C*_c_ increases only logarithmically with *g* ^−1^, the graphene contribution *Y*_Gr_ scales approximately with the field concentration factor *p*/*g* and therefore becomes increasingly dominant relative to *Y_C_* at smaller gaps. This enhanced graphene response leads to the observed blueshift of the parallel LC resonance. The detailed analysis of the geometrical parameter dependence is provided in Note S14.

### 2.4 Numerical Demonstration of a Single‐Chip Spectrometer

The high modulation efficiency and broad tunability, demonstrated through both experimental measurements and circuit model analysis in the preceding sections, provide a foundation for applying our device as an on‐chip micro‐spectrometer, extending its functionality beyond that of a simple active transmission modulator. Unlike conventional spectrometers that rely on bulky dispersive optics or mechanical moving parts [50, 51, 52, 53, 54, 55], metasurface‐based on‐chip spectrometers computationally reconstruct the incident spectrum from photodetector signals collected across multiple channels with distinct frequency responses [56, 57, 58, 59]. In this study, we propose and numerically demonstrate a single‐chip spectrometer that further minimizes the device footprint by replacing spatially distributed channels with a single pixel, leveraging the spectral diversity achieved solely through Fermi level modulation of graphene.

**FIGURE 4:**
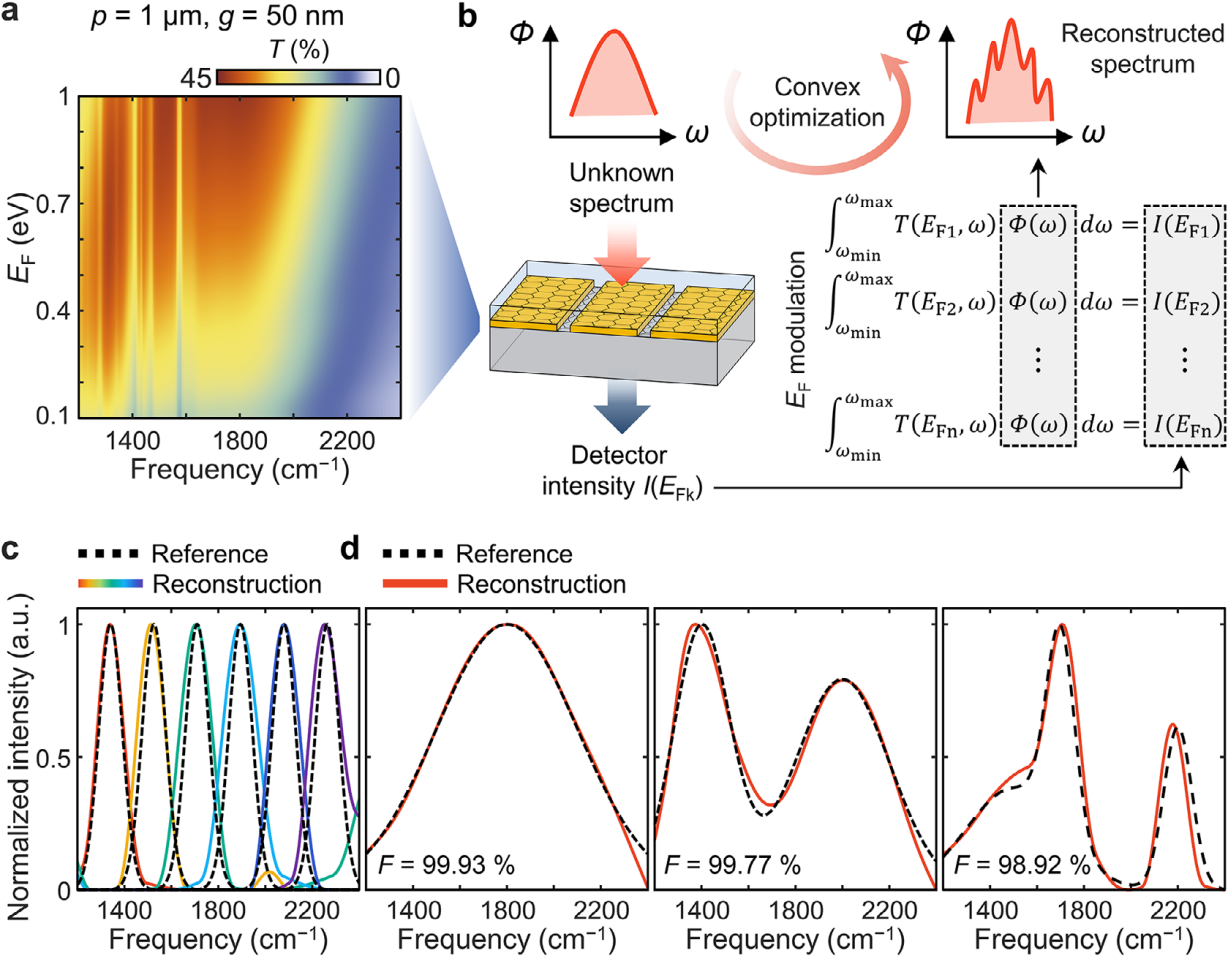
Application as a single‐chip spectrometer. (a) Transmission matrix (**T**) calculated for the optimized device with *p* = 1 µm, *g* = 50 nm, and Γ = 0.066 eV. (b) Schematic illustration of the spectral reconstruction process utilizing Fermi level modulation of graphene, based on compressed sensing (CS) theory. (c) Reconstruction results for narrowband Gaussian spectra with a standard deviation of 50 cm^−1^ at various center frequencies. Black dotted line and colored solid lines represent the reference and reconstructed spectra, respectively. (d) Reference spectra (black dashed lines) and reconstructed spectra (red solid lines) for selected broadband and complex signals composed of multiple Gaussian spectra.

Figure 4a shows the calculated transmission matrix (**T**) of the structure designed for the single‐chip spectrometer. To maximize the spectrometer's performance, transmission spectra at different Fermi levels must be distinct from one another, serving as a set of linearly independent (or quasi‐orthogonal) basis vectors. This spectral diversity allows the system to distinctly capture different spectral components of the incident light and can be enhanced by the high modulation depth of the device. We select the optimal structural parameters *p* = 1 µm and *g* = 50 nm and the frequency range *ω*_min_ = 1200 cm^−1^ to *ω*_max_ = 2400 cm^−1^, which provide a balance between frequency bandwidth and modulation depth. The transmission matrix is composed of transmission spectra obtained by sweeping the Fermi level from *E*_F_ = 0.1 eV to 1.0 eV, acting as the prior information (basis vectors) for reconstructing unknown incident spectra. All transmission spectra are calculated using RCWA under normal incidence, with the same assumed scattering rate.

Figure 4b illustrates the spectral reconstruction process for an unknown incident spectrum *Φ*(*ω*). Mathematically, the detected intensity *I* at a specific Fermi level *E*_Fk_ is expressed as the integration of the transmission spectrum *T* and the incident spectrum *Φ*:

(3) $$I\;\left(E_{\mathrm{Fk}}\right)=\int_{\omega_{\min}}^{\omega_{\max}\;}T\;\left(E_{\mathrm{Fk}},\omega \right)\Phi \left(\omega \right)d\omega .$$

Equation 3 can also be discretized into a matrix form as

(4) $$\;I_{M\times 1}=T_{M\times N}\;\Phi_{N\times 1}$$

where *M* represents the number of measurement steps, and *N* represents the number of spectral points. Given a frequency step of 1 cm^−1^ and a Fermi level step of 0.01 eV, we obtain *M* = 91 and *N* = 1201 for our reconstruction process. Generally, solving this inverse problem to reconstruct *Φ*(*ω*) is an underdetermined system (N ≫ M), which makes it difficult to obtain a unique solution. To address this issue, we introduce a regularization method based on compressed sensing (CS) theory [59, 60, 61, 62, 63, 64] as follows:

(5) $$\begin{array}{cc} & Minimize\;{\left|I-T\Phi \right|}_{2}+\sum_{i}\left(\alpha_{i}{\left|\Phi_{i}\right|}_{1}+\beta_{i}{\left|D_{1}\Phi_{i}\right|}_{1}+\gamma_{i}{\left|D_{2}\Phi_{i}\right|}_{2}\right)\\ & \times \;subject\;to\;0\leq \Phi \leq 1 \end{array}$$

where |∙|_1_ and |∙|_2_ denote the *l*_1_‐norm and *l*_2_‐norm, and D_1_ and D_2_ denote the first‐ and second‐order differential operators, respectively. The regularization terms utilizing these differential operators guarantee both the sparsity and continuity (smoothness) of the solutions [65]. Here, the solution is decomposed into two components (*i* = 1, 2) to simultaneously capture two distinct characteristics, narrowband and broadband features. Since the six regularization weights (*α_i_*, *β_i_*, *γ_i_*) significantly affect the reconstruction performance, they are carefully optimized through genetic algorithms (see Note S15). Finally, this minimization problem is solved via convex optimization using the CVX package in MATLAB [66].

To assess the performance of the spectral reconstruction using the proposed system, we conducted simulations similar to real‐world measurement conditions. To reflect experimental errors, we introduced 0.5% additive white Gaussian noise (AWGN) into the simulated data. We adopted fidelity (*F*) as the quantitative metric to evaluate the similarity between the original input spectrum *Φ*_0_ and the reconstructed spectrum *Φ*, defined as *F* (Φ_0_,Φ) = Φ_0_  · Φ/(|Φ_0_|_2_|Φ|_2_). Figure 4c shows reconstructed results for narrowband Gaussian spectra with a standard deviation of 50 cm^−1^, centered at various frequencies. These results represent the median performance derived from 200 independent trials with random noise. As a result, a high fidelity (*F* > 98%) was achieved over most of the frequency range, demonstrating highly precise reconstruction performance, while maintaining a reliable accuracy of *F* = 97.2% even near the spectral edge region at 2400 cm^−1^.

Furthermore, reconstruction was performed for broadband or complex‐shaped signals, as shown in Figure 4d. Although these represent challenging cases, we successfully reconstructed the overall spectral features based on the proposed two‐component (*Φ*_1_+*Φ*_2_) algorithm. While Figure 4d presents the best‐case reconstruction results, the high fidelity (> 98%) achieved for broadband and complex signals, alongside the successful narrowband reconstruction, strongly indicates that the proposed device platform has significant potential as a graphene‐based single‐chip spectrometer capable of analyzing complex real‐world optical signals beyond simple peak detection. Additional spectral reconstructions performed under higher AWGN levels (up to 4%), as well as with a spectrometer‐optimized metasurface using a relaxed graphene scattering rate, are detailed in Note S16.

## 3 Conclusion

In conclusion, we have demonstrated an active mid‐IR transmissive metasurface that simultaneously achieves high transmission modulation efficiency and a broad operational bandwidth based on a graphene‐gold hybrid architecture on a CaF_2_ substrate. The device achieves a transmission modulation efficiency of 73.3% with a 742 cm^−1^ operational bandwidth, whereas a different device geometry extends the bandwidth to 1949 cm^−1^ with an efficiency of 44%. Circuit model analysis confirms that the observed modulation originates from a parallel LC resonance formed by the capacitive gold grating and the inductive response of the gated graphene. Furthermore, we exploit the spectral diversity generated by Fermi‐level modulation to implement a single‐chip spectrometer, achieving high‐fidelity reconstruction of complex signals through a compressed sensing algorithm. While the present demonstration is focused on TM‐polarized illumination, the proposed LC resonance mechanism can be readily extended to a two‐dimensional crossed‐grating architecture to achieve polarization‐independent modulation (see Note S17). The modulation speed of the present device is a few tens of seconds (see Note S3), limited by the slow ion migration dynamics associated with ionic‐gel gating. We note that, although ionic‐gel gating was employed here as a proof‐of‐concept to induce large Fermi‐level shifts, our operating principle is not inherently bound to liquid electrolytes. Adopting solid‐state electrostatic gating schemes with high‐k dielectric can overcome the speed limitation of ionic mobility while preserving comparable high‐efficiency modulation and extending the operational frequency toward the long‐wave infrared (LWIR) regime (see Note S18). We anticipate that further investigation into the radiative coupling balance between the transmission and reflection channels will lead to even higher modulation efficiencies, broader operational bandwidths, and enable more sophisticated light manipulation, including independent control of transmission phase and amplitude. This compact and electrically tunable platform provides an efficient framework for next‐generation integrated mid‐IR applications, such as on‐chip spectroscopy and sensing systems.

## 4 Methods

### 4.1 Device Fabrication

The tunable transmission filters were fabricated on a 500 µm‐thick CaF_2_ substrate (CFc101005S2, double‐side polished, (111) direction, MTI Korea). Source and drain pads (Ti/Au (5 nm/50 nm)) were patterned using photolithography (MJB4 Mask Aligner, SUSS MicroTec) and deposited on the CaF_2_ substrate using a thermal evaporator (Pure Vacuum Technology). Patterns (Ti/Au (2 nm/10 nm)) located between the source and drain pads were also deposited through thermal evaporator also and electron beam lithography was followed. Electron beam lithography was carried out by a modified SEM system (Hitachi S‐4300) with an acceleration voltage of 30 keV. PMMA C2 electron beam resist was used for patterning, and Espacer 300Z was also used for preventing charge effects and achieve fine resolution. After the patterning, Ar milling process (MPS 3000FC, Ion Tech INC.) was followed to create a small gap by physical etching. Ar gas was used for etching and the remaining PMMA C2 layer acted as an etching mask. Monolayer graphene grown by chemical vapor deposition was directly wet‐transferred onto the pattern. Ionic gel was prepared from P(VDF‐HFP) (poly(vinylidene fluoride‐co‐hexafluoropropylene)), [BMIM][PF_6_], and acetone and spin‐coated on a graphene layer with a thickness of approximately 3 µm. The fabricated sample was bonded to PCB substrate using a wire bonder (7476D, Westbond Inc.), and an external voltage was applied to ionic gel through the bonding. Additional fabrication details are described in Note S1.

### 4.2 Measurement

FTIR was used to measure the transmission of tunable filter devices. A polarizer plate was installed, and the signal was measured while rotating it in 5° increments. The TE mode was defined as the lowest signal intensity and TM mode as the highest signal intensity. By adjusting the iris to be slightly smaller than the pattern size of 80 µm × 80 µm, only the signal from the entire pattern area was collected. External voltage applied to graphene through the ionic gel layer was carried out by KEITHLEY 2400.

### 4.3 Electromagnetic Simulations

Full‐wave electromagnetic simulations were performed with RETICOLO V9, an open‐source MATLAB‐based RCWA solver. A Fourier order of 425 was employed for the structure with *p* = 3 µm and *g* = 50 nm. The surface optical conductivity of graphene was calculated based on the random phase approximations. The complex refractive index of ionic gel ([BMIM][PF_6_]) was determined using mid‐IR spectroscopic ellipsometry (IR VASE II, J.A. Woollam). For the 10 nm‐thick thin‐film gold and CaF_2_ substrate, the refractive indices were extracted by fitting the measured transmission spectra to the Brendel–Bormann model. The refractive index of Ti was adopted from Rakić and also parameterized using the Brendel‐Bormann model. Detailed procedures for determining these refractive indices are provided in Note S4. In the equivalent circuit model, the layer of the periodic gold slit array incorporating graphene was represented by an effective surface admittance derived from the circuit components. Subsequently, the Transfer Matrix Method (TMM) was employed to calculate the transmission spectra of the structure. Given that the thickness of the 500 µm‐thick CaF_2_ substrate significantly exceeds the coherence length of the incident light, Fabry–Pérot resonances are effectively suppressed. Therefore, the CaF_2_ substrate was treated as an incoherent layer in the TMM calculations. Detailed derivations and parameters for the equivalent circuit model are provided in Note S6.

### 4.4 Spectral Reconstruction Algorithm

The spectral reconstruction process was performed based on the compressive sensing theory detailed in the main text (Equations (3), (4), (5)). The convex optimization problem was solved using the CVX package in MATLAB. The input spectrum was decomposed into two components, and the regularization weights (*α_i_*, *β_i_*, *γ_i_*) were optimized via genetic algorithms.

## Author Contributions

H.H., Junhyung Kim, and M.S.J. conceived the ideas. H.H. fabricated the metasurfaces and measured the experimental data. Jinseok Kong conducted the electromagnetic simulations, performed the equivalent circuit model analysis, and demonstrated the spectrometer application. Jiwon Kang measured the refractive index of the ionic gel. H.H., Jinseok Kong, and M.S.J. wrote the manuscript. M.S.J. supervised the project.

## Conflicts of Interest

The authors declare no conflicts of interest.

## Supporting information




**Supporting File**: advs77550‐sup‐0001‐SuppMat.pdf.

## Data Availability

The data that support the findings of this study are available from the corresponding author upon reasonable request.
