# Supplementary material for: Electrically Tunable Graphene‐Metal Hybrid Metasurfaces for High‐Efficiency, Broadband Mid‐Infrared Transmission Modulation
Source: Adv Sci (Weinh). 2026 Sep 3:e77550. Online ahead of print. doi: 10.1002/advs.77550 (PMC13542519; doi:10.1002/advs.77550)
Supplement: Supplementary file 1 [file ADVS-9999-e77550-s001.pdf]

Supplementary Information for

Electrically tunable graphene-metal hybrid metasurfaces for high-efficiency, broadband mid-infrared transmission modulation

Heonhak Ha[1,†], Jinseok Kong[1,†], Junhyung Kim[1], Jiwon Kang[1], and Min Seok Jang[1,*]

[*] jang.minseok@kaist.ac.kr

[†] Equally contributed authors

[1] School of Electrical Engineering, Korea Advanced Institute of Science and Technology, Daejeon 34141, Republic of Korea

This file includes:

Supplementary Notes 1 to 19

1. Device fabrication steps
2. Device statistics
3. Characterization of ionic-gel-gated metasurfaces
4. Material refractive index fitting
5. Transmission characteristics under TE-polarized illumination
6. Incoherent calculation using the transfer matrix method (TMM) and RCWA
7. Fitting procedure for the graphene Fermi level
8. Full-wave RCWA simulations at normal incidence
9. Dependence of transmission on conical incidence angles
10. Equivalent circuit model components
11. Approximations in the circuit model
12. Circuit model analysis for an ideal lossless system
13. Analysis of graphene plasmon resonance contributions
14. Dependence of transmission modulation on geometric parameters
15. Optimization of regularization weights via genetic algorithms
16. Additional numerical analysis of the computational spectrometer
17. Polarization-independent 2D grating metasurface design
18. Extension toward LWIR operation and fast solid-state gating using BaF_2 and HfO_2
19. Comparison with previously reported electrically tunable transmissive mid-infrared modulators

Supplementary Note 1. Device fabrication steps

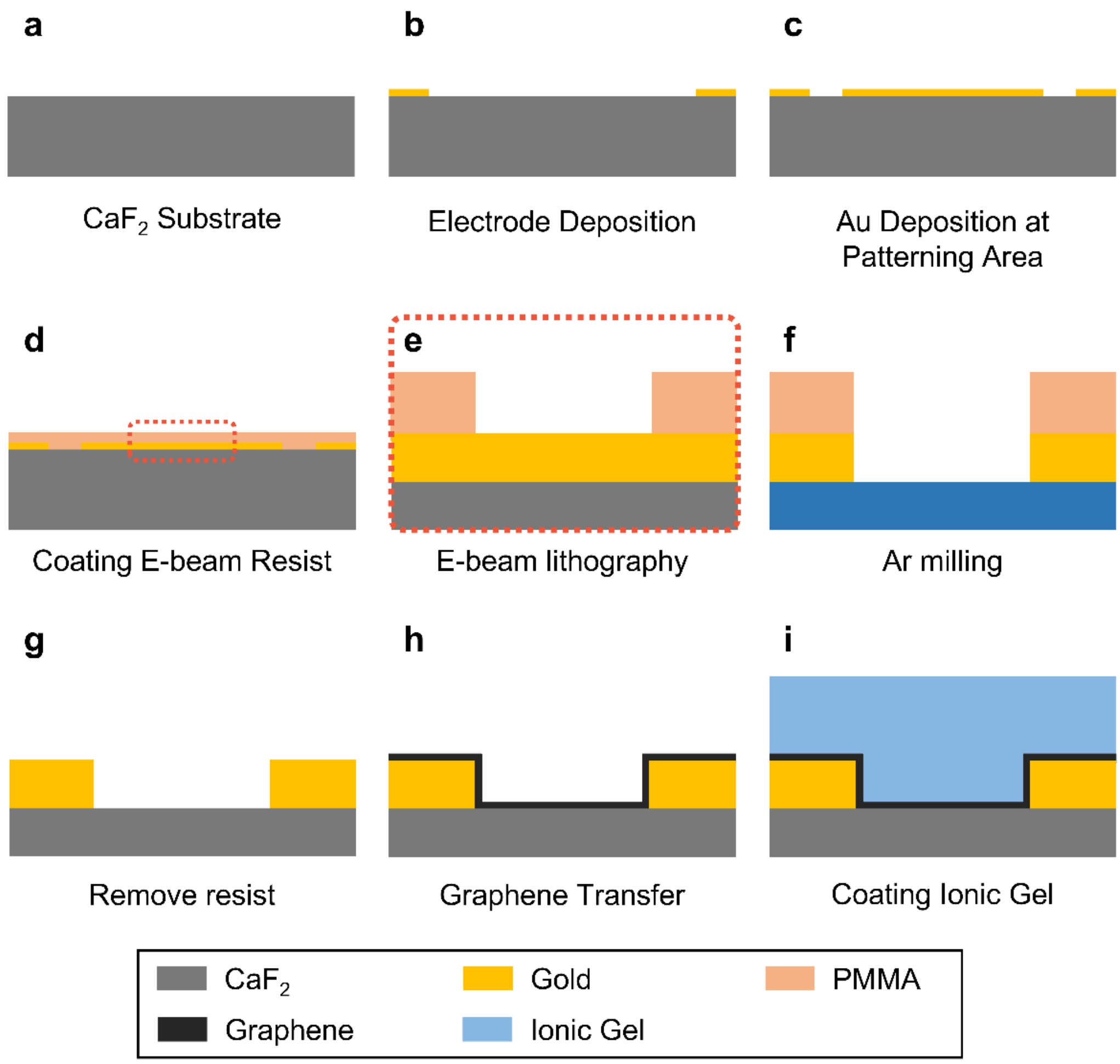

Figure S1. Steps for fabricating the active transmission filter. **a** A 500-µm-thick, double-side-polished CaF_2 substrate is prepared. **b** A 50-nm-thick gold source and drain with a 5-nm Ti adhesion layer are deposited on the CaF_2 substrate using thermal evaporator. **c** 10-nm-thick gold pads with a 2-nm Ti adhesion layer for the grating pattern are deposited between the source and drain using a thermal evaporator. **d** PMMA C2 and Espacer 300Z are spin-coated onto the device. **e** The desired pattern is created by electron-beam lithography. **f** The metal grating structure is formed through an Ar milling process. **g** Residual PMMA C2 is removed through acetone and isopropyl alcohol (IPA). **h** CVD-grown graphene is transferred onto the fabricated structure. **i** [BMIM][PF_6] ionic gel layer is spin-coated onto the graphene layer.

Supplementary Note 2. Device statistics

To evaluate the reproducibility of the proposed metasurface, we analyzed six devices with identical geometrical parameters (p = 3 μm and g = 50 nm). The transmission peak frequency, peak modulation efficiency, modulation peak frequency, and modulation bandwidth were evaluated for each device and are summarized in Table S1. The six devices exhibit an average transmission peak frequency of 1021 ± 47 cm^{-1}, peak modulation efficiency of 69.7 ± 5.7%, a modulation peak frequency of 1274 ± 20 cm^{-1}, and a modulation bandwidth of 732 ± 36 cm^{-1} (mean ± standard deviation). These results demonstrate the reproducibility of the transmission modulation characteristics across multiple devices and samples with the same geometry.

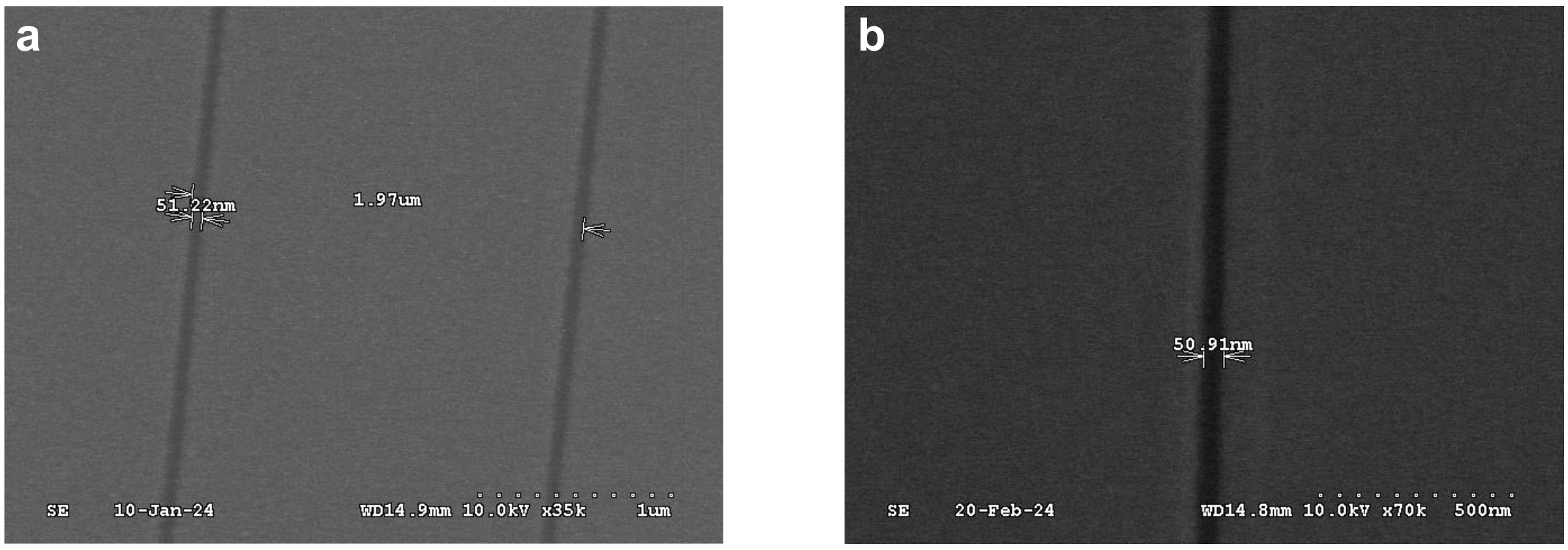

Figure S2. Representative SEM images of the fabricated metasurface with a nominal gap size of 50 nm. a, b SEM images of the different fabricated structures, showing measured gap widths close to their nominal gap size of 50 nm.

We also performed a statistical analysis of the fabricated gap widths using SEM images shown in Figure S2. Since the 50 nm gap represents the minimum feature size of our device, we focused our statistical analysis on structures with a nominal gap size of 50 nm to evaluate the fabrication accuracy of the most demanding feature. Eight gaps were quantitatively measured from SEM images, yielding an average gap width of 53.2 ± 2.5 nm (mean ± standard deviation, n = 8). The measured gap dimensions are reasonably consistent with the nominal design value, indicating that good dimensional control of the fabricated nanoscale gaps.

Regarding the graphene transfer quality, the polymer-assisted wet-transfer process can introduce local wrinkles, residues, or defects in the transferred graphene, which may contribute to device-to-device variation in graphene conductivity and optical modulation characteristics. Although a separate statistical characterization of the graphene transfer quality was not performed, the six devices analyzed in this study were fabricated across five different samples involving separate graphene transfer process. The relatively small variations in the modulation peak frequency and bandwidth suggest that

the overall modulation behavior is reproducibly maintained across different samples, despite possible variations associated with the graphene transfer process.

Device (p = 3 μm and g = 50 nm)	Peak frequency (cm^{-1})	Modulation peak frequency (cm^{-1})	η_T (%)	Bandwidth (FWHM)
Device 1 (Representative)	998	1265	73.3	724
Device 2	1052	1291	75.5	753
Device 3	1100	1252	60	692
Device 4	980	1282	66.5	787
Device 5	980	1256	71	696
Device 6	1015	1301	72.3	740
Mean ± Standard deviation	1021 ± 47	1274 ± 20	69.7 ± 5.7	732 ± 36

Table S1. Device-to-device reproducibility. Transmission modulation characteristics of six devices with identical geometrical parameters (p = 3 μm and g = 50 nm) fabricated across five different samples.

Supplementary Note 3. Characterization of ionic-gel-gated metasurfaces

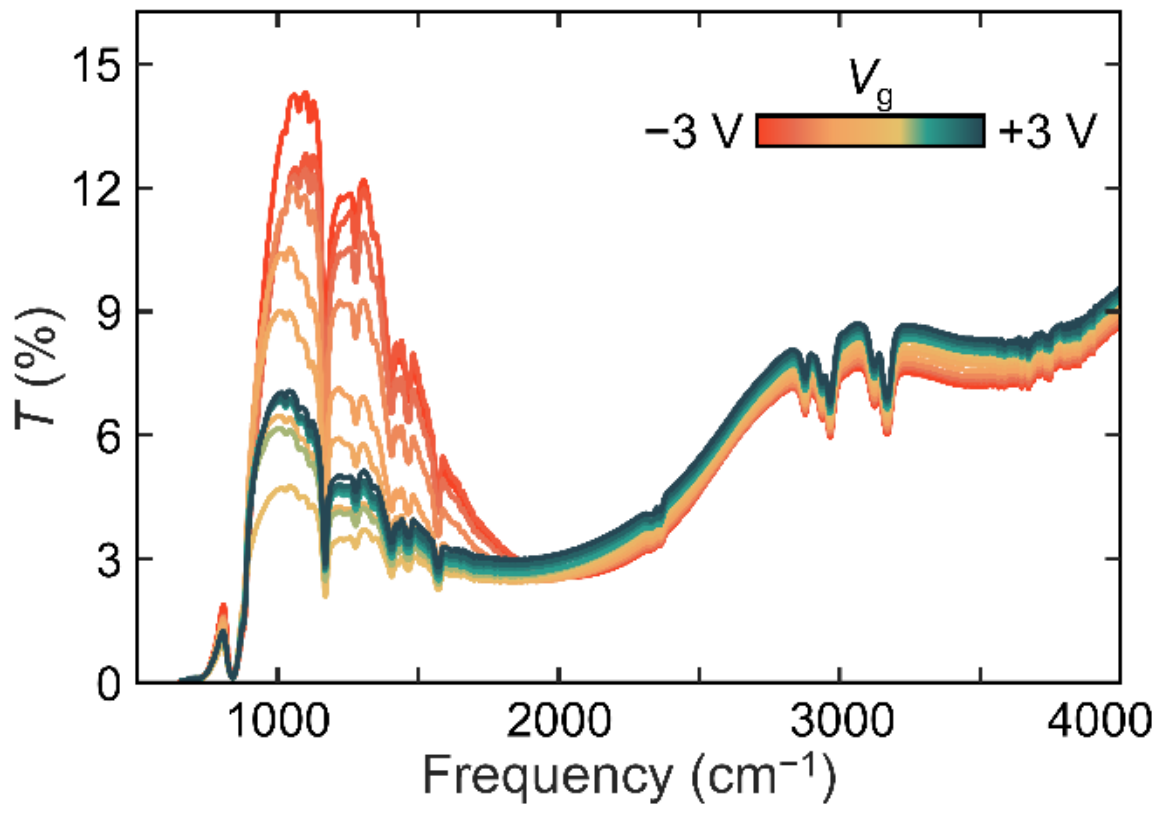

Figure S3. Transmission spectra at different applied gate voltages. Transmission spectra of a device (p = 2 μm and g = 50 nm) measured under applied voltages from −3 V to +3 V across the ionic gel layer.

Figure S3 shows the transmittance measured for a device with a period of 2 μm and a gap of 50 nm while the voltage applied to the ionic gel was varied from −3 V to +3 V in steps of 0.5 V. Since the ionic gel consists of distinct cations and anions, its response to negative and positive bias is inherently asymmetric depending on the ionic species [1]. The ionic gel layer used in this study is based on [BMIM][PF_6], in which the influence of the PF_6^- anion is more pronounced than that of the $BMIM^+$ cation. Accordingly, under negative bias, PF_6^- ions accumulate on the graphene surface and effectively modulate the Fermi level, resulting in a significant change in transmittance. In contrast, under positive bias, the contribution of PF_6^- is minimal, leading to negligible modulation of the transmittance even at an applied voltage of +3 V [2].

To evaluate the characteristics of the ionic-gel-gated device, we investigated the modulation response time, optical hysteresis under forward/reverse voltage sweep, stability under a fixed gate bias, repeatability over multiple voltage cycles, and long-term storage stability.

3.1 Modulation response time

The modulation speed of the present device is primarily limited by the relatively slow dynamics of the ionic gel, including ion migration, charge trapping, and relaxation processes associated with electric double-layer formation. To quantitatively evaluate the switching speed, we performed additional time-resolved measurements using the free-space optical setup shown in Figures S4a, b. Because the free-space measurement was performed under ambient conditions without a purged sample chamber, a limited voltage range (−2 V to −1 V) was employed to minimize the degradation of the ionic gel caused by moisture during repeated switching. A quantum cascade laser operating at ω = 1059 cm^{-1} was used as the light source, and the transmitted optical signal was monitored using an MCT detector

with a lock-in amplifier. The gate voltage was initially swept from 0 V to −2 V at a rate of 0.05 V/s and subsequently switched repeatedly between −2 V and −1 V with a holding time of 180 s, while the detector signal was recorded every 0.2 s. The response time was extracted using the conventional 10%–90% criterion. As shown in Figure S4c, the average measured rise and fall response times are approximately 50.9 s and 11.7 s, corresponding to a modulation rate of approximately 0.02 Hz.

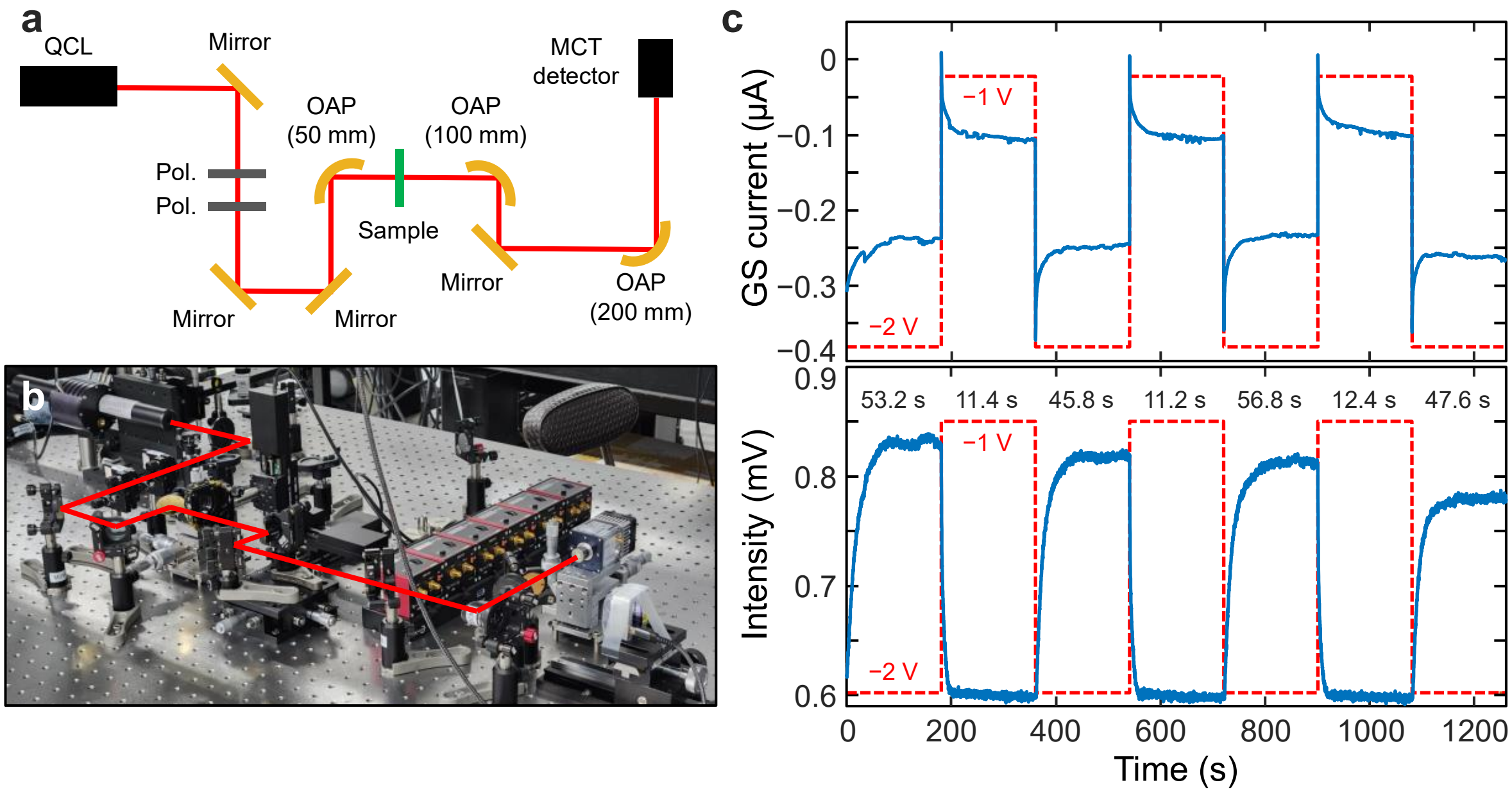

Figure S4. Modulation speed of the ionic gel gating. a Schematic of the free-space optical measurement setup. **b** Photographic image of the experimental setup. **c** Measured gate current and optical response during repeated voltage switching between −2 V and −1 V.

3.2 Optical hysteresis under forward/backward voltage sweeps

We measured both the electrical characteristics and optical transmission spectra during forward and reverse gate-voltage sweeps for a graphene metasurface with p = 3 μm and g = 50 nm. The gate voltage was swept in a butterfly sequence (0 V → −3 V → +3 V → + 0 V) with a step size of 0.05 V and a dwell time of 1 s at each voltage step. The optical transmission spectra were measured at V_g = −3 V, −2 V, −1 V, 0 V, +0.25 V, +0.5V, +0.75 V, and +1 V during both the forward and reverse sweeps, with each FTIR measurement requiring approximately 90 ~ 120 s. As shown in Figure S5a, the electrical measurements exhibit a hysteresis of approximately 0.5 V in the charge neutrality point (CNP), which is likely due to the slow redistribution of ions within the ionic gel. The optical transmission spectra shown in Figures S5b and S5c reproduce similar modulation characteristics in both sweep directions, while the CNP shift is consistently reflected in the optical response. This behavior is consistent with the typical hysteresis observed in electrolyte-gated graphene devices.

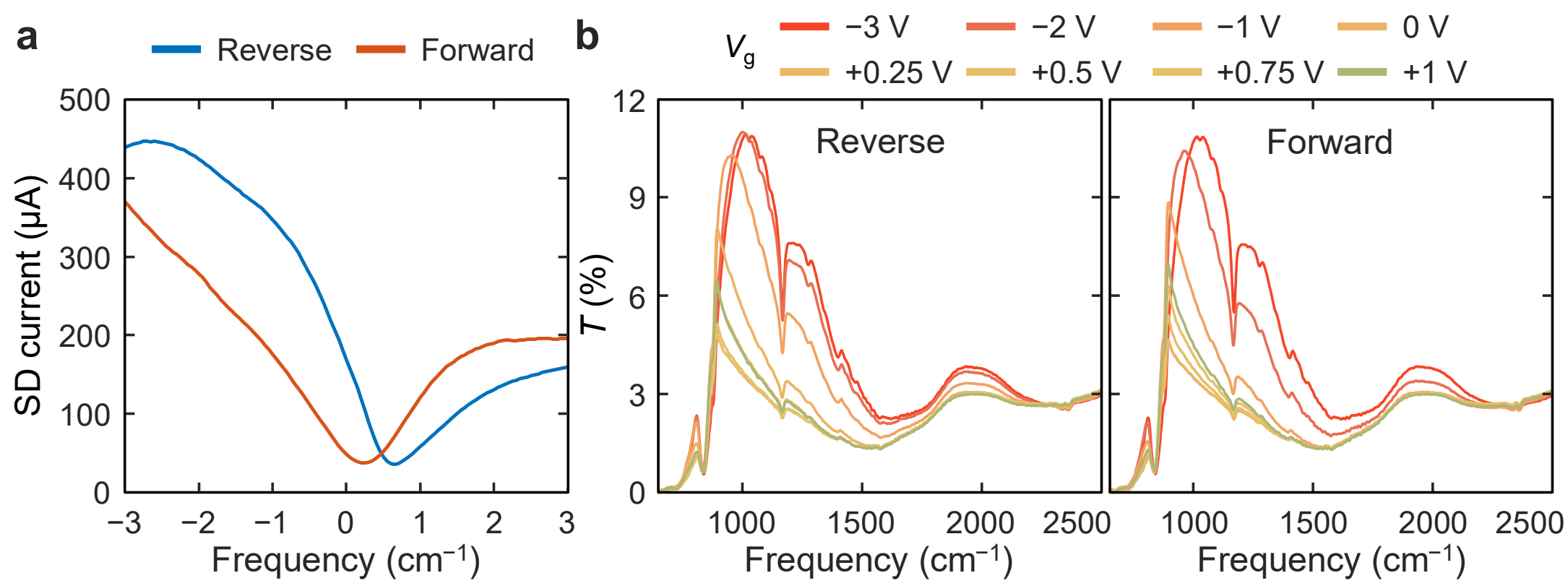

Figure S5. Optical hysteresis measurements of the graphene metasurface (*p* = 3 μm and *g* = 50 nm). a Electrical characteristics measured during forward and reverse gate-voltage sweeps. **b** Measured transmission spectra obtained during reverse (left) and forward (right) gate-voltage sweeps.

3.3 Short-term Stability under fixed bias

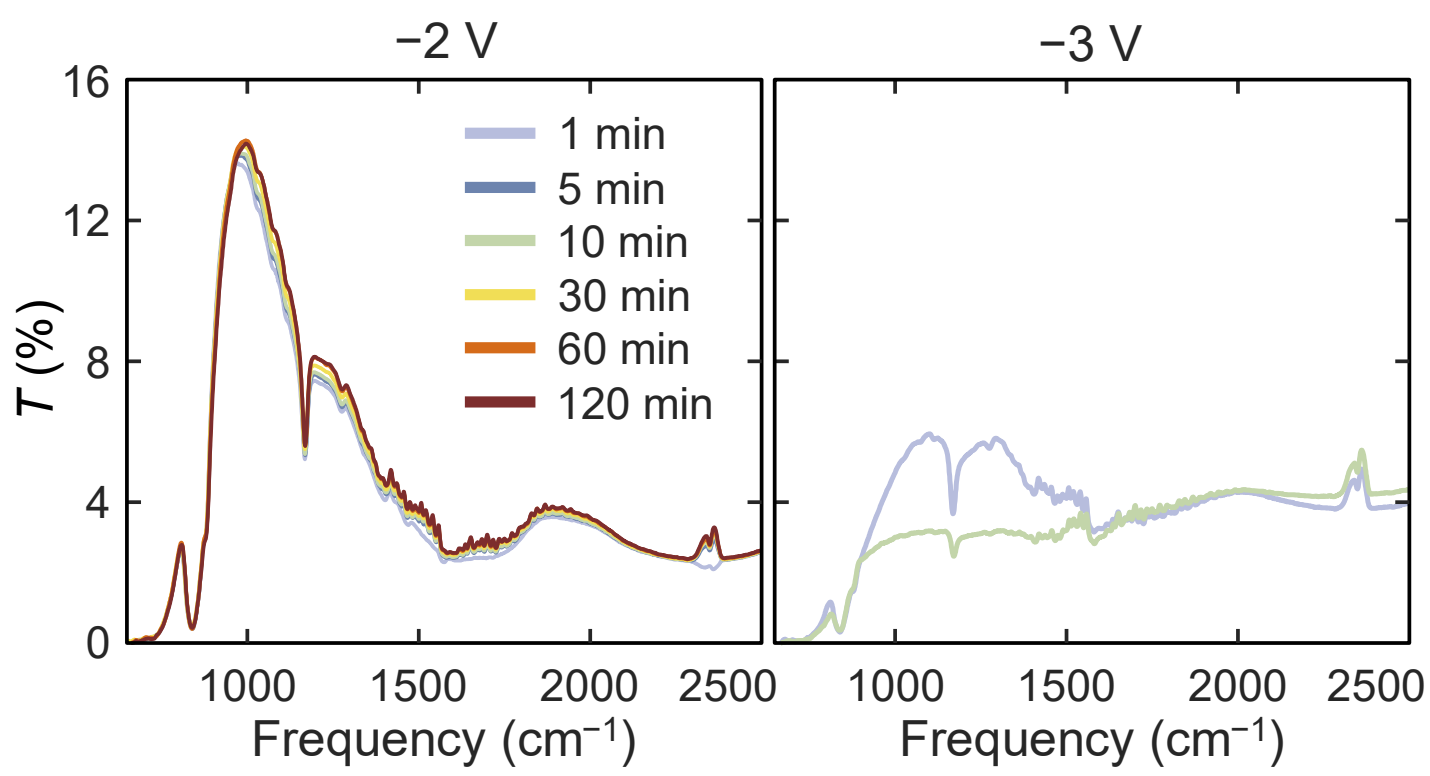

Figure S6. Stability of the optical modulation under a fixed gate bias for the graphene metasurface (*p* = 3 μm and *g* = 50 nm). Transmission measured after 1, 5, 10, 30, 60, and 120 min under a constant gate bias of $V_g = -2$ V (left) and after 1 and 10 min under a constant gate bias of $V_g = -3$ V (right).

We further investigated the stability of the optical modulation under a constant gate bias. Because the transmission spectra measured at $V_g = -2$ V are already comparable to those obtained at −3 V, we first evaluated the fixed-bias stability at −2 V using a separate device to avoid the influence of degradation induced by prolonged operation at $V_g = -3$ V. As shown in Figure S6 (left panel), the device maintained stable optical modulation for at least 2 hours without noticeable degradation, demonstrating stable continuous operation within the practical operating conditions.

To further examine the stability limit of the ionic-gel-gated device, we additionally performed the same measurement under a continuous biasing at $V_g = -3$ V. As shown in Figure S6 (right panel), the transmission gradually decreased with time, despite the graphene channel remaining electrically

conductive. Previous studies have reported that prolonged operation near the electrochemical stability limit of BMIM-based ionic liquids, particularly in the presence of residual water, can induce irreversible electrochemical processes that reduce the effectiveness of electrostatic gating [3, 4]. Consistent with these reports, we observed a gradual degradation of the optical modulation under continuous −3 V bias. It should be noted that no noticeable degradation was observed during the relatively short measurement period used for the characterization throughout this work. In contrast, a gradual degradation became apparent only under prolonged continuous biasing at a constant gate voltage for more than several minutes. Therefore, the reported device characteristics are expected to be only minimally affected by this long-term bias-induced degradation.

3.4 Long-term stability

In addition, we evaluated the long-term storage stability of the device. Figures S7a and S7b (left panels) show the initial transmission spectra and modulation efficiency, respectively. After maintaining the device in a desiccator at room temperature and low humidity for over 3 months, the measurements were repeated (Figures S7a and S7b, right panels). While the absolute transmittance exhibited a minor decrease, the modulation efficiency and overall switching performance remained largely intact, confirming the good long-term durability of the device.

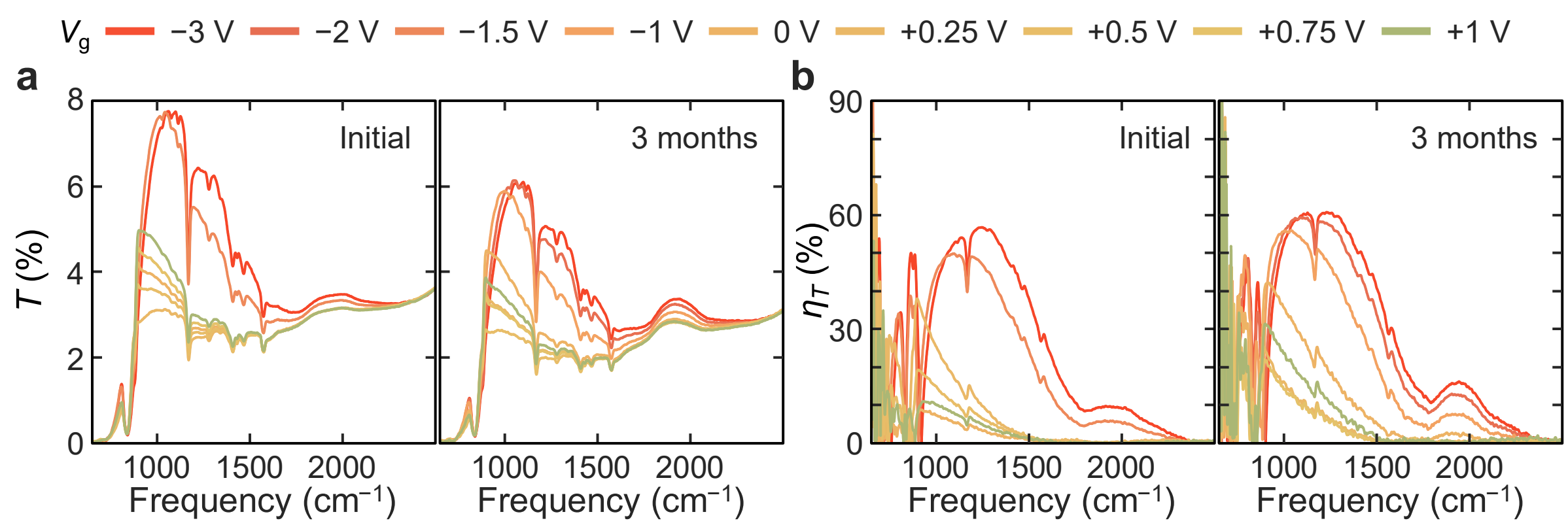

Figure S7. Long-term storage stability of the graphene metasurface (*p* = 3 μm and *g* = 50 nm). a Measured transmission spectra of the device before storage (left) and after storage in a desiccator for over 3 months (right) at room temperature under low-humidity conditions. **b** Corresponding transmission modulation efficiency before (left) and after (right) storage.

3.5 Repeatability over multiple voltage cycles

To evaluate the repeatability of the device, the gate voltage was repeatedly swept in a butterfly sequence (0 V → −3 V → +3 V → + 0 V) for ten consecutive cycles. The voltage was swept with a step size of 0.05 V and a dwell time of 1 s at each voltage step. The optical transmission spectra were

measured at $V_g = -3$ V, −2 V, −1 V, 0 V, +0.25 V, +0.5V, +0.75 V, and +1 V during each voltage sweep, with each FTIR measurement requiring approximately 90 ~ 120 s. For clarity, Figure S8 compares only the transmission spectra measured at $V_g = -3$ V for each cycle, together with the corresponding modulation efficiency. As shown in Figure S8, both the transmission spectra and the corresponding modulation efficiency remain highly reproducible throughout the repeated measurements. Although slight variations are observed after repeated operation, the overall spectral characteristics and modulation efficiency are well preserved, demonstrating good repeatability under cyclic electrical operation.

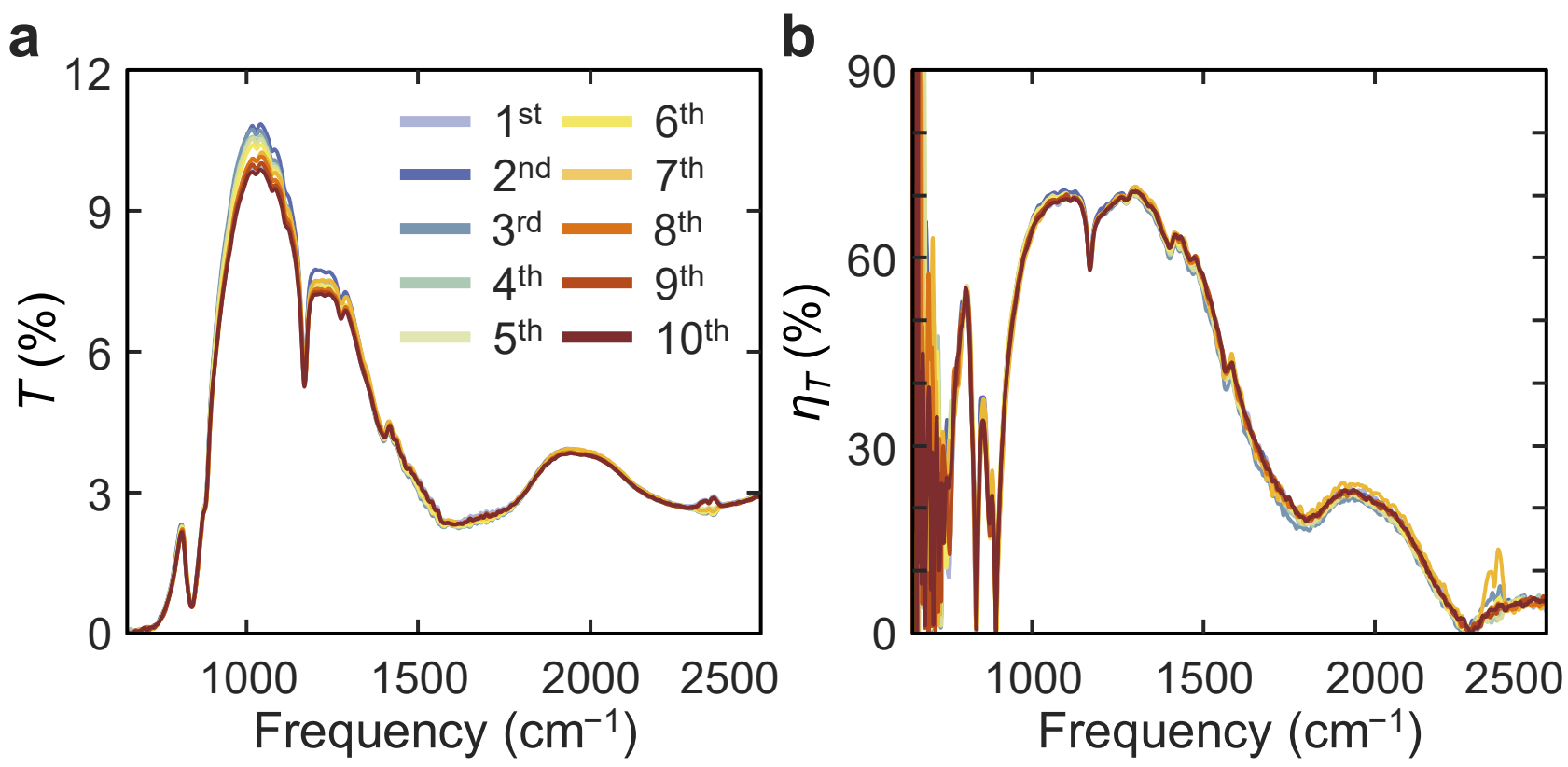

Figure S8. Repeatability of the graphene metasurface (p = 3 μm and g = 50 nm) under repeated gate-voltage sweeps. a Measured transmission spectra and **b** corresponding transmission modulation efficiency for ten consecutive voltage sweep cycles.

Supplementary Note 4. Material refractive index fitting

4.1 Refractive index fitting of CaF2 and thin Au using the Brendel-Bormann model

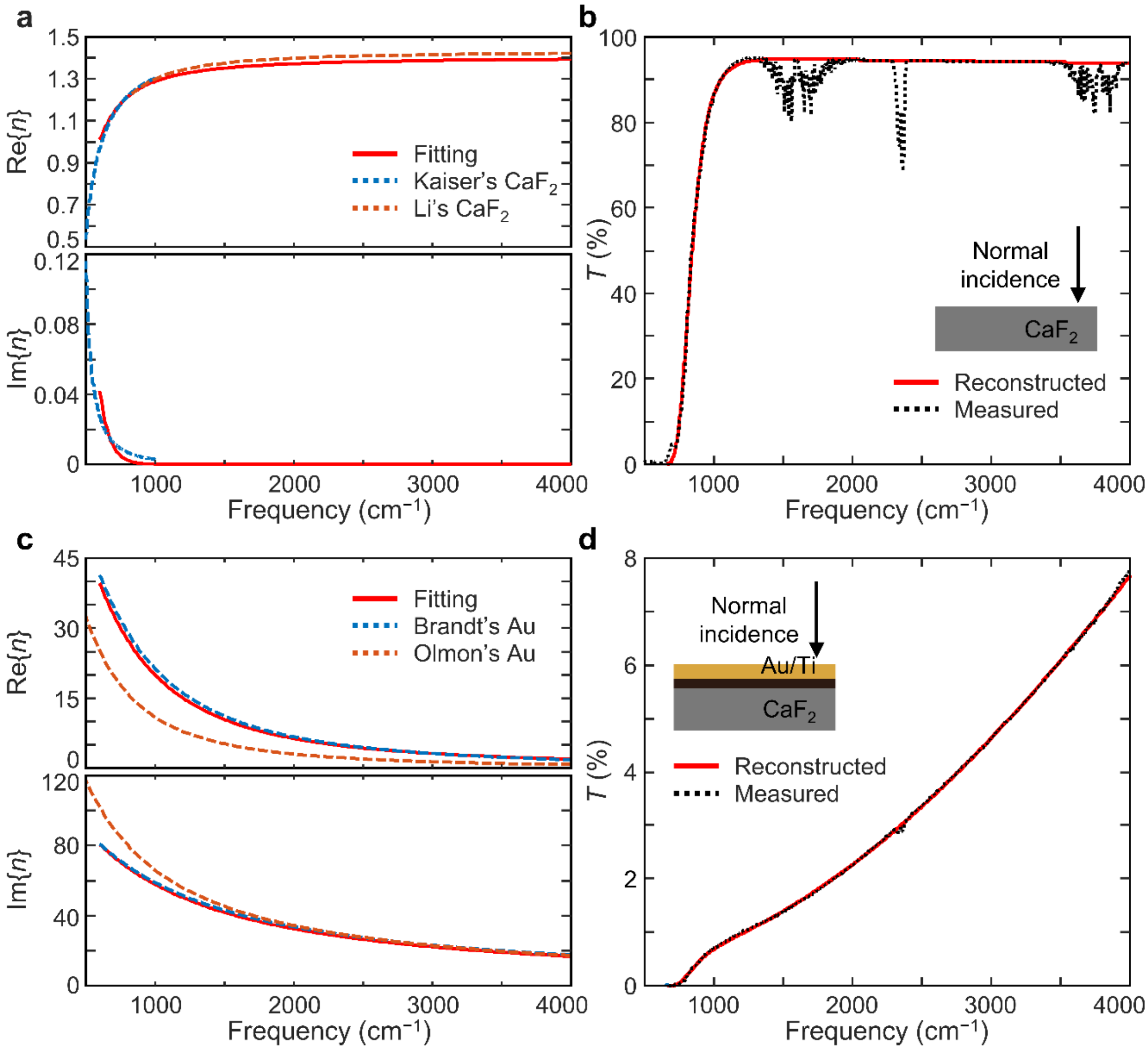

Figure S9. Results of fitting the refractive index of CaF_2 and thin Au. a Fitted real (upper panel) and imaginary (lower panel) parts of the CaF_2 refractive index as a function of frequency (red solid lines). Reference values from W. Kaiser [5] (blue dashed line) and H. H. Li [6] (orange dashed line) are also provided for validation. **b** Comparison of the theoretically calculated (red solid line) and experimentally measured (black dashed line) normal-incidence transmittance for the bare CaF_2 substrate. **c** Fitted real (upper panel) and imaginary (lower panel) parts of the thin Au refractive index as a function of frequency (red solid lines). Reference values of 9-nm-thick Au (blue dashed line) from T. Brandt [7] and evaporated Au (orange dashed line) from R. L. Olmon [8] are also provided for validation. **d** Comparison of the theoretically calculated (red solid line) and experimentally measured (black dashed line) normal-incidence transmittance for the thin Au film on a CaF_2 substrate with a 2-nm Ti adhesion layer.

The refractive indices of calcium fluoride (CaF_2) and thermally evaporated thin gold (Au) were extracted using transmission-based fitting. The measurements were performed in the FTIR sample compartment under unpolarized, normal incidence conditions over the operating frequency range of 650–4000 cm^{-1}. The CaF_2 substrate used in the device has a thickness of 500 μm. Transmittance through the bare substrate was analyzed using the transfer matrix method (TMM) to extract its complex refractive index. Due to the surface-roughness and large optical thickness of the substrate, the CaF_2 layer was treated as an incoherent layer while using the TMM calculation to suppress interference fringes which is obviously not observed in the measured spectra. The detailed process of calculating the transmission spectra using TMM is provided in Supplementary Note 5. The Brendel-Bormann model [9] was adopted for dispersion fitting, as described in the following Equations S1–S4:

$$\varepsilon(\omega) = \varepsilon_\infty + \sum_{k=1}^{m} \chi_k(\omega), \tag{S1}$$

$$\chi_k(\omega) = \frac{i\sqrt{\pi}\omega_{pk}^2}{2\sqrt{2}a_k(\omega)\sigma_k}\left[W\left(\frac{a_k(\omega) - \omega_{0k}}{\sqrt{2}\sigma_k}\right) + W\left(\frac{a_k(\omega) + \omega_{0k}}{\sqrt{2}\sigma_k}\right)\right], \tag{S2}$$

$$W(x) = e^{-x^2}\left(1 + \frac{2i}{\sqrt{\pi}}\int_0^x e^{t^2} dt\right), \tag{S3}$$

$$a_k(\omega) = \sqrt{\omega^2 + i\omega\tau_k}. \tag{S4}$$

Figure S9a shows the real and imaginary parts of the fitted refractive index of CaF_2, along with reference data from W. Kaiser [5] and H. H. Li [6] for comparison. The fitted result lies in close agreement with literature values over the entire spectral range. Figure S9b displays the measured and reconstructed transmittance spectra, confirming the model's accuracy.

Following the characterization of CaF_2, an additional sample was prepared by depositing a 2-nm Ti adhesion layer and a 10-nm Au film onto the fitted CaF_2 substrate. The transmittance spectrum of this multilayer structure was measured and used to extract the optical constants of the thin Au film. In the simulation, the optical properties of the Ti layer were fixed using the value reported by A. D. Rakić [9], and only the parameters of the Au layer were fitted using the Brendel-Bormann model for metal as shown in Equation S5:

$$\varepsilon(\omega) = 1 - \frac{\Omega_p^2}{\omega(\omega - i\Gamma_0)} + \sum_{k=1}^{m} \chi_k(\omega). \tag{S5}$$

Figure S9c presents the extracted complex refractive index of the 10-nm Au layer, alongside literature data from T. Brandt [7] and R. L. Olmon [8]. The fitted Au data show a spectral trend consistent with those of evaporated films. Figure S9d compares the measured and reconstructed transmittance, validating the accuracy of the extracted model over the operating spectral range.

4.2 Refractive index characterization of ionic gel using an ellipsometer

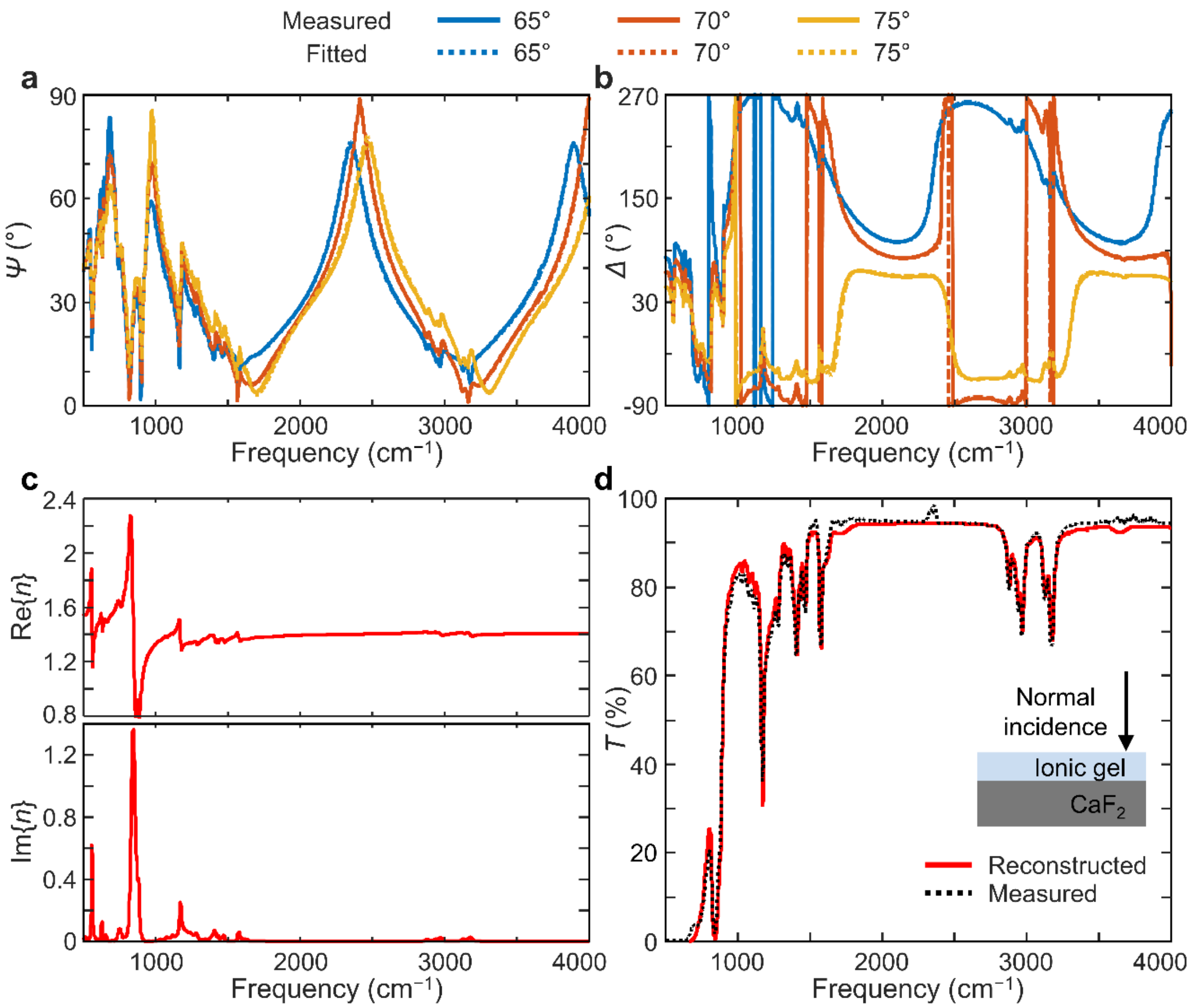

Figure S10. Results of fitting the refractive index of ionic gel. Measured (solid lines) and fitted (dashed lines) data for **a** *Ψ* and **b** *Δ* data at incidence angles of 65° (blue), 70° (orange), and 75° (yellow). **c** Extracted real (upper panel) and imaginary (lower panel) parts of the [BMIM][PF_6] refractive index as a function of frequency. **d** Comparison of the theoretically calculated (red solid line) and experimentally measured (black dashed line) normal-incidence transmittance for the spin-coated ionic gel on a CaF_2 substrate.

The tunable transmissive metasurface employed in this study utilizes [BMIM][PF_6] ionic gel as the gating medium. However, due to its complex molecular structure and strong vibrational absorption in the mid-IR regime, the material exhibits numerous absorption peaks and associated oscillators. As a result, fitting a dispersion model to reflectance or transmittance alone is highly challenging and can lead to inaccurate or non-unique solutions.

To circumvent this limitation, the complex refractive index of the ionic gel was directly measured using spectroscopic ellipsometry. This technique enables pointwise extraction of optical constants across the spectrum without assuming a specific model, and additionally provides access to film thickness. Measurements were performed using a J. A. Woollam IR-VASE Mark II ellipsometer, covering the wavelength range from 1.7 μm to 30 μm. The measured *Ψ* and *Δ*, along with the extracted

refractive index profile, are shown in Figure S10a–c. The film thickness was simultaneously determined to be 2.979 μm.

To independently verify the reliability of the ellipsometry results, a cross-validation using FTIR transmission measurements was performed. A sample was prepared by spin-coating [BMIM][PF_6] onto a fitted 500 μm-thick CaF_2 substrate. The transmittance spectrum of this structure was measured under unpolarized, normal incidence using the FTIR compartment, and reconstructed using TMM. As shown in Figure S10d, the reconstructed and measured spectra exhibit good agreement, confirming the validity of the extracted optical constants for simulation purposes.

4.3 Optical properties of graphene

For the optical simulations, the graphene monolayer is analytically treated as two-dimensional boundary of zero thickness. Its macroscopic optical response is characterized by the complex surface conductivity σ_{Gr}, derived from the local random phase approximation governing the Kubo formula as follows [10, 11]:

$$\sigma_{\mathrm{Gr}}(\omega, E_{\mathrm{F}}) = \frac{i2e^2 k_B T}{\pi\hbar^2(\omega + i\Gamma)} \log\left[2\cosh\left(\frac{E_{\mathrm{F}}}{2k_{\mathrm{B}}T}\right)\right] + \frac{ie^2(\omega + i\Gamma)}{\pi} \times \int_0^\infty dE\, \frac{f_d(-E) - f_d(E)}{[\hbar(\omega + i\Gamma)]^2 - (2E)^2}. \tag{S6}$$

Here, $f_d(E) = [1 + \exp((E - E_{\mathrm{F}})/k_{\mathrm{B}}T)]^{-1}$ and $\Gamma = ev_{\mathrm{F}}^2/(\mu E_{\mathrm{F}})$, where $v_{\mathrm{F}} \approx 10^6\ m/s$ represents the Fermi velocity and μ is the carrier mobility of the graphene layer. The system temperature T is fixed at a room temperature of 298.15 K.

For full-wave Rigorous Coupled-Wave Analysis (RCWA) simulations, the 2D complex surface conductivity σ_{Gr} was converted into the equivalent 3D complex permittivity using the relation $\varepsilon_{\mathrm{Gr}} = 1 + \frac{i\sigma_{\mathrm{Gr}}}{\omega\varepsilon_0 t_{\mathrm{GR}}}$, with an effective graphene thickness of t_{Gr} = 0.34 nm.

Supplementary Note 5. Transmission characteristics under TE-polarized illumination

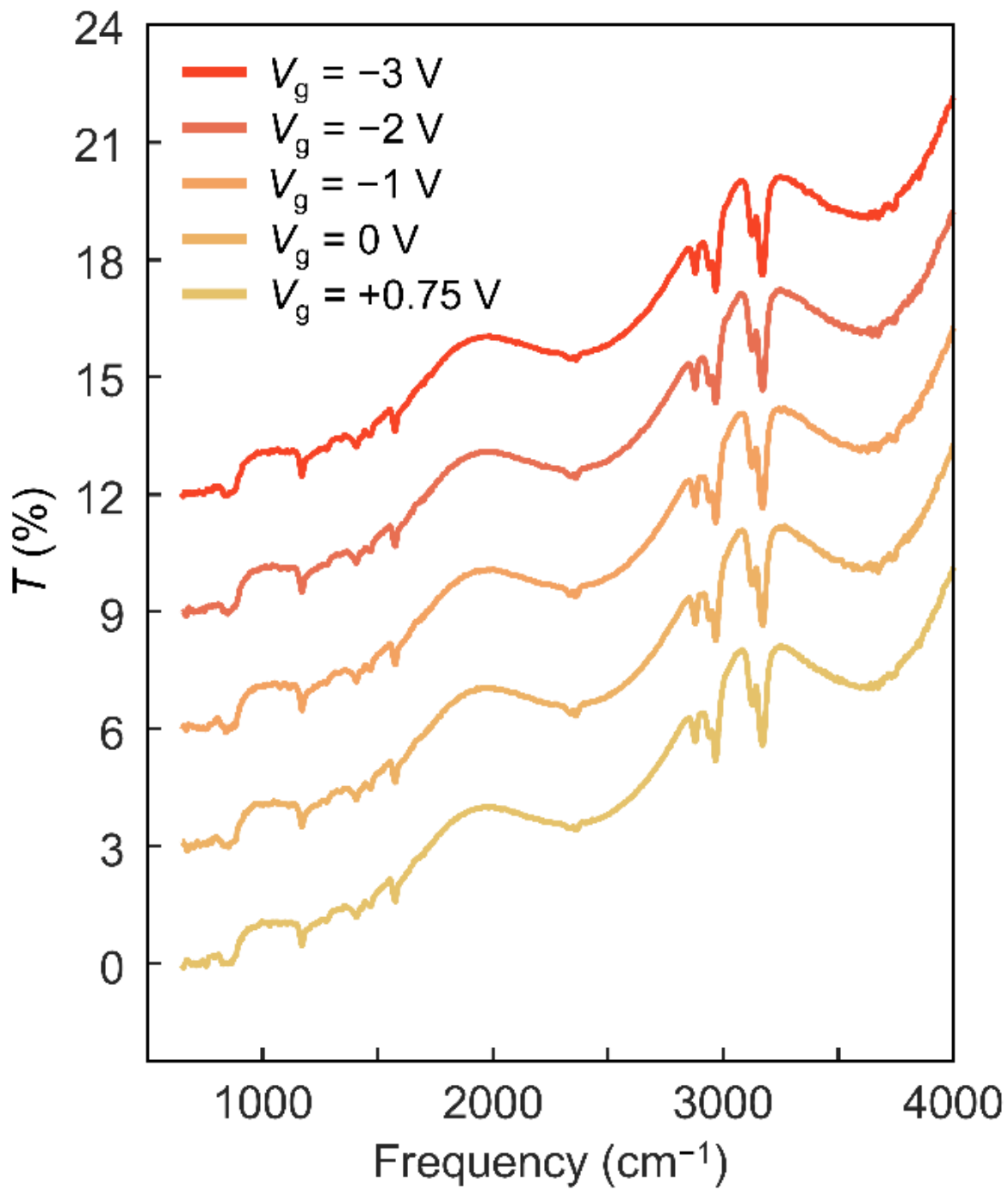

Figure S11. Measured transmission spectra for TE-polarized light. The gate voltage is swept from −3 V to +0.75 V. The spectra are vertically offset in steps of 3% for clarity. Unlike the transmission spectra for TM-polarized light shown in Figure 2a, only marginal changes occur with varying gate voltage.

Supplementary Note 6. Incoherent calculation using the transfer matrix method (TMM) and RCWA

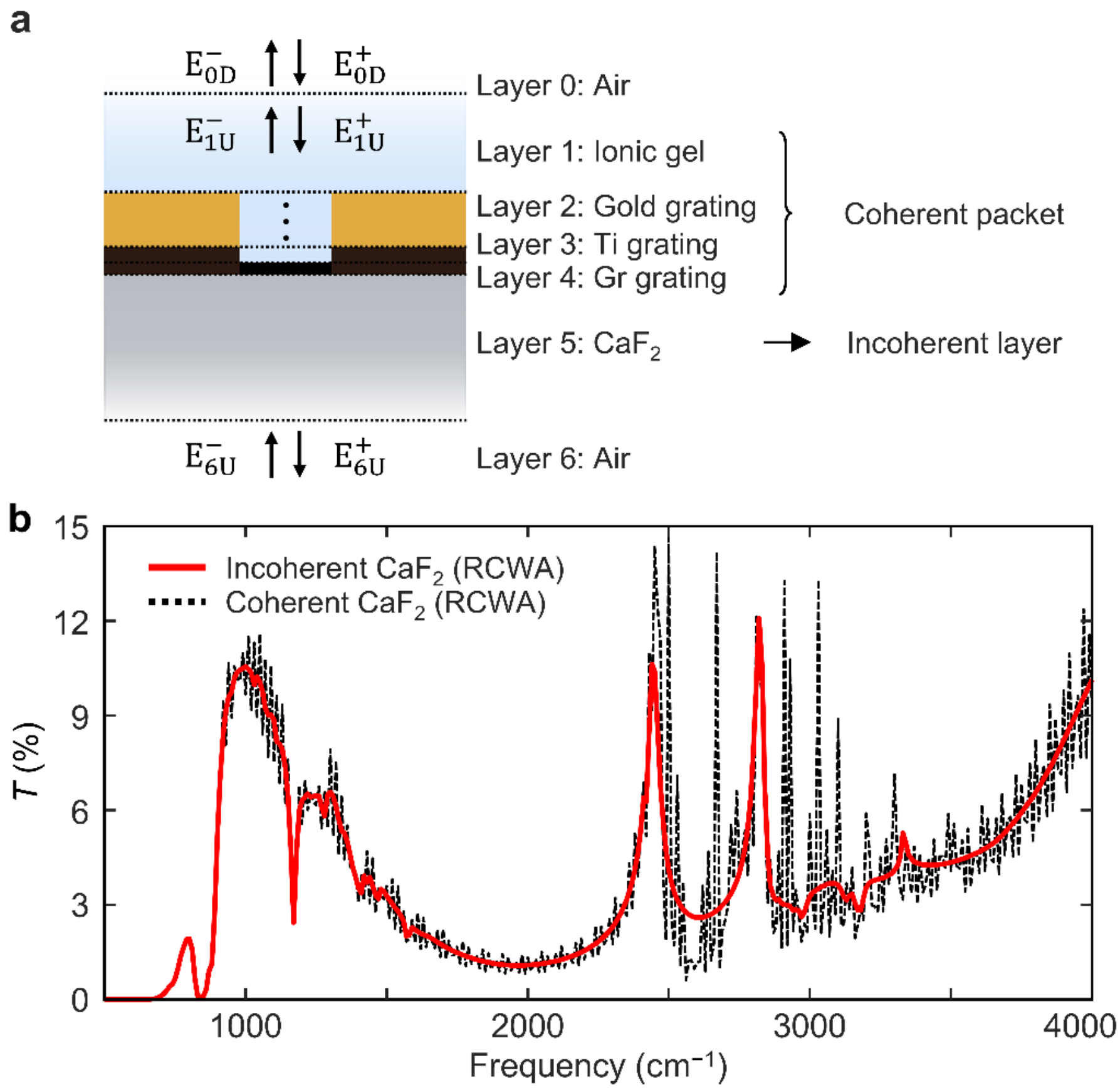

Figure S12. Incoherent transmission calculation. a Schematic illustration of the multilayer device structure divided into a coherent packet (Layers 1–4) and an incoherent layer (CaF_2 substrate). **b** Comparison of the calculated transmission spectra using the proposed incoherent calculation (red solid line) and the fully coherent RCWA (black dashed line) with a period of 3 µm, a gap of 50 nm, and a graphene Fermi level of 0.87 eV. The incoherent model effectively suppresses the Fabry–Pérot interference fringes, smoothly tracking the moving average of the coherent results.

In general, the transfer matrix method (TMM) utilizes a scattering-matrix formalism to describe the propagation of electromagnetic waves through a multilayer structure [12]. The relationship between the electric fields at the first interface and the last interface is expressed as follows:

$$\begin{bmatrix} \mathrm{E}_{0\mathrm{D}}^{+} \\ \mathrm{E}_{0\mathrm{D}}^{-} \end{bmatrix} = \mathbf{S} \begin{bmatrix} \mathrm{E}_{(m+1)\mathrm{U}}^{+} \\ \mathrm{E}_{(m+1)\mathrm{U}}^{-} \end{bmatrix}. \quad (S7)$$

In this expression, E_{0D} denotes the electric field at the boundary between the 0th layer and the 1st layer, where the superscripts + and – indicate propagation in the positive and negative directions, respectively. The 2×2 scattering matrix **S** is defined by the product of interface matrices $\mathbf{I}_{j(j+1)}$ and

layer propagation matrices $\mathbf{P}_j$ as $\mathbf{S} = \mathbf{I}_{01}\mathbf{P}_1\mathbf{I}_{12} \dots \mathbf{P}_m\mathbf{I}_{(m+1)}$. The interface matrix $\mathbf{I}_{j(j+1)}$ accounts for the reflection and transmission coefficients at the boundary of jth and (j+1)th layers, which can be derived from the Fresnel coefficients for uniform layers. The propagation through each layer is governed by the matrix $\mathbf{P}_j$, defined as:

$$\mathbf{P}_j = \begin{bmatrix} \exp(-i\beta_j) & 0 \\ 0 & \exp(i\beta_j) \end{bmatrix} \tag{S8}$$

where $\beta_j = \frac{2\pi d_j N}{\lambda_0}\cos(\phi_j)$ represents the phase shifts, with d_j, N_j, λ_0, φ_j being the layer thickness, complex refractive index, free-space wavelength, and propagation angle, respectively. From this scattering matrix, the front and back reflection (r, r') and transmission (t, t') coefficients for the entire structure can be extracted.

The device investigated in this study consists of seven distinct layers, including the incident air, ionic gel, Au grating, Ti grating, graphene (Gr) grating, CaF_2 substrate, and the transmitted air layer as shown in Figure S12a. Among these components, the CaF_2 substrate is treated as an incoherent layer due to its large optical thickness as well as surface roughness and slight non-parallelism, which eliminate internal interference effects. To account for this, the structure is divided into coherent packets separated by the incoherent substrate. Following the generalized matrix method, the coherent scattering matrix $\mathbf{S}$ for the layers preceding the CaF_2 is converted into an equivalent incoherent interface matrix $\bar{\mathbf{S}}$ based on intensity-related components [13] $\mathrm{U} = |\mathrm{E}|^2$:

$$\bar{\mathbf{I}} = \frac{1}{|t|^2}\begin{bmatrix} 1 & -|r'|^2 \\ |r|^2 & |tt'|^2 - |rr'|^2 \end{bmatrix}, \tag{S9}$$

$$\bar{\mathbf{P}} = \begin{bmatrix} \left|\exp(-i\beta_j)\right|^2 & 0 \\ 0 & \left|\exp(i\beta_j)\right|^2 \end{bmatrix}. \tag{S10}$$

By substituting the complex electric field matrices with these real-valued intensity matrices, the mixed coherent-incoherent TMM allows for an accurate calculation of the total transmittance and reflectance without the Fabry–Pérot interference typically caused by the thick substrate in a fully coherent model.

The aforementioned interface and propagation matrices based on Fresnel coefficients are only suitable for structures composed of uniform layers, such as the material fitting cases discussed in Supplementary Note 3 or the case for the grating structure is being approximated to equivalent surface admittance using circuit model. However, they cannot be directly applied to complex, patterned structures, which need to be simulated through full-wave simulations such as Rigorous Coupled-Wave Analysis (RCWA). To treat CaF_2 substrate incoherently within the RCWA framework as well, we implemented an RCWA-incorporated incoherent calculation.

In this approach, the reflection r and transmission t coefficients of the metasurface's coherent packet are first rigorously calculated using RCWA. The incoherent calculation becomes more complex in RCWA as it must account for multiple diffraction orders. Specifically, the total transmittance T_{tot} through the combined system of the grating and the incoherent substrate is determined by summing the power of all orders, considering the infinite internal reflections within the substrate without phase coherence. This RCWA-incorporated incoherent propagation can be expressed as a geometric series of the power matrices:

$$\mathbf{T}_{\mathrm{tot}} = \overline{\mathbf{T}}_{\mathrm{surf}}\overline{\mathbf{P}}_{\mathrm{CaF_2}}\left(\mathbf{I} - \overline{\mathbf{R}}_{\mathrm{grat}}\overline{\mathbf{P}}_{\mathrm{CaF_2}}\overline{\mathbf{R}}_{\mathrm{surf}}\overline{\mathbf{P}}_{\mathrm{CaF_2}}\right)^{-1}\overline{\mathbf{T}}_{\mathrm{grat}}. \quad \text{(S11)}$$

Here, $\overline{\mathbf{T}}_{\mathrm{surf}}$, $\overline{\mathbf{R}}_{\mathrm{surf}}$ represent the $n\times o$ transmission and $o\times o$ internal reflection matrices at the air-CaF_2 interface (viewed from inside the substrate), respectively. $\overline{\mathbf{T}}_{\mathrm{grat}}$, $\overline{\mathbf{R}}_{\mathrm{grat}}$ are the $o\times 1$ forward transmission and $o\times o$ backward reflection response matrices of the coherent packet obtained via RCWA. $\overline{\mathbf{P}}_{\mathrm{CaF_2}}$ is the $o\times o$ propagation matrix within the CaF_2 substrate, where o and n represent the number of available diffraction orders within the CaF_2 substrate and transmitted air layer, respectively. In these matrices, the element in the i-th row and j-th column denotes the transmittance or reflectance scattered from the j-th incident diffraction order into the i-th diffracted order.

Figure S12b validates the proposed RCWA-incorporated incoherent calculation, demonstrating that its results for the device with a period of 3 μm, a gap of 50 nm, and a graphene Fermi level of 0.87 eV, closely track the moving average of the fully coherent RCWA spectrum. For the RCWA calculations, RETICOLO V9, an open-source MATLAB RCWA library [14], with a Fourier order of 425 was used.

Supplementary Note 7. Fitting procedure for the graphene Fermi level

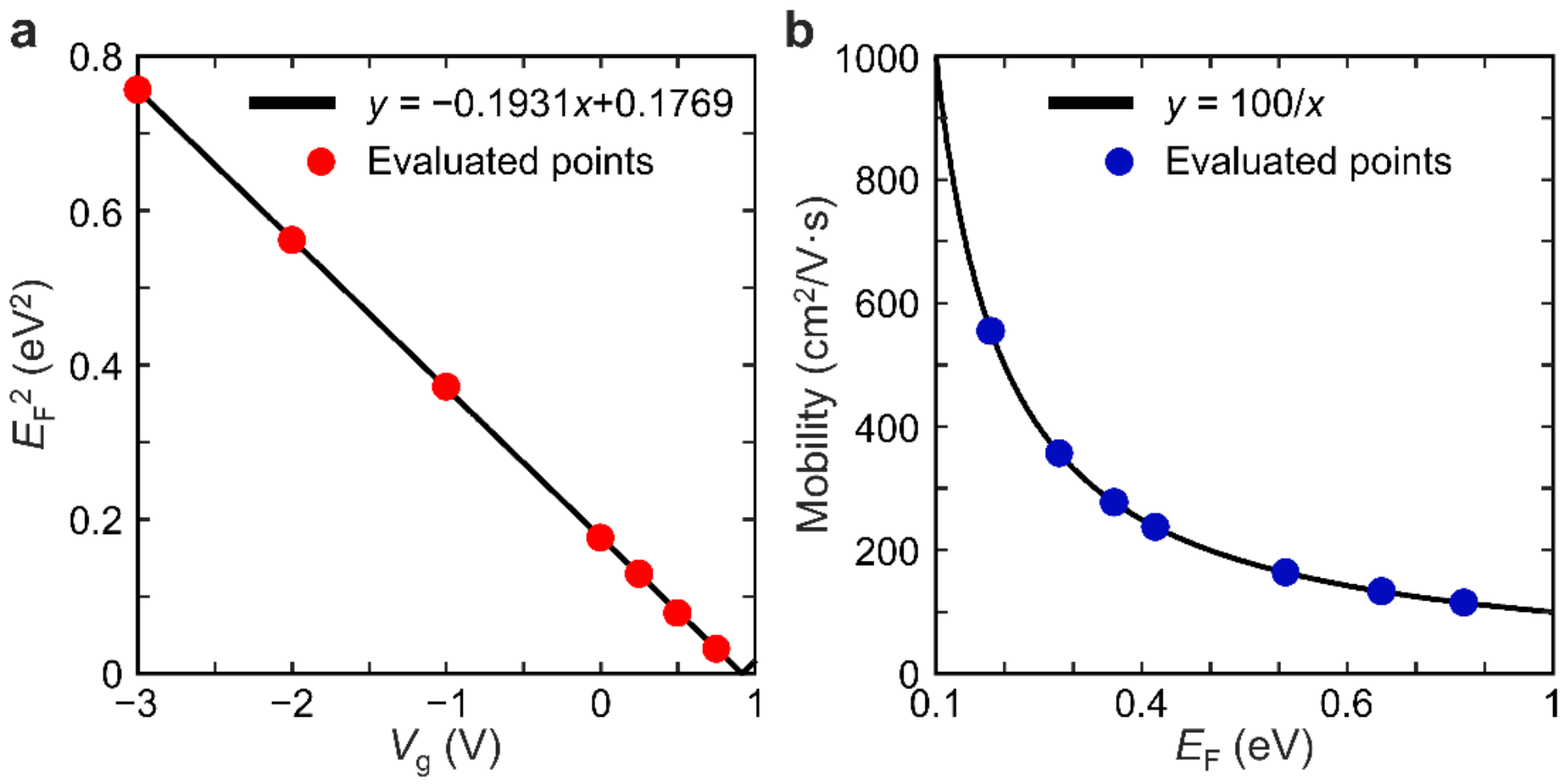

Figure S13. Optimization results for the graphene Fermi level and carrier mobility. a The square of the extracted Fermi level (E_F^2) as a function of the applied gate voltage (V_g). The red circles indicate the evaluated points from the optimization, which strictly follow the analytical linear fit (black solid line, $E_F^2 = -0.1931V_g + 0.1769$). **b** The corresponding graphene carrier mobility (μ) as a function of the Fermi level. The blue circles represent the evaluated points, constrained by the constant scattering condition of $E_F \times \mu = 100$ (black solid line). The evaluated E_F values of [0.87, 0.75, 0.61, 0.42, 0.36, 0.28, 0.18] eV and mobilities of [116, 134, 166, 240, 281, 356, 564] cm^2/V·s correspond to the gate voltages of [−3, −2, −1, 0, 0.25, 0.5, 0.75] V.

To extract the graphene Fermi level E_F from the measured transmittance spectra, a global optimization using a pre-calculated simulation library was performed. The experimental data consisted of transmission spectra for devices with varying periods ($p \in [1,\ 2,\ 3,\ 4,\ 5]$ μm, $g = 50$ nm) and gap sizes ($p = 3$ μm, $g = [50, 75, 100, 125, 150]$ nm) under seven distinct gate voltages ($V_g \in [-3, -2, -1, 0, 0.25, 0.5, 0.75]$ V). The discrete simulation library calculated from the circuit model was constructed by sweeping E_F from 0.02 eV to 1.0 eV in steps of 0.02 eV, and the carrier mobility μ from 50 cm^2/V·s to 1500 cm^2/V·s in steps of 5 cm^2/V·s.

To prevent overfitting and ensure physical validity, a constant scattering assumption was applied by fixing the product of E_F and μ to $E_F \times \mu = 100$, which is equivalent to a constant graphene scattering rate of $\Gamma = 0.066$ eV. Consequently, the electrical modulation of E_F was modeled using the analytical relation $E_F = \sqrt{|a \cdot V_g + b|}$, which describes the dependence of the charge carrier density on the gate voltage near the Dirac point. This physical constraint reduces the optimization variables to only two fitting parameters: the slope a and the offset b. The continuous search space was tightly bounded to $a \in [-0.5, 0]$ and $b \in [0, 1]$.

Prior to the error evaluation, a dynamic frequency masking technique was applied to exclude high-frequency diffraction effects from the experimental data, because the adopted circuit model, chosen for computational efficiency, cannot account for these diffraction phenomena. A specific cutoff frequency, determined by the diffraction limit at a 45-degree incidence angle for each periodicity p, was utilized as it is the maximal acceptance angle for FTIR measurements. The discrepancy between the masked experimental data and the simulated spectra was then evaluated using a dual-objective mean absolute error (MAE) loss function, expressed as:

$$Loss = MAE\left(T_{\text{exp}} - T_{\text{sim}}\right) + MAE\left(T_{\text{exp}} - T_{\text{sim}} \times \frac{\max\left(T_{\text{exp}}\right)}{\max\left(T_{\text{sim}}\right)}\right). \tag{S12}$$

This function simultaneously minimizes the raw MAE to capture baseline transmittance fluctuations and the maximum-normalized MAE to track resonance frequency shifts and spectral lineshapes. To balance their physical weighting, the normalized MAE was scaled by the maximum experimental transmittance.

Finally, to avoid local minima caused by the discrete simulation grid, the MATLAB's built-in MultiStart global optimization algorithm was employed. The optimization was executed with 1,000 independent loops starting from randomly generated initial points within the bounded parameter space. Figure S13 illustrates the result of the optimization, where a linear relation between E_{F}^2 and V_{g} is clearly observed, satisfying the relation $E_{\text{F}}^2 = -0.1931V_{\text{g}} + 0.1769$. Specifically, gate voltages of [−3, −2, −1, 0, 0.25, 0.5, 0.75] V are mapped to Fermi level of [0.87, 0.75, 0.61, 0.42, 0.36, 0.28, 0.18] eV within the p-doped gating regime. These Fermi levels, rounded to the third decimal place, were subsequently used in the following simulations. Figures S14 and S15 provide a comparison between the measured spectra and reconstructed spectra calculated from the circuit model, using the fitted Fermi levels and graphene mobilities. While the absolute transmittance intensities may exhibit slight deviations due to the assumption of a single constant scattering rate to prevent overfitting, the overall spectral trends including peak frequency, across varying gate voltages (Fermi levels) show excellent agreement.

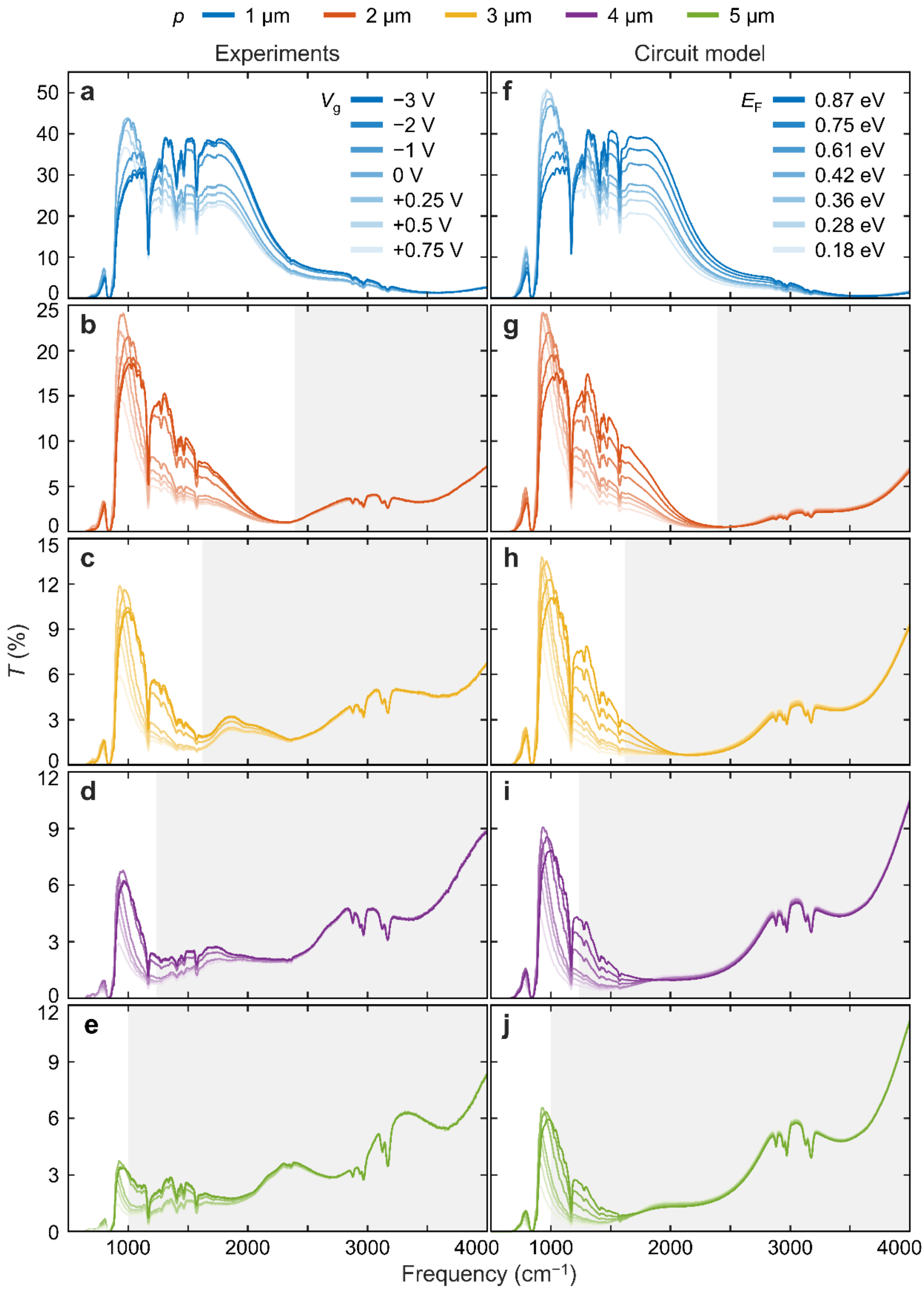

Figure S14. Experimental and simulated transmission spectra of the period-sweep devices. a–e Measured transmission spectra for devices with varying periods (p = 1, 2, 3, 4, 5 μm, g = 50 nm) as a function of the applied gate voltage. **f–j** Reconstructed transmission spectra calculated using the circuit model with the fitted Fermi levels and mobilities. The grey shaded areas represent the frequency regimes excluded by the dynamic frequency masking to prevent diffraction-induced fitting errors.

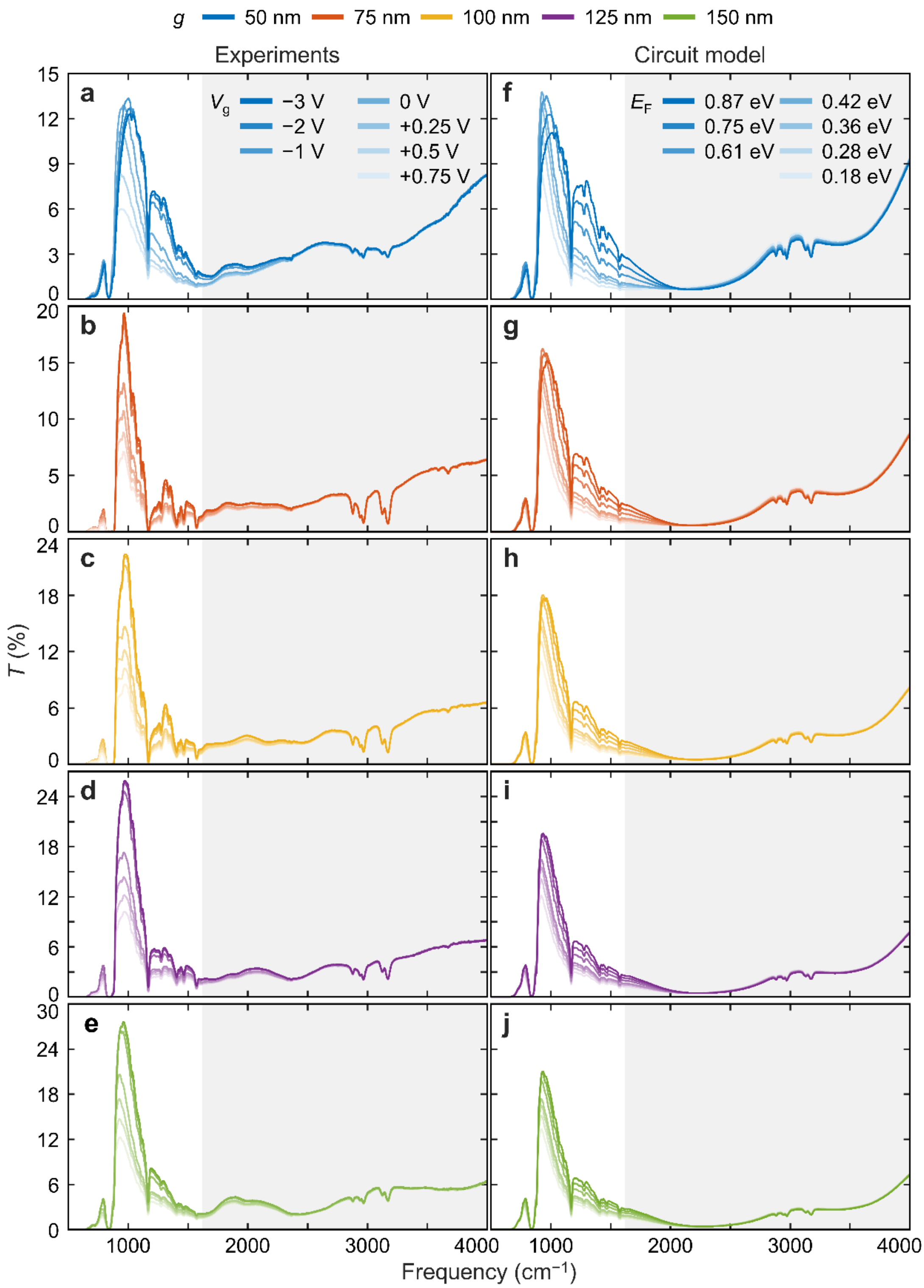

Figure S15. Experimental and simulated transmission spectra of the gap-sweep devices. a–e Measured transmission spectra for devices with varying periods (p = 3 μm, g = 50, 75, 100, 125, 150 nm) as a function of the applied gate voltage. **f–j** Reconstructed transmission spectra calculated using the circuit model with the fitted Fermi levels and mobilities. The grey shaded areas represent the frequency regimes excluded by the dynamic frequency masking to prevent diffraction-induced fitting errors.

Supplementary Note 8. Full-wave RCWA simulations at normal incidence

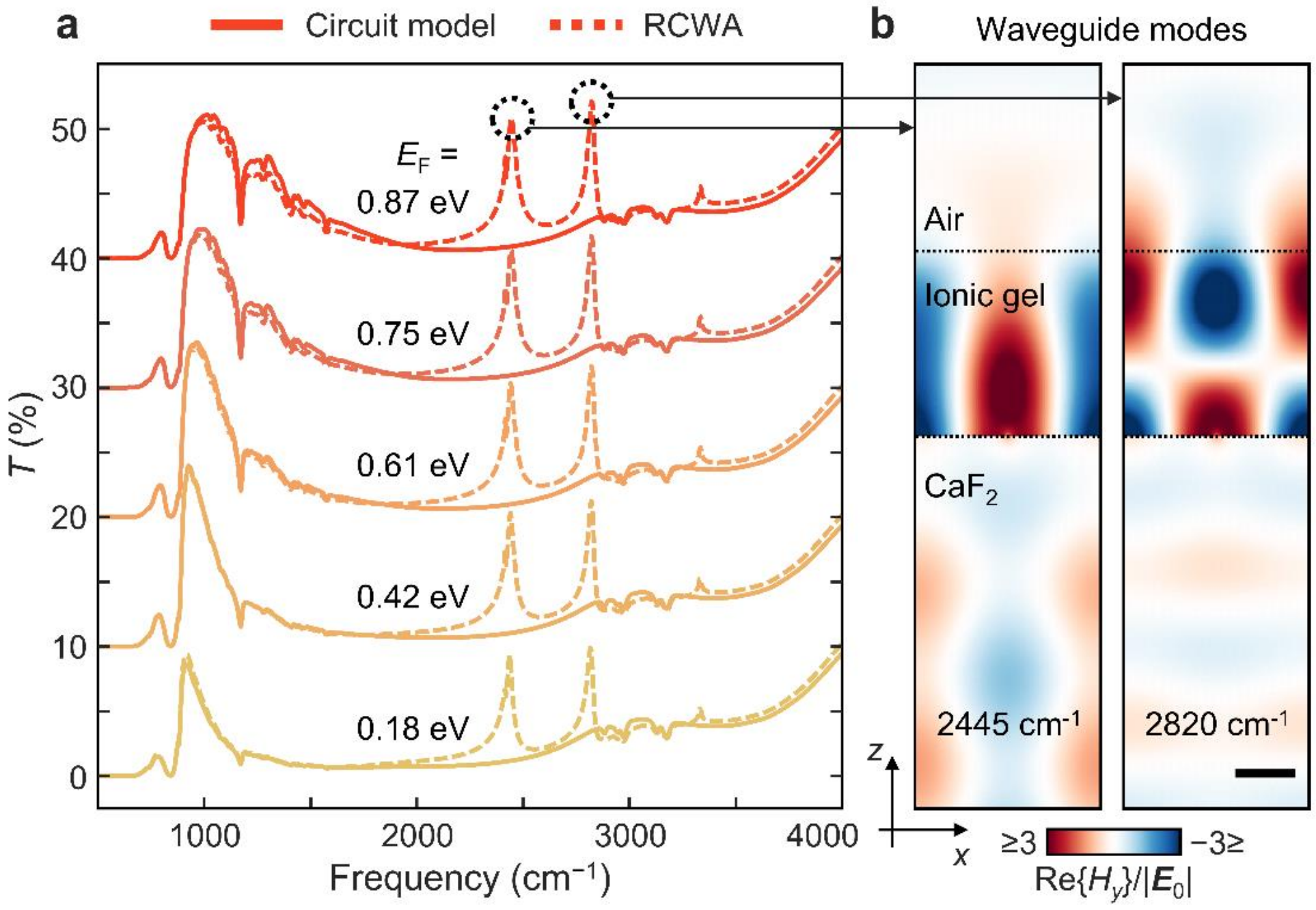

Figure S16. Comparison of the circuit model and full-wave RCWA simulations. a Calculated transmission spectra for the device (p = 3 μm, g = 50 nm) using the circuit model (solid lines) and RCWA (dotted lines) under various graphene Fermi levels. **b** Cross-sectional magnetic field profiles (Re{H_y}) corresponding to the major waveguide mode peaks at 2445 cm^{-1} and 2820 cm^{-1}.

To validate the accuracy of the circuit model, we compared the analytically calculated transmission spectra with full-wave RCWA simulations. The calculations were performed for the structure with a period of 3 μm and a gap of 50 nm under TM-polarized normal incidence. For the RCWA calculations, Fourier orders of 425 were used to ensure numerical convergence.

As shown in Figure S16a, the overall baseline transmission and the modulation trends of the resonances exhibit excellent agreement between the two models across the varying Fermi levels. However, noticeable discrepancies emerge in the high-frequency regime between 2000 cm^{-1} and 3500 cm^{-1}. Because the circuit model relies on an effective surface admittance model combined with TMM calculations, it inherently cannot account for multiple diffractions and waveguide coupling effects. The RCWA spectra reveal two major sharp resonance peaks at 2445 cm^{-1} and 2820 cm^{-1}. As visually confirmed by the cross-sectional magnetic field profiles in Figure S16b, these prominent features correspond to the excitation of the first- and second-order waveguide modes tightly confined within the ionic gel layer. Furthermore, the two minor peaks observed at 2415 cm^{-1} and 3335 cm^{-1} originate from the Rayleigh anomalies, representing the onset of diffraction orders propagating into the CaF_2 substrate and the air layer, respectively.

To clarify the validity range of the equivalent-circuit model, we carried out full-wave RCWA simulations for all period- and gap-dependent metasurfaces investigated in this work. The comparison results are presented in Figures S17 and S18. As shown in the figures, the equivalent-circuit model accurately reproduces the resonance frequencies, transmission spectra, and geometry-dependent modulation characteristics over the entire investigated geometrical range (p = 1-5 µm and g = 50-150 nm). As the metasurface period increases, the diffraction-related Rayleigh anomalies and ionic-gel waveguide modes progressively redshift toward lower frequencies. Since these effects are not included in the equivalent circuit model, discrepancies with RCWA simulations appear once they enter the spectral range of interest. Consequently, the validity range of the equivalent circuit model becomes increasingly restricted for larger periods.

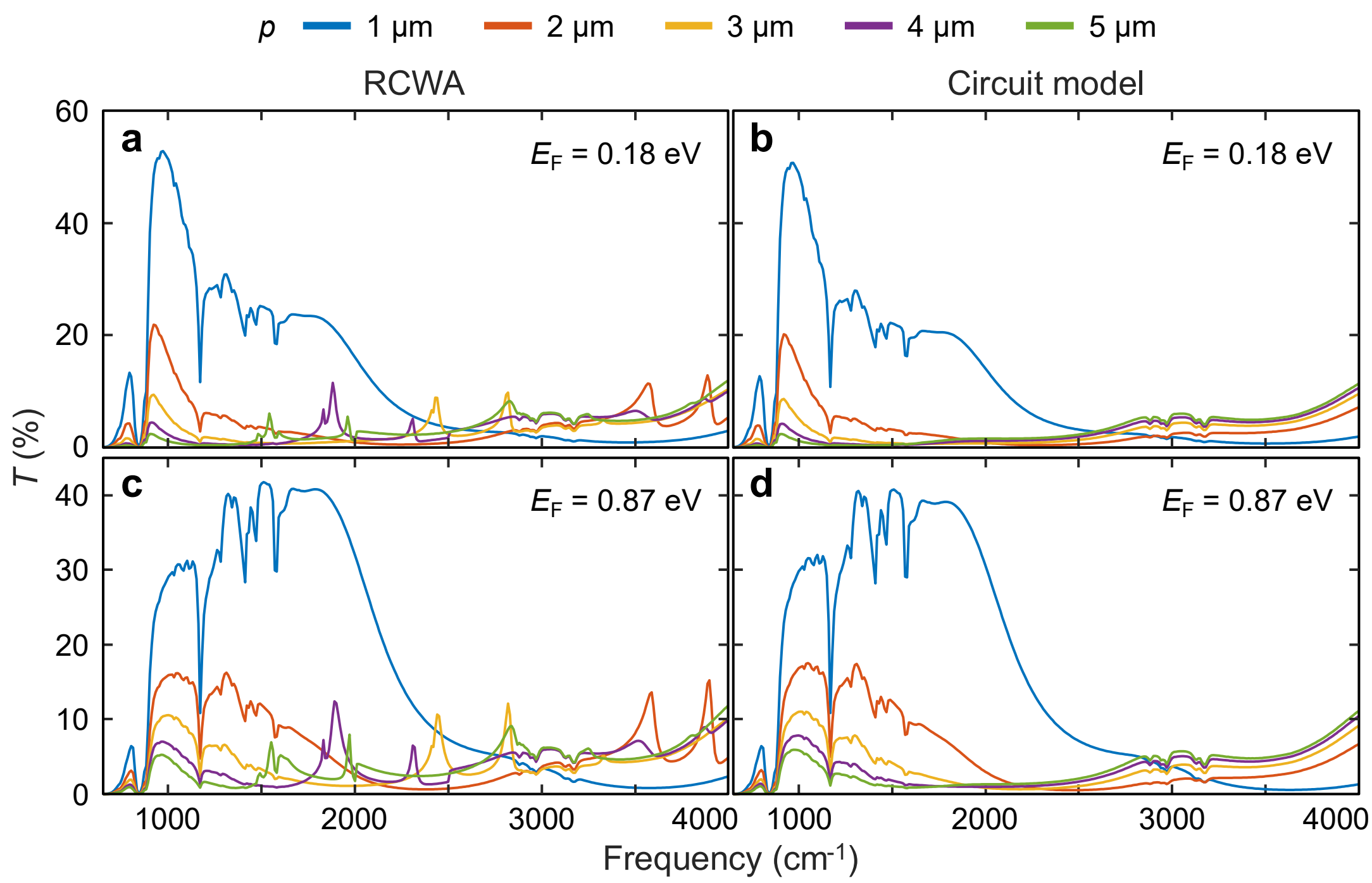

Figure S17. Comparison between full-wave RCWA simulations and the equivalent-circuit model for period-dependent metasurfaces (p = 1-5 µm, g = 50 nm). a RCWA simulations at E_F = 0.18 eV. **b** Equivalent-circuit model at E_F = 0.87 eV. **c** RCWA simulations at E_F = 0.87 eV. **d** Equivalent-circuit model at E_F = 0.87 eV.

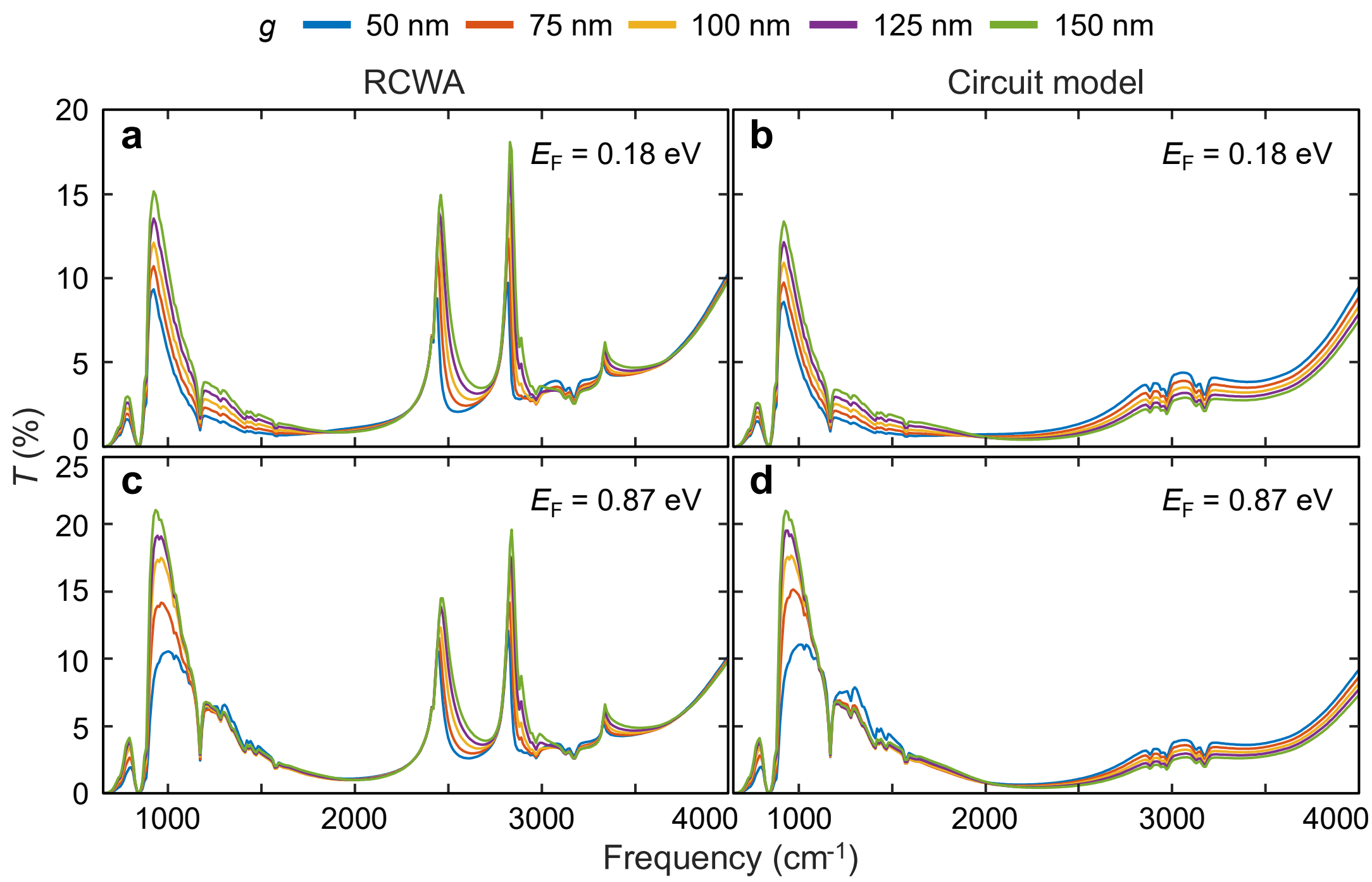

Figure S18. Comparison between full-wave RCWA simulations and the equivalent-circuit model for gap-dependent metasurfaces (p = 3 µm, g = 50-150 nm). a RCWA simulations at E_F = 0.18 eV. **b** Equivalent-circuit model at E_F = 0.87 eV. **c** RCWA simulations at E_F = 0.87 eV. **d** Equivalent-circuit model at E_F = 0.87 eV.

Supplementary Note 9. Dependence of transmission on conical incidence angles

9.1 Angle-weighted RCWA calculations

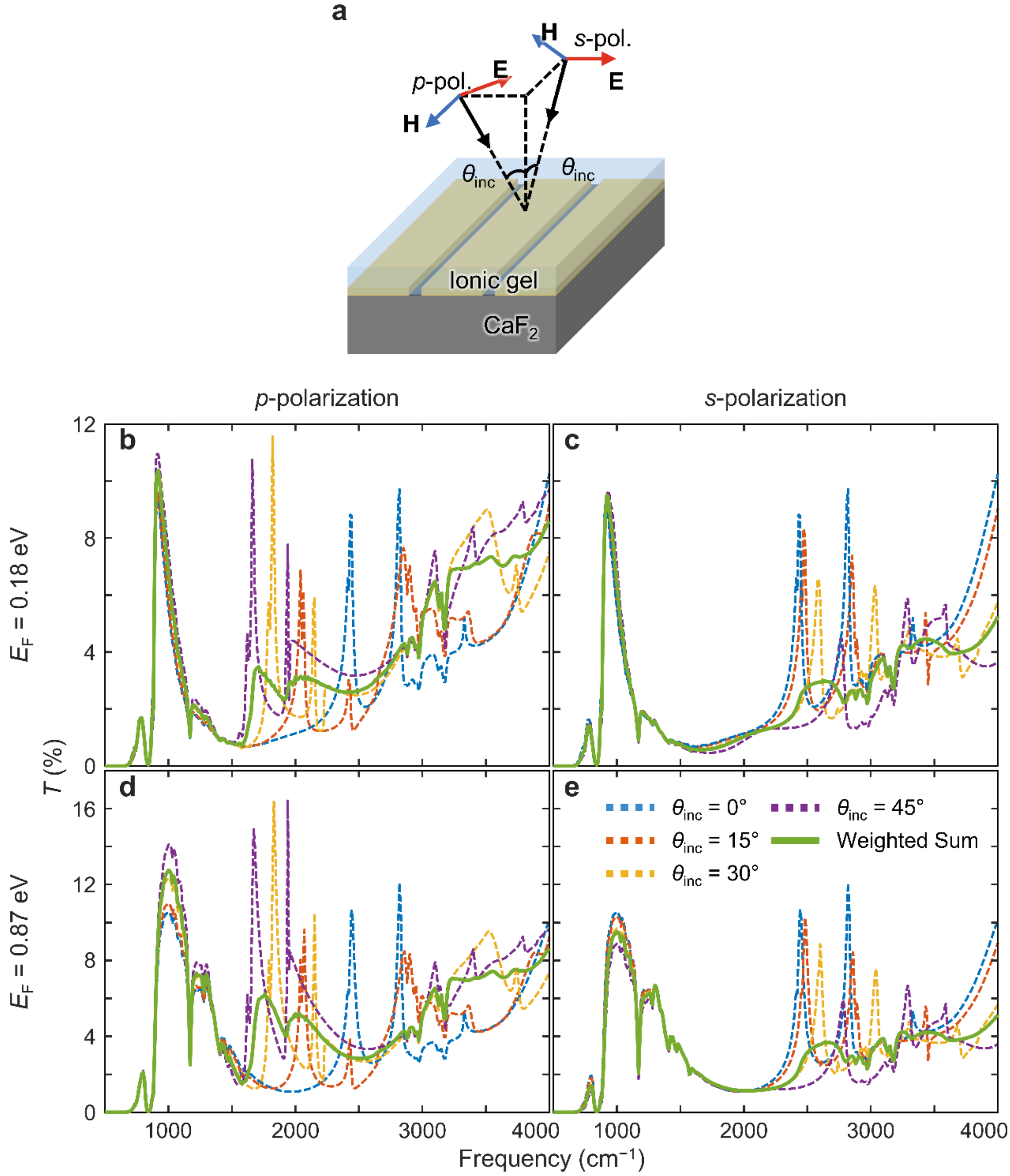

Figure S19. Angle-resolved transmission spectra for conical incidence. a Schematic illustration of the conical incidence geometry with *p*- and *s*-polarizations assuming TM-polarizer. **b–e** Calculated transmission spectra (p = 3 μm, g = 50 nm) at E_F = 0.18eV (b, c) and E_F = 0.87 eV (d, e) for *p*- and *s*-polarizations under various incident angles (θ_{inc}) up to 45°. The solid green lines represent the angle-weighted sum approximating the FTIR measurement with NA = 0.71.

In the main text, the optical responses of the graphene-metal hybrid metasurface were analyzed using normal incidence simulations for simplicity and clear physical interpretation. However, the experimental transmittance was measured using a Fourier Transform Infrared (FTIR) microscope equipped with a Cassegrain objective (NA = 0.71). This setup illuminates the sample with a converging conical beam with incident angles θ_{inc} up to 45°. To rigorously validate our simplified normal-incidence model and fully reproduce the measured FTIR spectra, we performed angle-resolved RCWA simulations taking this conical incidence into account.

To simulate the conical illumination, the full-wave RCWA calculations were conducted over an incident angle range of θ_{inc} = 0° to 45° with an angular resolution of $\Delta\theta$ = 1°. Two orthogonal azimuthal planes (0° and 90°) were chosen to represent the *p*- and *s*-polarizations, respectively, and the results were averaged to account for the FTIR light source with TM-polarizer, as shown in Figure S19a–e. To mimic the light collection geometry of the FTIR microscope lens, the angle-weighted total transmittance was calculated using a weighting factor $W(\theta) = \tan(\theta)\sec^2(\theta)\Delta\theta$.

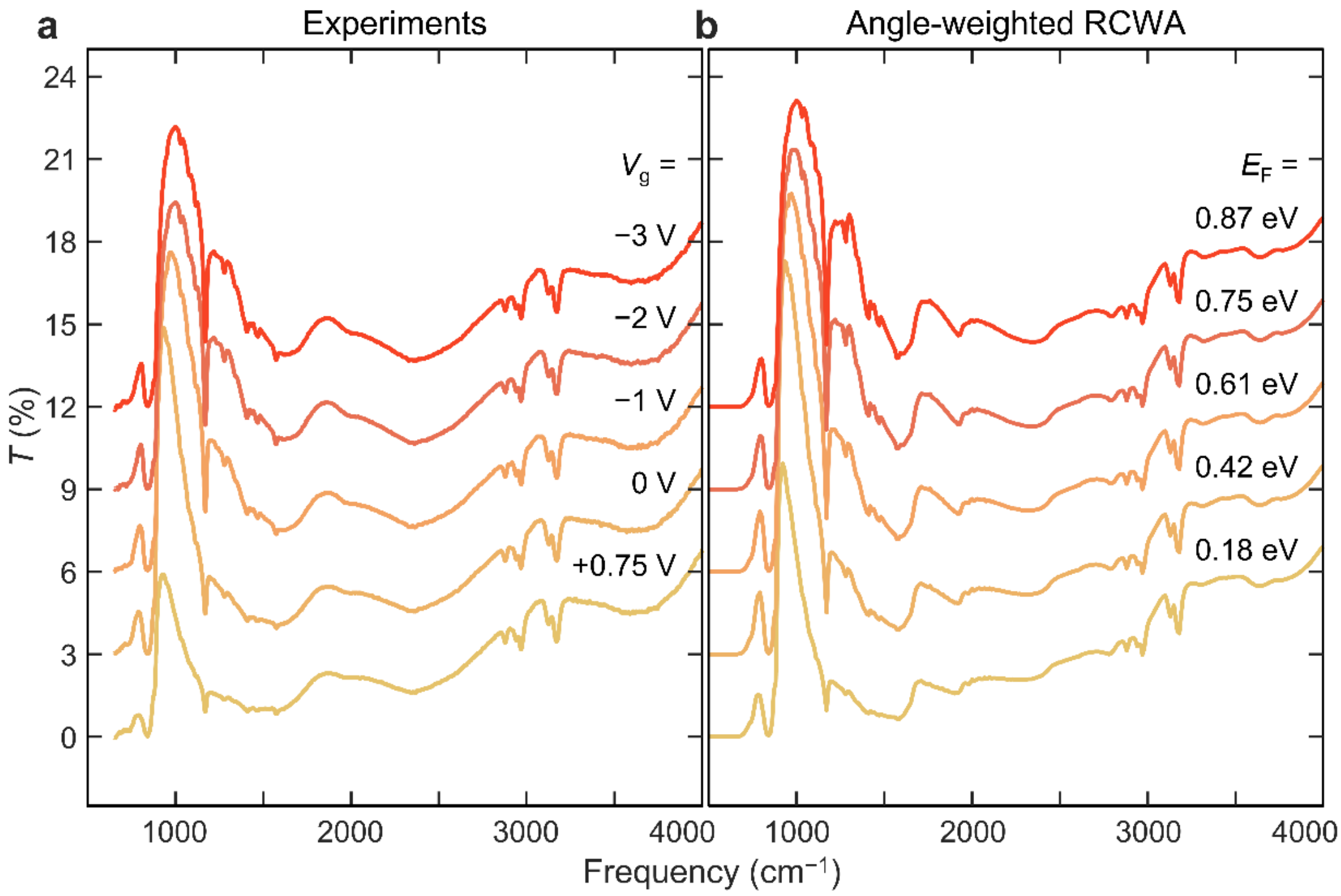

Figure S20. Comparison of measured FTIR spectra and angle-weighted RCWA. a Experimental transmission spectra (p = 3 µm, g = 50 nm) measured at various gate voltages (V_g). **b** Reconstructed spectra from the angle-weighted RCWA calculations under varying Fermi levels (E_F). The sharp waveguide and diffraction modes are averaged out, faithfully reproducing the smooth experimental curves.

As discussed in Supplementary Note 7, normal incidence calculations exhibit sharp, highly sensitive peaks corresponding to ionic gel waveguide modes and Rayleigh anomalies. However, because the resonance conditions for these modes are strongly angle-dependent, these sharp peaks are

completely averaged out during the angle-weighting process. As a result, the angle-weighted RCWA spectra successfully reproduce the smooth, broadened spectral features observed in the actual FTIR measurements, as illustrated in Figure S20a, b. Most importantly, the overall modulation and the parallel LC resonance tuning trends remain largely consistent with the normal incidence case. This confirms that the normal incidence approximation used in the main text is sufficiently robust and valid for explaining the primary modulation mechanisms of the device.

9.2 Fabry-Pérot interference and gel thickness variations

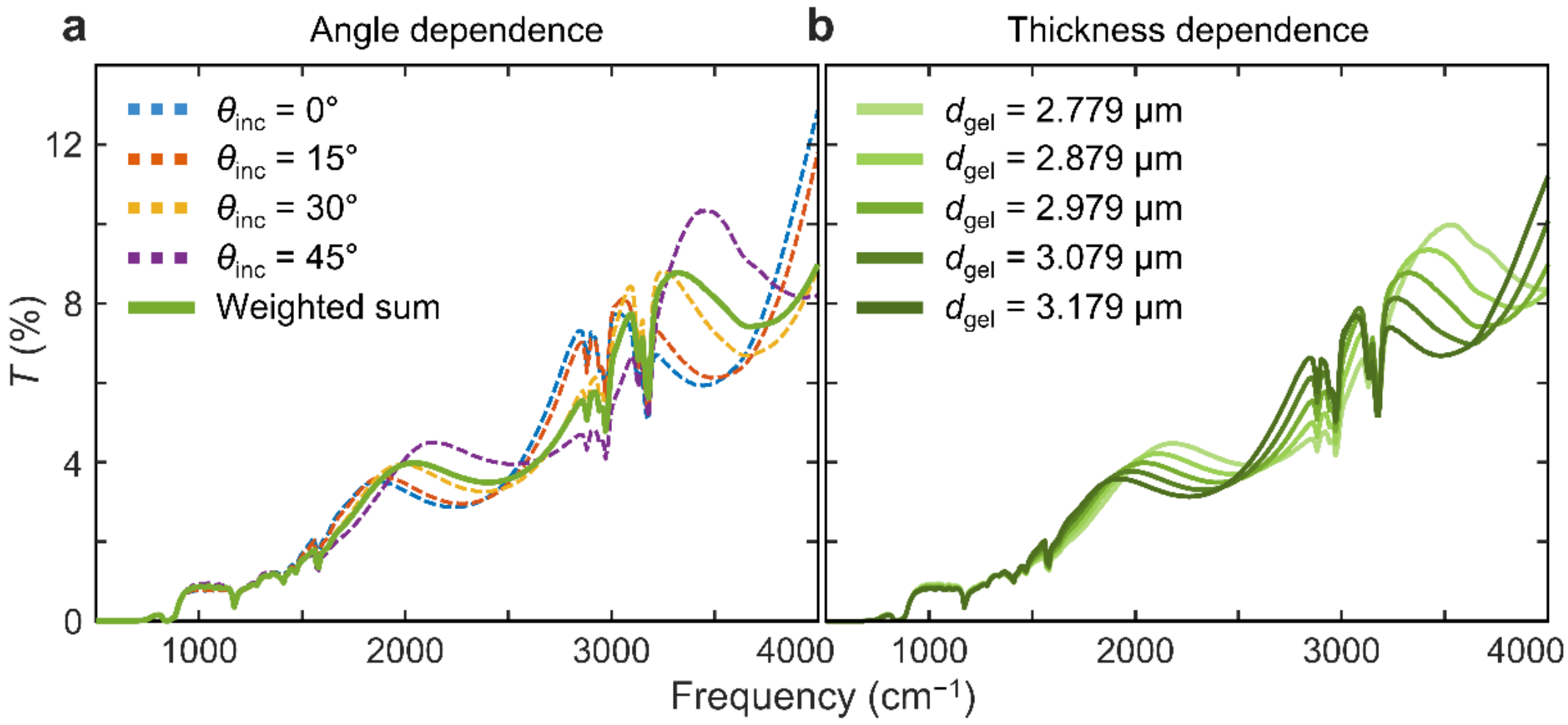

Figure S21. Effect of conical incidence and ionic gel thickness on Fabry-Pérot oscillations. a Angle dependence of the transmission spectra, showing the shift of Fabry-Pérot (FP) interference patterns in the high-frequency regime. **b** Transmission spectra variations calculated with different ionic gel thicknesses (d_{gel}), demonstrating that a minor thickness deviation of 100 nm significantly alters the position of the FP oscillations.

While the angle-weighted RCWA successfully smoothens the sharp diffraction peaks, minor discrepancies in the oscillating peak positions remain between the experiments and the circuit model in the high-frequency regime (>2000 cm^{-1}, see Figures S14 and S15). These broad oscillating patterns originate from the Fabry-Pérot (FP) interference within the ionic gel layer. The exact spectral position of these FP patterns is highly sensitive to the angle of incidence and the precise thickness of the dielectric layers. For this analysis, the theoretical transmission was calculated using a TMM that treats the 500 μm-thick CaF_2 substrate as an incoherent layer, covered by a 2 nm Ti adhesion layer, a 10 nm Au film, and a 2.979 μm-thick ionic gel layer. As the incident angle increases, the resonance positions of the FP cavities continuously shift, meaning that the angle-weighted FP patterns will inherently exhibit a slight spectral offset compared to the simple normal incidence prediction, as shown in Figure S21a. Furthermore, due to the practical tolerances of the spin-coating process, the actual thickness of the ionic gel d_{gel} may vary marginally depending on the local measurement position on the metasurface array or between different fabricated samples. As demonstrated in Figure S21b, even a minute thickness

deviation of ~100 nm in the ionic gel layer is sufficient to significantly alter the phase and spectral position of the weighted FP oscillations. Given the combination of angle-averaging effects and typical fabrication tolerances, the slight mismatch in the high-frequency oscillating patterns between the idealized normal-incidence model and the measured FTIR spectra is fully expected and physically justified.

Supplementary Note 10. Equivalent circuit model components

The electrically tunable graphene-metal hybrid metasurfaces were analyzed by modeling the structure as a collection of circuit elements [15]. The circuit components that make up the meta structure are as follows. The movement of charge inside the metal is represented by two inductors, while the movement of charge within the grating is represented by two capacitors. These elements allow us to express the movement of charge in the metal structure. The equations for each component are as follows:

Gap capacitance (C_g): This represents the capacitance between the gap formed by the metal grating. It is given by:

$$C_{\mathrm{g}} = \varepsilon_0 \varepsilon_{\mathrm{r,gel}} \frac{h}{g} \tag{S13}$$

where ε_0 is the permittivity of free space, $\varepsilon_{r,gel}$ is the relative permittivity of the ionic gel, h is the height of the metal.

Coplanar capacitance (C_c): This capacitance represents the capacitive component between two infinitely long thin metals. This expression is given by:

$$C_{\mathrm{c}} = \frac{2\varepsilon_0 \varepsilon_{\mathrm{eff}}}{\pi} \ln \frac{p}{g} \tag{S14}$$

where $\varepsilon_{\mathrm{eff}} = (\varepsilon_{\mathrm{r,gel}} + \varepsilon_{\mathrm{r,CaF_2}})/2$ is the effective relative permittivity between ionic gel and CaF_2 substrate.

Magnetic inductance (L_m): The magnetic inductance is generated by the movement of charge between the gap and the metal layers, contributing to the system's inductive response to electromagnetic fields. It is expressed as:

$$L_{\mathrm{m}} = \mu_0 h g \tag{S15}$$

where μ_0 is the permeability of free space.

Kinetic inductance (L_k): The kinetic inductance represents the amount by which charges in the metal are dragged due to their inertial mass, arising from the non-ideal behavior of the metal, thus contributing to the inductive component of the system. The expression is given by:

$$L_{\mathrm{k}} = \frac{p-g}{\varepsilon_0 \delta (1-\varepsilon_{\mathrm{metal}})\omega^2} = \frac{p-g}{\varepsilon_0 h (1-\varepsilon_{\mathrm{metal}})\omega^2} \tag{S16}$$

where δ is skin depth of the metal and ε_{metal} is the relative permittivity of the metal. If the height of the metal is lower than the skin depth, it is possible to replace the δ to h. For Au, δ is about 20–30 nm in the operating frequencies.

Graphene admittance (Y_{Gr}): The graphene admittance is set as the graphene sheet conductivity. Since the voltage drop inside the metal is negligible, we compensate for this by multiplying the graphene admittance by the ratio of period to the gap size. This configuration ensures that the graphene's contribution is aligned with the capacitive component of the gap. The admittance is given by:

$$Y_{\mathrm{Gr}} = \frac{p}{g}\sigma_{\mathrm{Gr}} \tag{S17}$$

where σ_{Gr} is the graphene conductivity.

Supplementary Note 11. Approximations in the circuit model

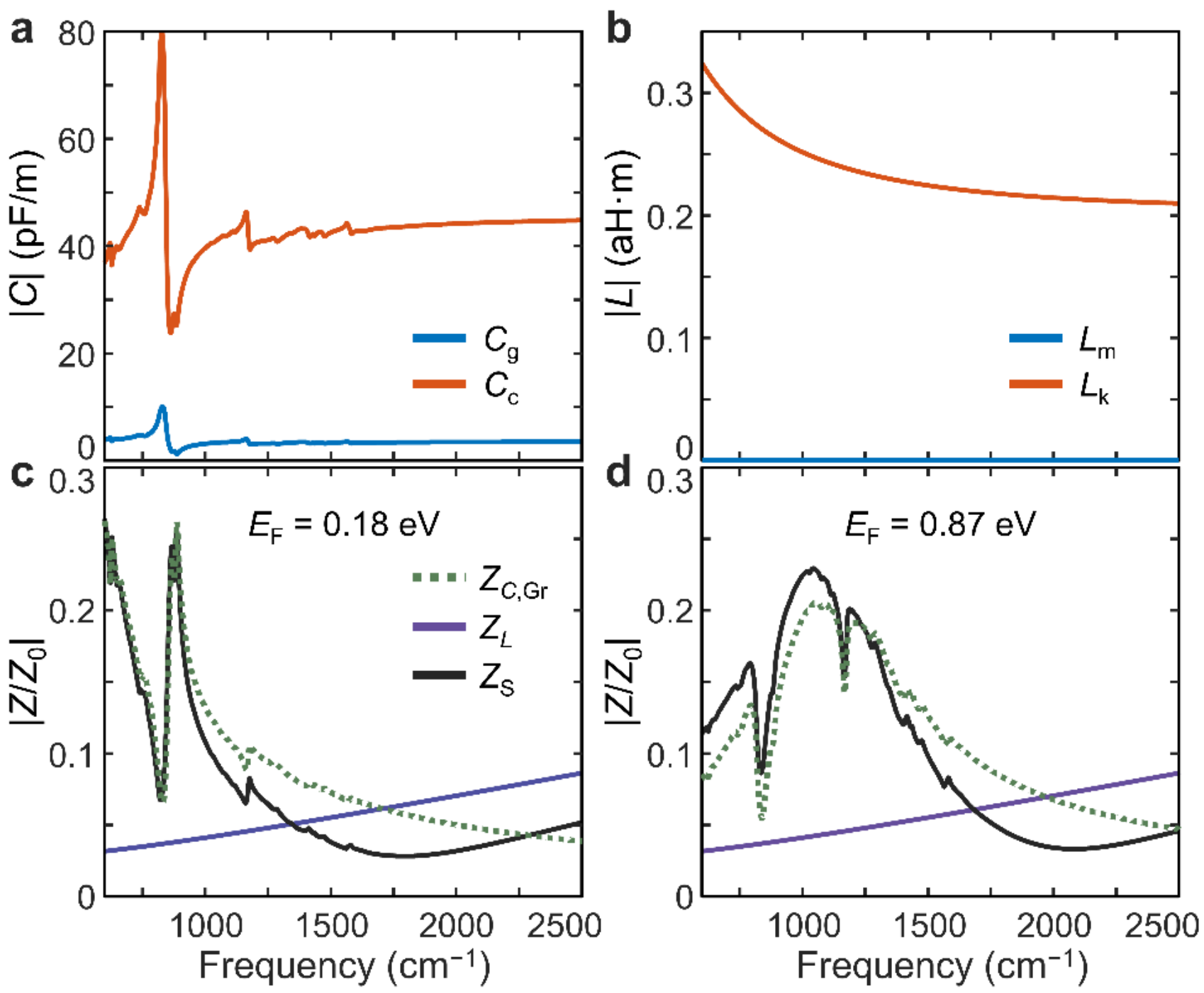

Figure S22. Validation of the analytical approximations used in the circuit model. a Calculated magnitudes of the coplanar capacitance (C_c, orange) and the gap capacitance (C_g, blue), demonstrating that the total capacitance is dominated by C_c. **b** Calculated magnitudes of the kinetic inductance (L_k, orange) and the magnetic inductance (L_m, blue), showing that L_k is the dominant inductive contribution. Period of 3 μm and gap of 50 nm are used as geometrical parameters. **c, d** Normalized magnitudes of the total surface impedance ($|Z_S/Z_0|$, black solid lines), compared with the inductive ($|Z_L/Z_0|$, purple solid lines) and graphene-capacitive ($|Z_{C,Gr}/Z_0|$, green dotted lines) impedance components for graphene Fermi levels of E_F = 0.18 eV (c) and 0.87 eV (d). In the low-frequency regime (ω < 1300 cm^{-1}), the total surface impedance is primarily governed by the graphene-capacitive impedance curve.

In the circuit model analysis, the total capacitance of the metasurface is obtained as the sum of the gap capacitance, $C_g = \varepsilon_0 \varepsilon_{r,gel} \frac{h}{g}$, and the coplanar capacitance, $C_c = \frac{2\varepsilon_0 \varepsilon_{eff}}{\pi} \ln \frac{p}{g}$. Here, the ratio between these two components is given by $C_g/C_c = \pi/2\ (\varepsilon_{r,gel}/\varepsilon_{eff}) \times h/(g \ln p/g)$. Considering the ultrathin nature of the Au film ($h \ll g$) and the large period-to-gap ratio ($g \ll p$), the total capacitance of the metasurface can be effectively approximated by the coplanar capacitance alone, as verified in Figure S22a. Similarly, the ratio between the magnetic inductance and the kinetic inductance is $L_m/L_k = \mu_0 \varepsilon_0 \omega^2 h^2 (g/(p-g)) \times (1-\varepsilon_{metal}) = (2\pi h/\lambda_0)^2 \times (g/(p-g)) \times (1-\varepsilon_{metal})$. This ratio is exceptionally small due to the subwavelength thickness of the Au film ($h \ll \lambda_0$) and the large period-to-gap ratio ($g \ll p$), which is confirmed in Figure S22b.

Consequently, the inductive and capacitive admittances of the metal grating can be simplified as:

$$Y_L \approx \frac{pi}{\omega L_k} \approx \frac{\varepsilon_0 h \omega_{\mathrm{p,metal}}^2}{\omega} i, \tag{S18}$$

$$Y_C \approx -i\omega p C_c = -i\omega \frac{2\varepsilon_0 \varepsilon_{\mathrm{eff}}}{\pi} p \ln \frac{p}{g}, \tag{S19}$$

where the metal permittivity is described using the Drude model with the metal plasma frequency $\omega_{\mathrm{p,metal}}$. From Equations S17 and S18, it is evident that the inductive impedance $Z_L = 1/Y_L$ is significantly smaller than the capacitive impedance $Z_C = 1/Y_C$ in the low-frequency regime. Because these components essentially act in series, the total surface impedance is dominated by the larger component. Therefore, as shown in Figures S22c and d, the magnitude of the total surface impedance $|Z_{\mathrm{S}}|$ closely tracks the magnitude of the graphene-capacitive impedance $|Z_{C,\mathrm{Gr}}| = |1/(Y_C+Y_{\mathrm{Gr}})|$ at frequencies below 1300 cm^{-1}, especially near the LC resonance point.

Supplementary Note 12. Circuit model analysis for an ideal lossless system

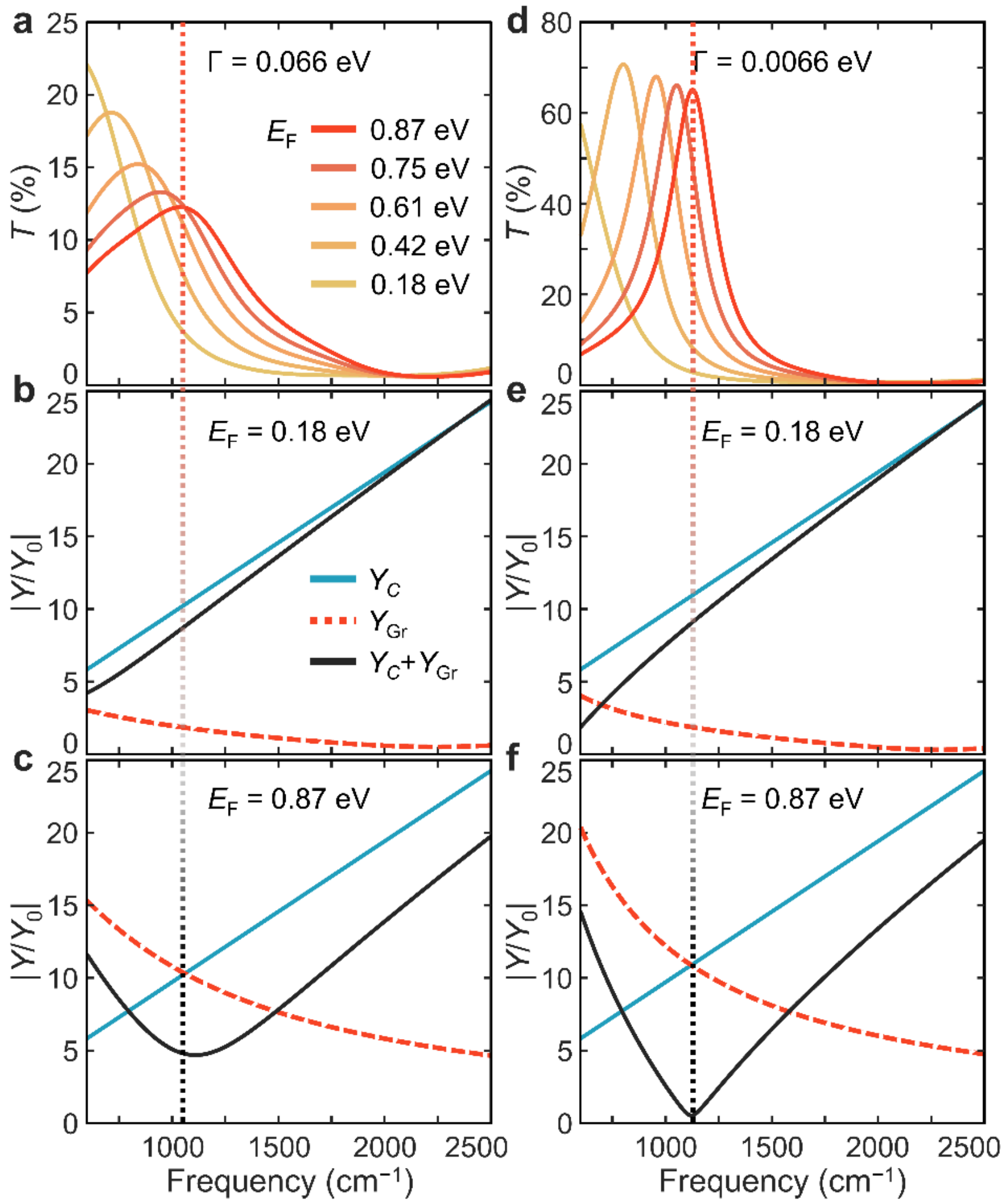

Figure S23. Circuit model analysis for an ideal lossless system. Calculated transmission spectra and admittance magnitudes assuming lossless, non-dispersive surrounding media (n_{gel} = 1.4, n_{CaF2} = 1.3). **a–c** Results using the graphene scattering rate of Γ = 0.066 eV. (a) Transmission spectra for varying Fermi levels. (b, c) Magnitudes of the normalized admittances ($|Y/Y_0|$) for the capacitive metal antenna (Y_C, light blue), the graphene layer (Y_{Gr}, red dashed lines) and their sum (Y_C+Y_{Gr}, black) at E_F = 0.18 eV and 0.87 eV, respectively. **d–f** Results using a ten times reduced graphene scattering rate of Γ = 0.0066 eV. The reduced intrinsic loss leads to a nearly purely imaginary graphene admittance, resulting in a sharper LC resonance cancellation and a significantly enhanced Q-factor in the transmission spectra.

In the actual device, the intrinsic absorption bands and material dispersion of the ionic gel and CaF_2 substrate introduce a non-zero extinction coefficient k. This material loss imparts a real component to the capacitive admittance Y_C, which broadens the resonance and partially obscures the clear observation of the transmission peak shifts caused by the LC resonance. To distinctly isolate the LC resonance mechanism, we performed an additional circuit model analysis assuming an ideal, lossless,

and non-dispersive system. In this ideal model, the refractive indices of the ionic gel and CaF_2 were fixed at constant real values of 1.4 and 1.3, respectively. As shown in Figure S23a–c, removing the substrate and superstrate losses allows the frequency shift of the transmission peak to be much more distinctly observed as the Fermi level increases, unequivocally substantiating our LC resonance tuning mechanism.

Furthermore, we investigated the effect of graphene's intrinsic loss by reducing its scattering rate by a factor of 10 (from 0.066 eV to 0.0066 eV), which conceptually corresponds to a tenfold increase in carrier mobility. When the scattering rate is substantially reduced, the graphene admittance Y_{Gr} becomes nearly purely imaginary with a negligible real part, leading to a near-perfect cancellation with the capacitive admittance. Consequently, as depicted in Figure S23d–f, the Q-factor of the LC resonance significantly increases, resulting in a dramatic enhancement of the transmission peak. Under this optimal condition, the metasurface becomes highly transparent, and the total transmittance approaches that of a bare, uniform multilayer system (air/ionic gel/CaF_2/air) without the metallic grating.

Supplementary Note 13. Analysis of graphene plasmon resonance contributions

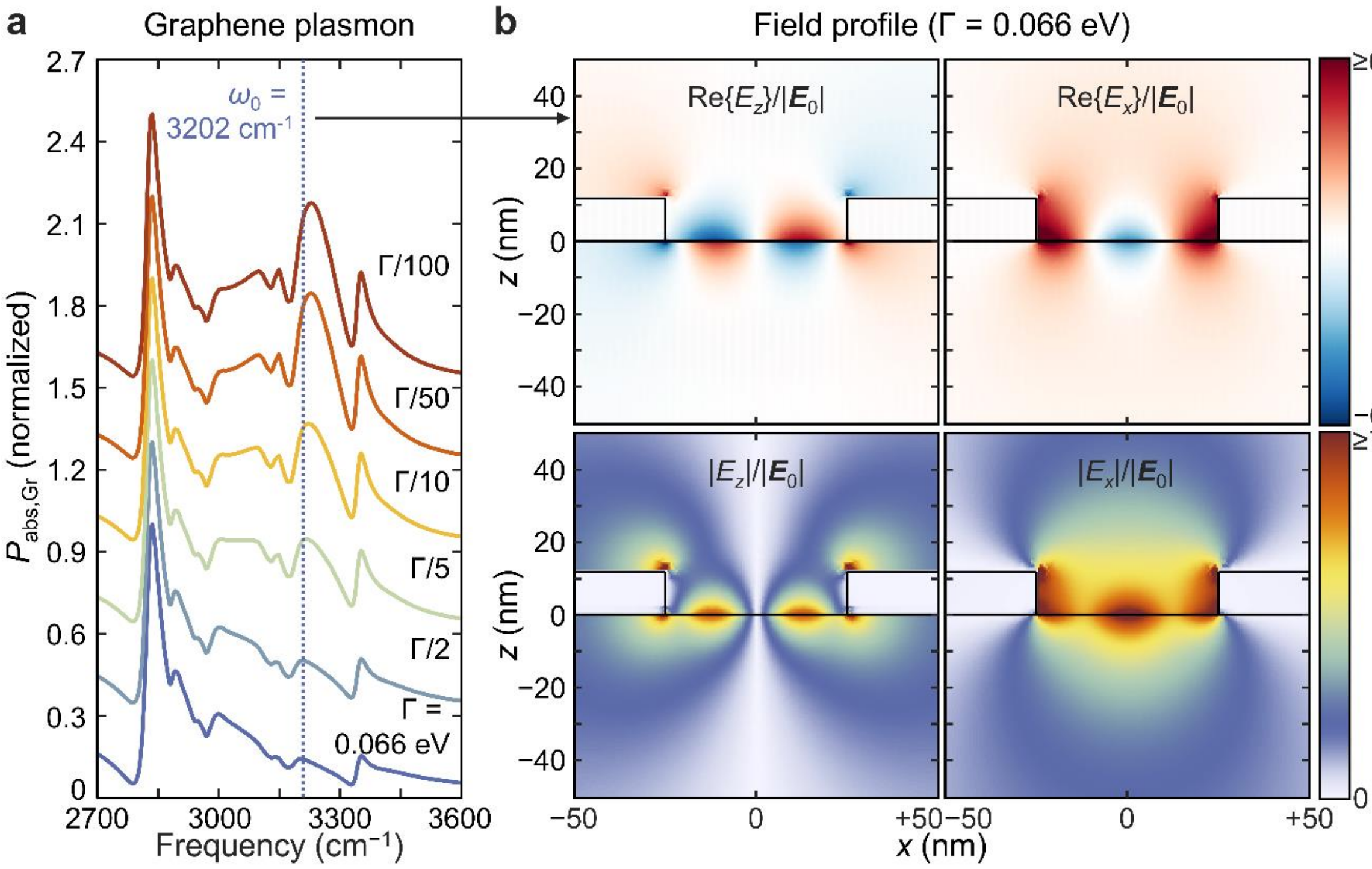

Figure S24. Analysis of graphene plasmon resonance contributions. a Normalized optical absorption power of the graphene layer ($P_{abs,Gr}$) calculated at varying scattering rates under TM normal incidence (E_F = 0.87 eV, p = 3 µm, g = 50 nm). The spectra are normalized to the maximum absorption of the ionic gel waveguide mode at 2820 cm^{-1} and cascaded with vertical offsets of 0.3. As the scattering rate increases to the experimental value (Γ = 0.066 eV), the sharp plasmon peak significantly redshifts and dampens, becoming nearly imperceptible. **b** Simulated near-field profiles (Re{E_x}, Re{E_z}, |E_x|, and |E_z|) at the tracked plasmon frequency of 3202 cm^{-1} with Γ = 0.066 eV, clearly confirming the excitation of a second-order graphene plasmon resonance tightly localized at the metal gap edges.

To verify that the excitation of graphene plasmons does not contribute to our primary transmission tuning mechanism, we investigated the ohmic absorption of the graphene layer across varying scattering rates. Full-wave simulations were conducted using COMSOL Multiphysics for the specific structural condition of p = 3 µm, g = 50 nm, and E_F = 0.87 eV under TM-polarized normal incidence. To isolate the absorption tightly bound to the graphene layer, the exit medium was set to a 5-µm-thick CaF_2 substrate instead of air. The graphene layer was implemented as a surface current density, and its optical absorption power $P_{abs,Gr}$ was calculated by integrating $0.5\mathrm{Re}\{\sigma_{Gr}\}|E_x|^2$ over the sheet boundary. As shown in Figure S24a, the calculated absorption spectra are normalized to the peak intensity of the second-order ionic gel waveguide mode (located at 2820 cm^{-1}) and are vertically cascaded with an offset step of 0.3. For an artificially low scattering rate (Γ/100), a sharp absorption

peak corresponding to the graphene plasmon resonance is clearly visible near ω = 3230 cm^{-1}. However, as the scattering rate increases, this plasmonic peak broadens, redshifts, and severely dampens. When the scattering rate reaches the experimentally fitted value of Γ = 0.066 eV, the plasmon absorption peak becomes virtually imperceptible, completely merging into the background response. By tracking the redshifted peak evolution in the cascade plot, the plasmon resonance frequency at Γ = 0.066 eV was identified at 3202 cm^{-1}. The near-field profiles extracted at this exact frequency, which are provided in Figure S24b, explicitly reveal the excitation of a second-order graphene plasmon mode tightly confined at the gap edges. Note that the first-order plasmon mode remains optically dark and inaccessible under normal incidence due to the symmetry mismatch. Consequently, we conclude that the graphene plasmon resonance is located at 3202 cm^{-1}, which is far detuned from the maximum modulation frequency of our device ω = 1265 cm^{-1}. Furthermore, due to the high intrinsic scattering rate, the plasmonic resonance is heavily overdamped and its influence on the overall transmission modulation is fundamentally negligible.

Supplementary Note 14. Dependance of transmission modulation on geometric parameters

To further investigate the geometric dependence of the LC resonance, we measured the complete TM-polarized transmission spectra for devices with varying periods and gap sizes. Regarding the resonance peak position, since we are primarily interested in the relative magnitude between Y_C and Y_{Gr}, we define a redefined admittance variable $\tilde{Y} = (g/p)\,Y$. This definition ensures that the normalized graphene admittance $\tilde{Y}_{\mathrm{Gr}} = \sigma_{\mathrm{Gr}}$ remains independent of the geometric parameters. With this definition, the surface admittance can be approximated as $Y_s \approx p/g(\tilde{Y}_C + \tilde{Y}_{\mathrm{Gr}})$, allowing us to separate the contributions of resonance condition and overall transmission magnitude. Specifically, the resonance frequency is governed by the condition $\tilde{Y}_C + \tilde{Y}_{\mathrm{Gr}} = 0$, while the absolute magnitude of the surface admittance Y_s is scaled by the geometric factor p/g. Since the transmittance t_S is inversely related to Y_s, an increase in the p/g (either by increasing p or decreasing g) leads to a higher overall surface admittance, which results in a reduction of the absolute transmission level across the entire spectrum.

14.1 Period dependence

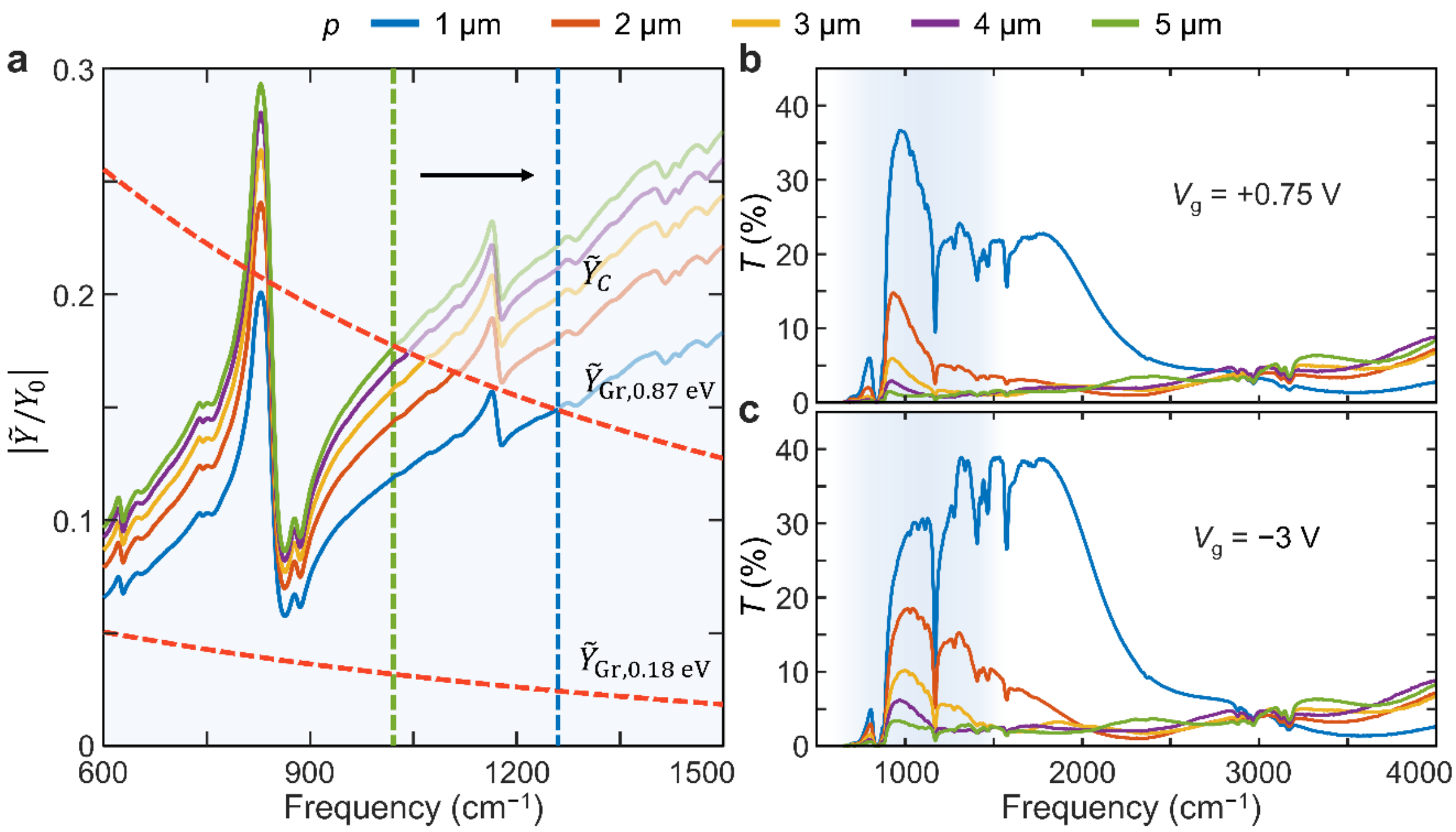

Figure S25. Period dependence on LC resonance. a Calculated normalized admittance $|\tilde{Y}/Y_0|$ of the capacitive metal antenna ($\tilde{Y}_C$, solid lines) and graphene ($\tilde{Y}_{\mathrm{Gr}}$, red dashed lines) for varying periods (p = 1–5 μm, g = 50 nm). Two $\tilde{Y}_{\mathrm{Gr}}$ lines represent the CNP and E_F = 0.87 eV case, respectively. **b, c** Measured transmission spectra for TM-polarized light of devices with a fixed gap size of 50 nm and periods ranging from 1 μm to 5 μm at an applied gate voltage of (b) −3 V and (c) +0.75 V.

Figures S25a presents the calculated $\tilde{Y}$ components extracted from the equivalent circuit model for devices with varying period at the CNP and E_F = 0.87 eV. Under a fixed Fermi level, the graphene admittance $\tilde{Y}_{Gr}$ remains constant regardless of the period. In contrast, the capacitive admittance $\tilde{Y}_C$, which is primarily determined by the coplanar capacitance, is proportional to $\omega g \ln p/g$, showing a decrease in slope as p decreases. This reduction in the capacitive slope leads to a blueshift of the intersection point between $|\tilde{Y}_{Gr}|$ and $|\tilde{Y}_C|$. Consequently, the transmission peak shifts toward higher frequencies as p decreases, as shown in Figures S25b and S25c.

14.2 Gap dependence

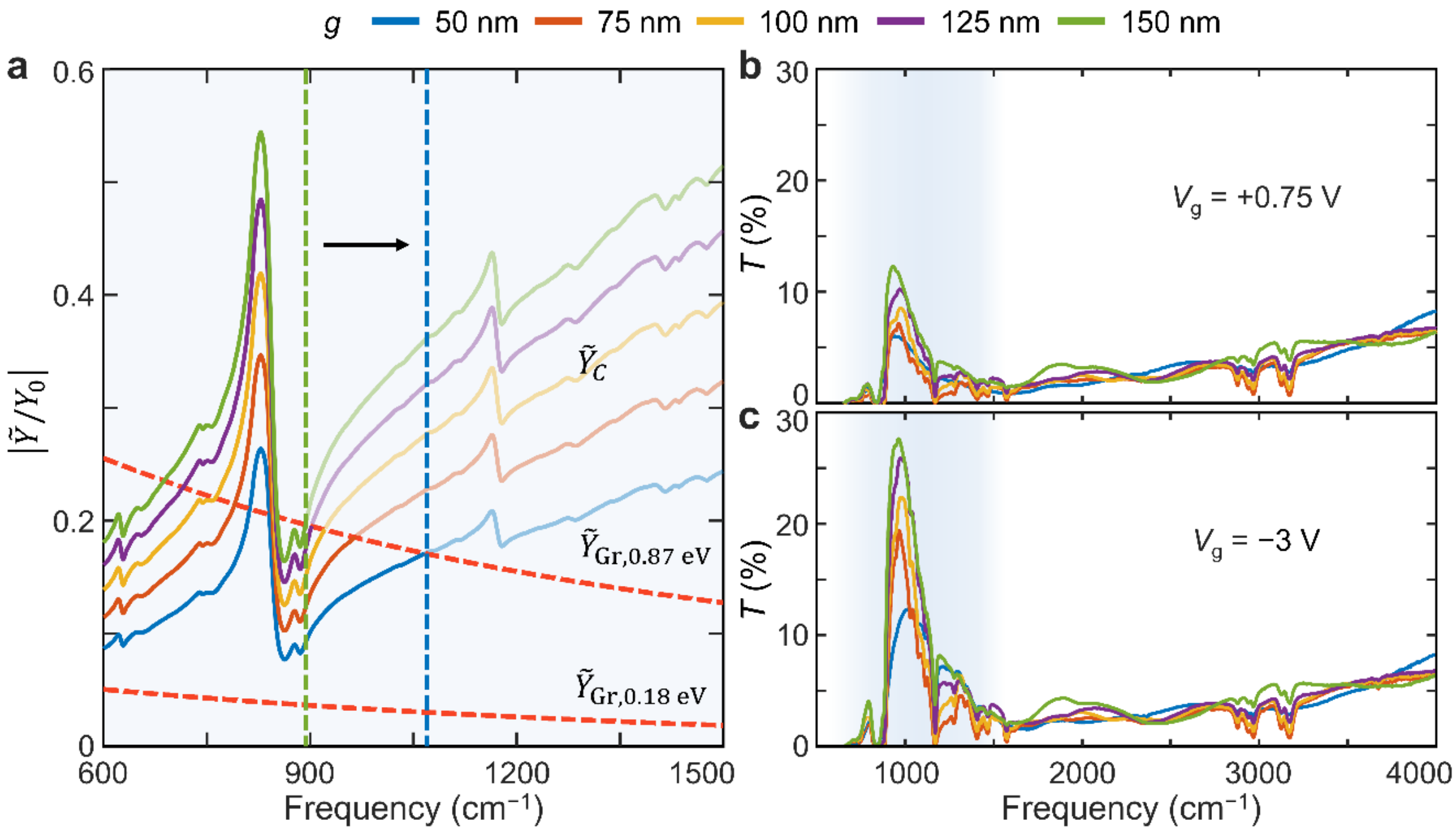

Figure S26. Gap dependence on LC resonance. a Calculated normalized admittance $|\tilde{Y}/Y_0|$ of the capacitive metal antenna ($\tilde{Y}_C$, solid lines) and graphene ($\tilde{Y}_{Gr}$, red dashed lines) for varying gap sizes (p = 3 μm, g = 50–150 nm). Two $\tilde{Y}_{Gr}$ lines represent the CNP and E_F = 0.87 eV case, respectively. **b, c** Measured transmission spectra for TM-polarized light of devices with a fixed period of 3 μm and gap sizes ranging from 50 nm to 150 nm at an applied gate voltage of (b) −3 V and (c) +0.75 V.

A similar blueshift is observed as the gap size g decreases, as shown in Figure S26a. To understand this, we examine the gap dependence of the capacitive term $\tilde{Y}_C(g) = A\omega g \ln p/g$, where A is a constant related to material permittivity. The derivative of this term with respect to g is given by:

$$\frac{\partial \tilde{Y}_C}{\partial g} = A\omega\left(\ln\left(\frac{p}{g}\right) - 1\right). \quad \text{(S20)}$$

Given that our metasurfaces operate in the large period-to-gap ratio regime ($g \ll p$), the value of ln(p/g) is significantly greater than 1. Therefore, $\partial \tilde{Y}_C/\partial g \gg 0$, meaning that a decrease in g directly leads to a decrease in the capacitive admittance $|\tilde{Y}_C|$. This reduction in $|\tilde{Y}_C|$ results in a higher resonance frequency.

Furthermore, the simultaneous increase in the p/g ratio as g decreases further enhances the total surface admittance $|Y_S|$, leading to the overall decrease in transmission magnitude observed in Figure S26b and S26c.

Supplementary Note 15. Optimization of regularization weights via genetic algorithm

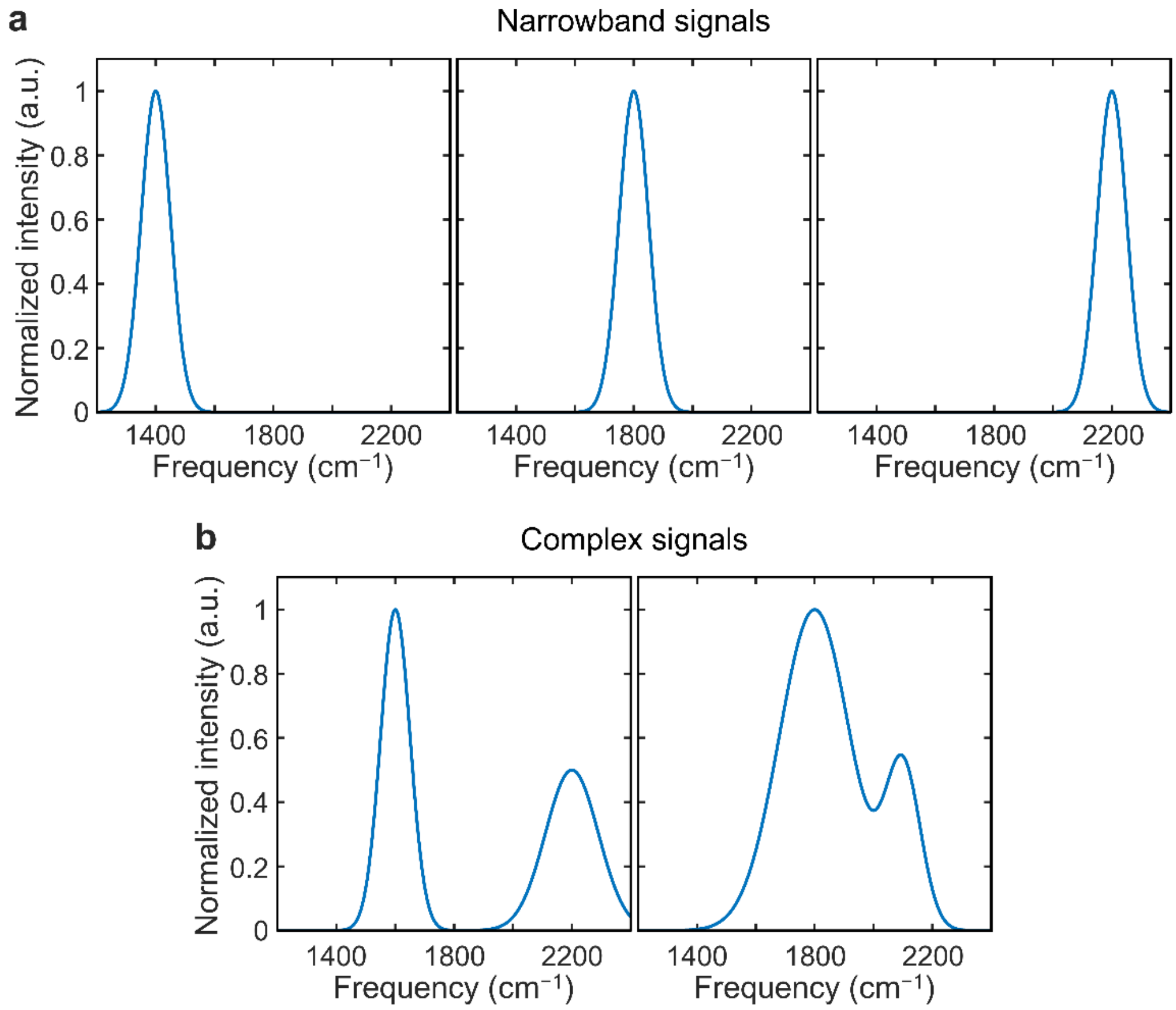

Figure S27. Training dataset utilized for the genetic algorithm (GA) optimization. a Three narrowband Gaussian signals with a standard deviation of 50 cm^{-1}, centered at 1400, 1800, and 2200 cm^{-1}. **b** Two complex broadband signals included in the training set to evaluate the robustness of the algorithm against varied spectral shapes.

To optimize the six regularization weights (α_i, β_i, γ_i for $i = 1, 2$) in Equation 5 of the main text, we employed a genetic algorithm. The optimization was performed using a predefined training dataset comprising five representative spectra, as shown in Figure S27. This dataset includes three narrowband Gaussian signals (Figure S27a) and two complex broadband signals (Figure S27b). The narrowband training signals have a standard deviation of 50 cm^{-1} with center frequencies positioned at 1400, 1800, and 2200 cm^{-1}. Note that these center frequencies are deliberately chosen to be different from those evaluated in Figure 4c of the main text to ensure that the optimized parameters yield generalized reconstruction performance rather than simply overfitting to specific targets.

To guarantee that the optimized weights are robust against experimental uncertainties, we simulated realistic measurement conditions by adding 0.5% additive white Gaussian noise (AWGN) to the training signals. For rigorous reproducibility, fixed random seeds were used to generate the noise

profiles. During the GA optimization, each set of weights (optimizing parameter) was evaluated based on the reconstruction error. The reconstruction performance was quantified using a customized loss function which accounts for both the absolute spectral error and the gradient error to preserve the shape of the spectra. The loss function is defined as $Loss = MSE + 0.5 \times MSE_{\mathrm{grad}}$, where the normalized mean squared error (MSE) and the gradient mean squared error ($\mathrm{MSE_{grad}}$) are calculated using the L_2-norm ($\|\cdot\|_2$) as follows:

$$MSE = \frac{\|\Phi_0 - \Phi_{\mathrm{recon}}\|_2}{\|\Phi_0\|_2}, \tag{S21}$$

$$MSE_{\mathrm{grad}} = \frac{\|D_1\Phi_0 - D_1\Phi_{\mathrm{recon}}\|_2}{\|D_1\Phi_0\|_2}. \tag{S22}$$

Here, Φ_0 and Φ_{recon} represent the original input spectrum and the reconstructed spectrum, respectively, and D_1 denotes the first-order differential operator. If the convex optimization solver (CVX) failed to converge and yielded NaN values for a given set of weights, a severe penalty was applied by assigning an extremely high loss value (10^5) to heavily penalize and eliminate that specific weight combination from the gene pool.

To formulate a robust Figure of Merit (FoM) for the GA, a minimax approach combined with multiple noise trials was implemented. For a given set of weights, we reconstructed all five training spectra and identified the maximum loss value (i.e., the worst-performing case among the five). To prevent the weights from being biased by a specific noise pattern, this process was independently repeated for two different random noise trials. The final FoM for the GA was defined by averaging the maximum loss values from the two trials and multiplying by −1, allowing the GA to search for the optimal weights by maximizing the FoM. By utilizing this FoM, the genetic algorithm effectively minimizes the worst-case reconstruction error across varied signal types and noise conditions, leading to highly stable and reliable regularization parameters.

Supplementary Note 16. Additional numerical analysis of the computational spectrometer

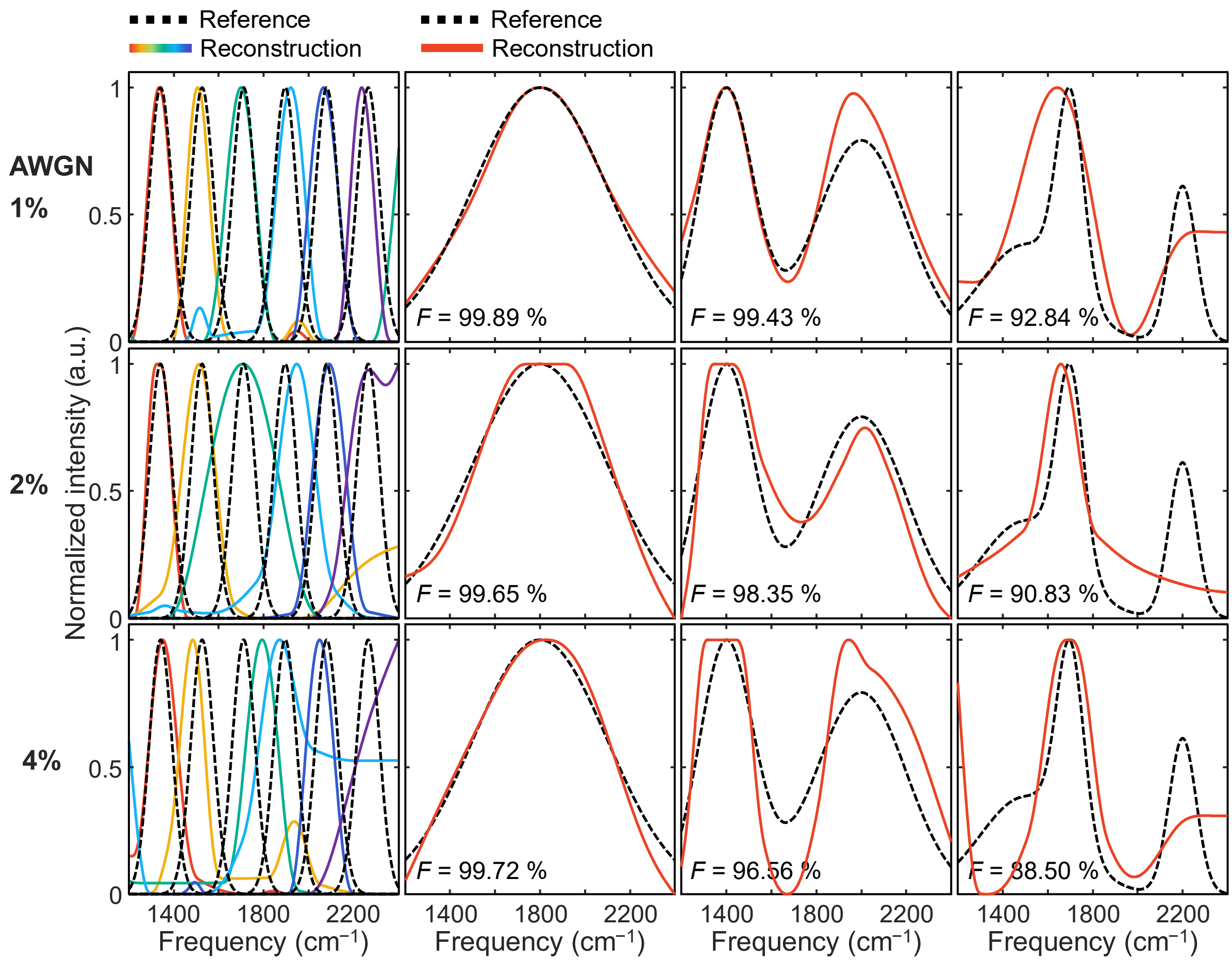

Figure S28. Spectral reconstruction against elevated additive white Gaussian noise (AWGN). Reconstructed spectra evaluated at AWGN levels of 1% (top), 2% (middle), and 4% (bottom). The left column shows results for narrowband Gaussian signals, while the remaining columns display broadband and complex multi-peak signals. The reference spectra and the reconstructed spectra are plotted as black dashed and colored solid lines, respectively.

We additionally investigated the robustness of the computational spectrometer under higher additive white Gaussian noise (AWGN) levels of 1%, 2%, and 4%. As shown in Figure S28, the reconstruction performance progressively degrades as the noise level increases. At 1% AWGN, the reconstructed spectra remain in good agreement with the target spectra, with the lowest reconstruction fidelity of 91.37% among the narrowband signals. Although some fine spectral features begin to disappear for the most complex broadband spectrum, the narrowband spectra and the simpler broadband spectra are still reconstructed accurately. At 2% AWGN, the lowest reconstruction fidelity decreases to

78.95%. While the peak positions of the narrowband spectra are still reproduced reasonably well, noticeable broadening of the reconstructed peaks leads to significant deviations in the spectral linewidths. When the noise level is further increased to 4% AWGN, the lowest reconstruction fidelity decreases to 61.00%, and both the peak positions and linewidths of the narrowband spectra become significantly distorted. Under this condition, only the simplest broadband spectrum with a single broad peak can be reconstructed reliably. These results indicate that the proof-of-concept computational spectrometer presented in this work is sensitive to the signal-to-noise ratio under higher noise conditions, making reliable quantitative spectral reconstruction difficult at 4% AWGN.

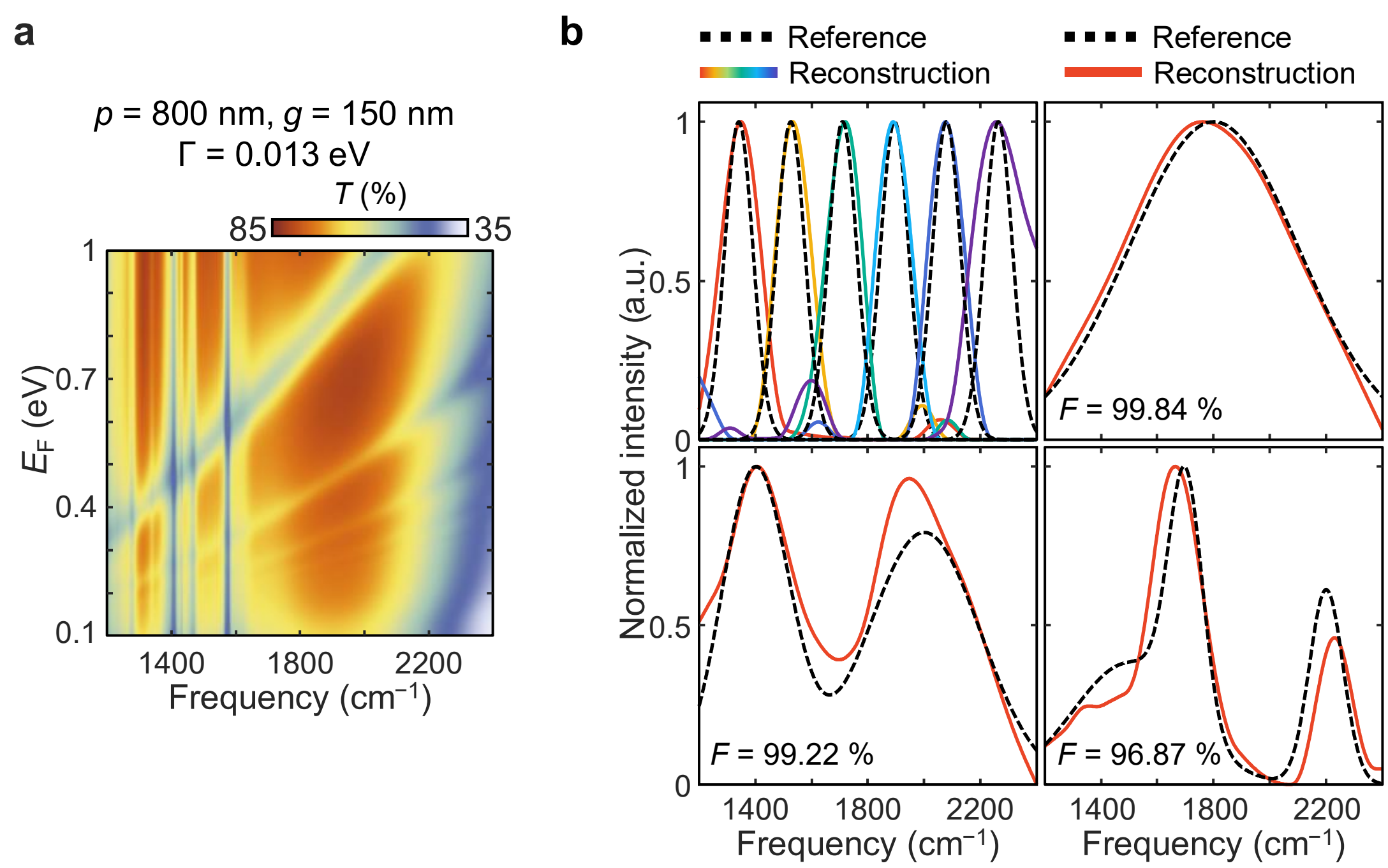

Figure S29. Spectral reconstruction using an optimized computational spectrometer under 4% AWGN. a Simulated transmission matrix of the optimized metasurface. **b** Comparison between the target spectra (dashed lines) and the reconstructed spectra (solid lines) for representative narrowband and broadband signals at AWGN level of 4%.

While high noise levels of 4% AWGN present reconstruction challenges for the proof-of-concept device, such limitation can be effectively overcome by incorporating a relaxed graphene scattering rate (Γ = 0.013 eV) and appropriate structural parameters (p = 800 nm, g = 150 nm). For this modified metasurface design, the regularization parameters (α_i, β_i, γ_i) were re-optimized using the genetic algorithm on the same training set. As shown in Figure S29a, the optimized structure exhibits a significantly more diverse transmission matrix. This improvement arises from the combined effects of the reduced graphene scattering rate, which accentuates the LC-resonance-induced peak shift, and the strengthened contribution of graphene plasmon resonance, together yielding a highly diverse transmission matrix. Consequently, the transmission responses become more distinguishable over the

entire Fermi-level tuning range, providing a more informative measurement basis for computational spectral reconstruction. The corresponding reconstruction results under 4% AWGN are shown in Figure S29b. Despite the substantially higher noise level, the optimized structure successfully reconstructs the reference spectra, with fidelities exceeding approximately 90% for the narrowband spectra and 97% for the broadband spectra. These findings further suggest that optimizing the metasurface geometry together with improving graphene quality can substantially enhance spectral reconstruction performance under realistic noise conditions.

Supplementary Note 17. Polarization-independent 2D grating metasurface design

The two-dimensional grating with $p_x = p_y = 2$ μm and $g_x = g_y = 50$ nm were simulated under normal incidence, as illustrated in Figure S30a. As shown in Figure S30b, the transmission spectra of the simulated two-dimensional grating closely follow the TM response of its one-dimensional counterpart at both $E_F = 0.18$ eV and $E_F = 0.87$ eV, whereas the TE response of the one-dimensional grating exhibits almost no gate-dependent modulation. Consequently, the modulation efficiency under unpolarized illumination is reduced in the one-dimensional grating because the nearly constant TE transmission contributes to both T_{min} and T_{max}. In contrast, owing to its fourfold rotational symmetry, the two-dimensional grating maintains strong modulation for both orthogonal polarizations, thereby preserving a high modulation efficiency under unpolarized illumination. As shown in Figure S30c, the simulated two-dimensional grating achieves a maximum modulation efficiency of 74.0% at 1651 cm^{-1}, compared with 63.3% at 1350 cm^{-1} for the unpolarized response of the one-dimensional grating. These results demonstrate that the two-dimensional grating maintains a high modulation efficiency under unpolarized illumination by eliminating the polarization dependence of the one-dimensional grating.

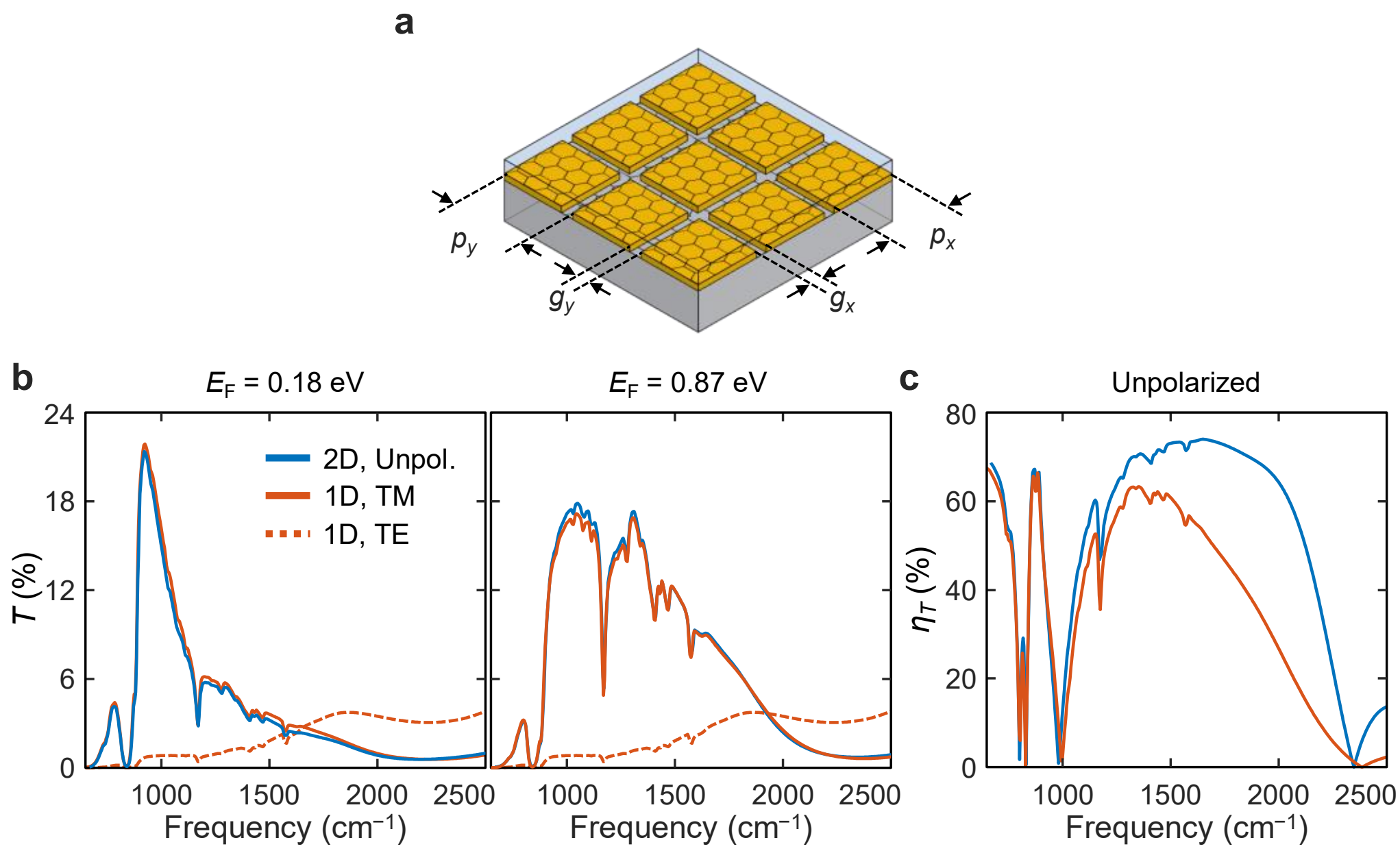

Figure S30. Simulation results of polarization-independent transmission modulation using a two-dimensional grating metasurface. a Schematic of the two-dimensional grating metasurface. **b** Simulated transmission spectra of the two-dimensional grating ($p_x = p_y = 2$ μm and $g_x = g_y = 50$ nm) under unpolarized illumination compared with the TM- and TE-polarized responses of the one-dimensional grating at $E_F = 0.18$ eV and $E_F = 0.87$ eV. **c** Simulated transmission modulation efficiency under unpolarized illumination for the one-dimensional and two-dimensional grating metasurfaces.

Motivated by this concept, we had previously fabricated and characterized a two-dimensional grating metasurface with a period of 2 μm and a gap of 50 nm. Although the simulations predict that the two-dimensional geometry should maintain a modulation efficiency comparable to that of the one-dimensional grating while eliminating polarization dependence, the fabricated device exhibited a somewhat degraded modulation performance, as shown in Figure S31. Specifically, the maximum transmission modulation efficiency was approximately 60%, which is about 10 percentage points lower than that of the one-dimensional grating device with the same period (p = 2 μm) under TM polarization reported in this work. We attribute this degradation primarily to fabrication imperfections associated with defining the narrow two-dimensional gap structures, which are considerably more challenging to fabricate than one-dimensional gratings. We therefore expect that, with further improvements in nanofabrication accuracy, a two-dimensional grating geometry could achieve efficient polarization-independent transmission modulation while maintaining a high modulation efficiency.

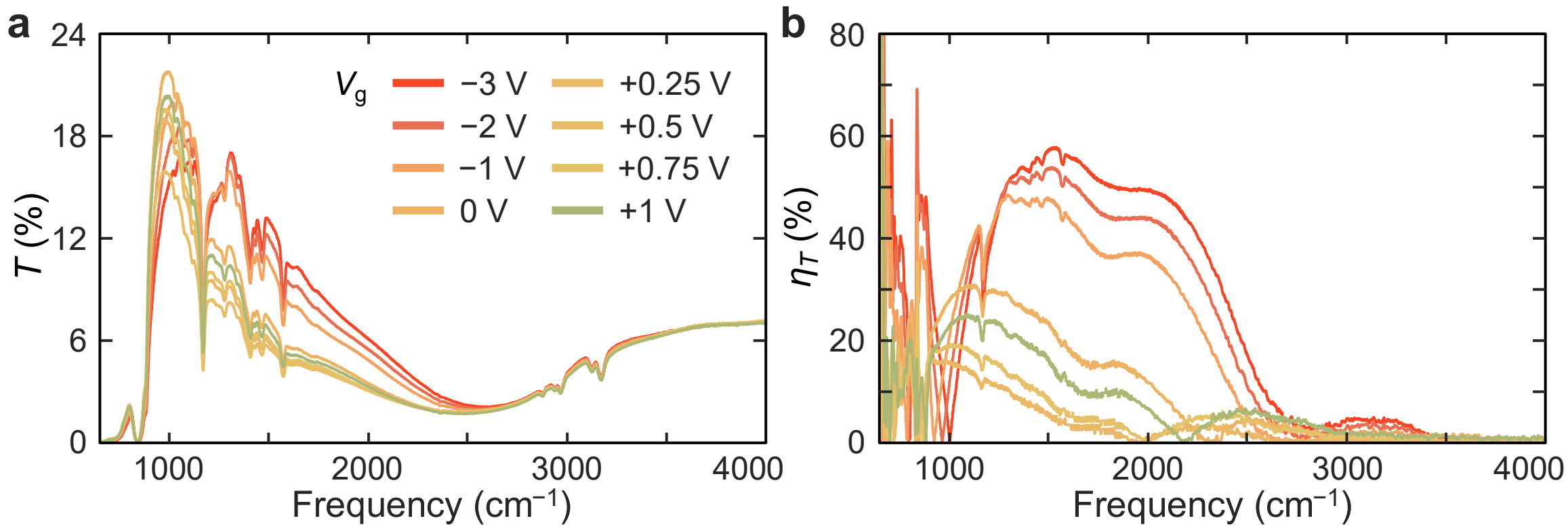

Figure S31. Experimental characterization of the fabricated 2D grating metasurface. a Measured transmission spectra at gate voltages ranging from −3 V to +1 V. **b** Corresponding transmission modulation efficiency calculated using the transmission spectrum at +1 V as the reference.

Supplementary Note 18. Extension toward LWIR operation and fast solid-state gating using BaF_2 and HfO_2

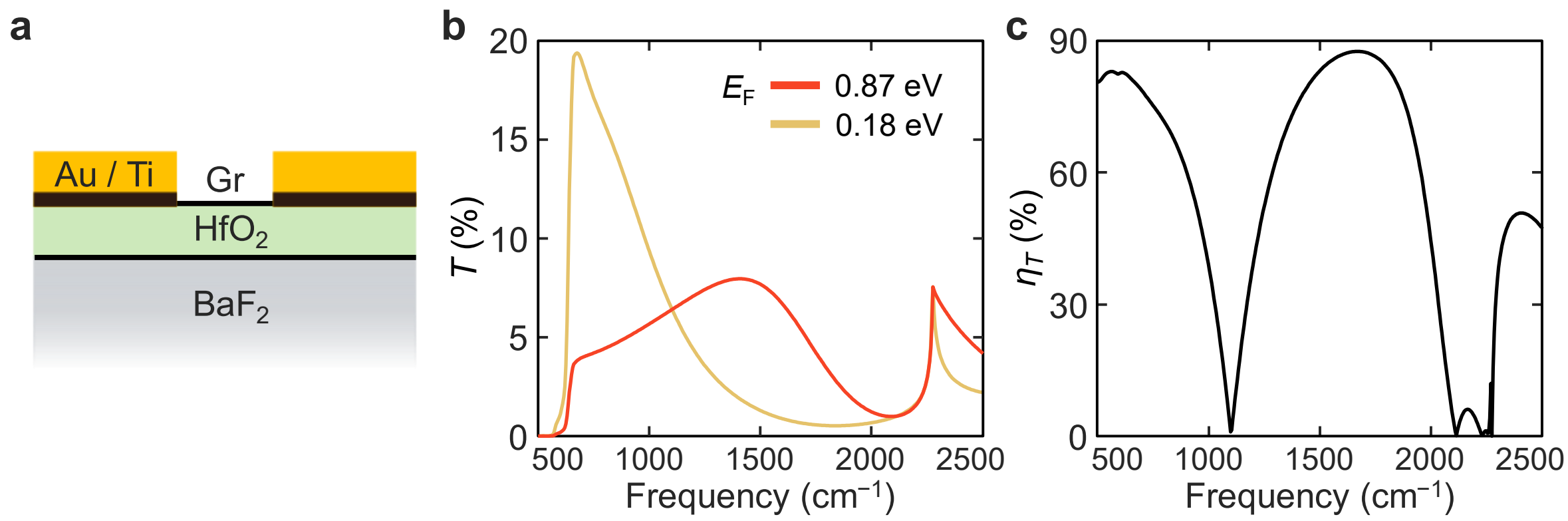

Figure S32. Simulation of the proposed metasurface using a solid-state HfO_2 gate dielectric and a BaF_2 substrate. a Schematic of the graphene metasurface on a BaF_2 substrate with HfO_2 gate oxide. **b** Calculated transmission spectra at E_F = 0.18 eV and E_F = 0.87 eV. **c** Corresponding transmission modulation efficiency.

To evaluate the feasibility of extending the operational bandwidth toward the long-wave infrared region, we performed additional RCWA simulations by replacing the ionic gel and CaF_2 substrate with a solid-state HfO_2 gate dielectric and a BaF_2 substrate while maintaining the same metasurface geometry (p = 3 μm and g = 50 nm), graphene mobility, and Fermi-level. The refractive index data of HfO_2 and BaF_2 are referenced from M. R.[16]. The simulated structure consisted of a BaF_2 (500 μm)/Gr (0.34 nm)/HfO_2 (10 nm)/Ti (2 nm)/Au (10 nm)/graphene metasurface, as shown in Figure S32a.

The calculated transmission spectra and corresponding modulation efficiency are presented in Figures S32b and S32c. The proposed structure exhibits a maximum transmission modulation efficiency of 87.7% at 1670 cm^{-1}, corresponding to a transmission change from 0.7% to 5.6%. In addition, the transmission modulation efficiency reaches 80.5% at 670 cm^{-1} under the charge-neutrality condition, while high absolute transmission is maintained, with transmission values of 19.4% and 3.8% at the two bias states, respectively. These results demonstrate that replacing the ionic gel and CaF_2 substrate with a solid-state HfO_2 gate dielectric and a BaF_2 substrate enables efficient transmission modulation over a broader spectral range extending toward the long-wave infrared region while maintaining a high absolute transmission at lower frequencies. Although these simulation results are promising, the practical realization of this architecture remains challenging because it requires the integration of a

high-quality ultrathin HfO_2 gate dielectric with graphene as well as reliable nanofabrication on a BaF_2 substrate.

Supplementary Note 19. Comparison with previously reported electrically tunable transmissive mid-infrared modulators

To provide a comprehensive comparison with previously reported electrically tunable transmissive mid-infrared modulators, Table S2 summarizes the absolute maximum transmittance (T_{max}), absolute transmission modulation (ΔT), relative modulation depth (η_T), operating bandwidth, driving voltage, and device architecture for representative experimentally demonstrated electrically tunable transmissive mid-infrared modulators.

The comparison reveals that the previously reported graphene modulator by Lan *et al*. achieves a high modulation depth of 41% while maintaining nearly 100% transmittance through the use of triple-layer graphene nanoribbons and asymmetric light–plasmon coupling. In contrast, our device employs a simple architecture consisting of an unpatterned monolayer graphene sheet integrated with a metallic slit metasurface.

To provide a fair comparison, we included two representative device geometries from this work. The device with p = 3 μm and g = 50 nm exhibits the highest modulation efficiency of 73.3%, which is the highest relative modulation efficiency among the compared experimentally demonstrated transmissive graphene modulators. This device, however, operates with relatively low absolute transmittance, highlighting the trade-off between modulation efficiency and transmitted optical power.

On the other hand, the device with p = 1 μm and g = 50 nm simultaneously achieves a relatively high transmittance ($T_{max} \approx 38.9\%$), a large absolute transmission modulation ($\Delta T \approx 17\%$), and a broad operating bandwidth of 1949 cm^{-1}. Compared with the work of Lan *et al*., the p = 1 μm device provides a substantially broader operating bandwidth while maintaining a relatively large absolute transmission modulation using only an unpatterned monolayer graphene sheet.

In addition, the proposed device operates with a gate-voltage swing of only 3.75 V, which is the lowest among the compared experimentally demonstrated transmissive mid-infrared modulators and is significantly lower than the tens to hundreds of volts required by most previously reported devices.

Finally, we note that experimentally demonstrated electrically tunable transmissive mid-infrared modulators based on alternative material platforms, such as black phosphorus, remain relatively limited in number and generally exhibit substantially smaller modulation depths. The added comparison demonstrates that the proposed device offers a competitive combination of high modulation efficiency, large absolute transmission modulation, broad operating bandwidth, low operating voltage, and a simple device architecture.

Reference	Architecture	T_{max}	Absolute ΔT	η_T	Bandwidth (FWHM)	ΔV
[17]	EOT slit + Gr ribbon	10.5%	3%	28.6%	100 cm^{-1}	350 V
[18]	Tri-Gr ribbon + Ionic gel	100%	41%	41%	230 cm^{-1}	4 V
[19]	Gr ribbon + nano gold antenna	82%	14%	17%	80 cm^{-1}	80 V
[20]	BP transistor	Not reported	Not reported	3%	500 cm^{-1}	300 V
This work 1 (p = 1 µm, g = 50 nm)	Gr + Au slit + Ionic gel	38.9%	17%	**44%**	**1949 cm^{-1}**	3.75 V
This work 2 (p = 3 µm, g = 50 nm)	Gr + Au slit + Ionic gel	11.9%	6%	**73.3%**	**742 cm^{-1}**	3.75 V

Table S2. Comparison of representative experimentally demonstrated electrically tunable transmissive mid-infrared modulators. T_{max}, ΔT, and η_T denote the independently determined maximum values of the corresponding performance metrics and therefore do not necessarily occur at the same operating frequency.

References

[1] Min, K.-J., et al., *Asymmetric electron-hole decoherence in ion-gated epitaxial graphene.* Scientific reports, 2017. **7**(1): p. 12130.

[2] Sha, M., et al., *Double-layer formation of [Bmim][PF6] ionic liquid triggered by surface negative charge.* Langmuir, 2010. **26**(15): p. 12667–12672.

[3] O'Mahony, A.M., et al., *Effect of water on the electrochemical window and potential limits of room-temperature ionic liquids.* Journal of Chemical & Engineering Data, 2008. **53**(12): p. 2884–2891.

[4] Polat, E.O. and C. Kocabas, *Broadband optical modulators based on graphene supercapacitors.* Nano letters, 2013. **13**(12): p. 5851–5857.

[5] Kaiser, W., et al., *Infrared Properties of Ca F 2, Sr F 2, and Ba F 2.* Physical Review, 1962. **127**(6): p. 1950.

[6] Li, H., *Refractive index of alkaline earth halides and its wavelength and temperature derivatives.* Journal of physical and chemical reference data, 1980. **9**(1): p. 161–290.

[7] Brandt, T., et al., *Temperature-and frequency-dependent optical properties of ultrathin Au films.* Physical Review B—Condensed Matter and Materials Physics, 2008. **78**(20): p. 205409.

[8] Olmon, R.L., et al., *Optical dielectric function of gold.* Physical Review B—Condensed Matter and Materials Physics, 2012. **86**(23): p. 235147.

[9] Rakić, A.D., et al., *Optical properties of metallic films for vertical-cavity optoelectronic devices.* Applied optics, 1998. **37**(22): p. 5271–5283.

[10] Hanson, G.W., *Dyadic Green's functions and guided surface waves for a surface conductivity model of graphene.* Journal of Applied Physics, 2008. **103**(6).

[11] Falkovsky, L.A. *Optical properties of graphene*. in *Journal of Physics: conference series*. 2008.

[12] Azzam, R.M., N.M. Bashara, and S.S. Ballard, *Ellipsometry and polarized light*. 1978, American Institute of Physics.

[13] Centurioni, E., *Generalized matrix method for calculation of internal light energy flux in mixed coherent and incoherent multilayers.* Applied optics, 2005. **44**(35): p. 7532–7539.

[14] Hugonin, J.P. and P. Lalanne, *Reticolo software for grating analysis.* arXiv preprint arXiv:2101.00901, 2021.

[15] Han, S., et al., *Complete complex amplitude modulation with electronically tunable graphene plasmonic metamolecules.* ACS nano, 2020. **14**(1): p. 1166–1175.

[16] Querry, M.R., *Optical constants of minerals and other materials from the millimeter to the ultraviolet*. 1998: Chemical Research, Development & Engineering Center, US Army Armament ….

[17] Kim, S., et al., *Electronically tunable extraordinary optical transmission in graphene plasmonic ribbons coupled to subwavelength metallic slit arrays.* Nature communications, 2016. **7**(1): p. 12323.

[18] Lan, G., et al., *Enhanced Asymmetric Light-Plasmon Coupling in Graphene Nanoribbons for High-Efficiency Transmissive Infrared Modulation.* Laser & Photonics Reviews, 2024. **18**(1): p. 2300469.

[19] Feinstein, M.D. and E. Almeida, *Hybridization of graphene-gold plasmons for active control of mid-infrared radiation.* Scientific reports, 2024. **14**(1): p. 6733.

[20] Peng, R., et al., *Midinfrared electro-optic modulation in few-layer black phosphorus.* Nano letters, 2017. **17**(10): p. 6315–6320.
